# Differential Expression of miRNAs in Response to Topping in Flue-Cured Tobacco (*Nicotiana tabacum*) Roots

**DOI:** 10.1371/journal.pone.0028565

**Published:** 2011-12-14

**Authors:** Hongxiang Guo, Yunchao Kan, Weiqun Liu

**Affiliations:** 1 The Key Lab of National Tobacco Cultivation, College of Life Sciences, Henan Agricultural University, Zhengzhou, China; 2 China-UK NYNU-RRes Joint Lab of Insect Biology, Nanyang Normal University, Nanyang, China; University of Arizona, United States of America

## Abstract

**Background:**

Topping is an important cultivating measure for flue-cured tobacco, and many genes had been found to be differentially expressed in response to topping. But it is still unclear how these genes are regulated. MiRNAs play a critical role in post-transcriptional gene regulation, so we sequenced two sRNA libraries from tobacco roots before and after topping, with a view to exploring transcriptional differences in miRNAs.

**Methodology/Principal Findings:**

Two sRNA libraries were generated from tobacco roots before and after topping. Solexa high-throughput sequencing of tobacco small RNAs revealed a total of 12,104,207 and 11,292,018 reads representing 3,633,398 and 3,084,102 distinct sequences before and after topping. The expressions of 136 conserved miRNAs (belonging to 32 families) and 126 new miRNAs (belonging to 77 families) were determined. There were three major conserved miRNAs families (nta-miR156, nta-miR172 and nta-miR171) and two major new miRNAs families (nta-miRn2 and nta-miRn26). All of these identified miRNAs can be folded into characteristic miRNA stem-loop secondary hairpin structures, and qRT-PCR was adopted to validate and measure the expression of miRNAs. Putative targets were identified for 133 out of 136 conserved miRNAs and 126 new miRNAs. Of these miRNAs whose targets had been identified, the miRNAs which change markedly (>2 folds) belong to 53 families and their targets have different biological functions including development, response to stress, response to hormone, N metabolism, C metabolism, signal transduction, nucleic acid metabolism and other metabolism. Some interesting targets for miRNAs had been determined.

**Conclusions/Significance:**

The differential expression profiles of miRNAs were shown in flue-cured tobacco roots before and after topping, which can be expected to regulate transcripts distinctly involved in response to topping. Further identification of these differentially expressed miRNAs and their targets would allow better understanding of the regulatory mechanisms for flue-cured tobacco response to topping.

## Introduction

Tobacco is one of the most important economic crops that leaves are the main product. To maximize leaf production and encourage leaf ripening, topping (removal of the flowering head and young leaves) is an essential cultivating measure for flue-cured tobacco which switches the plant from reproductive to vegetative phase. Previous reports showed that there were many responses of flue-cured tobacco to topping [1], [2]. It had been proved that topping might act as a wounding signal to induce a decrease in IAA synthesis and an increase in JA content [3], which in turn affected plants growth, sink-source relation, root secondary growth and metabolism [4].

Nicotine, a secondary metabolite synthesized in tobacco roots, acts as a unique alkaloid in tobacco and is an important quality factor for tobacco. The increase in nicotine synthesis after topping is one of the typical responses of flue-cured tobacco to topping, therefore, the optimal plant material can be provided for studying nicotine synthesis by topping [5], [6]. Protein patterns in roots of flue-cured tobacco before and after topping were analyzed by two-dimensional electrophoresis (2-DE) [7]. Twenty-six differentially expressed proteins were revealed, and four differential proteins were enzymes possibly involved in nicotine biosynthesis. However, nicotine biosynthesis is involved in a rather complicated network and the related regulating factors in the network had not been found, it still needs to be further studied that how topping promotes nicotine biosynthesis in tobacco roots.

Root systems hold the plant upright, absorb water and nutrition for plant growth and development, produce the hormone cytokinin, and generate secondary metabolites. The change in roots development after topping is also an important response of flue-cured tobacco to topping. It had been proved that topping could increase the activity, number and biomass of the roots [8]. However, the regulation mechanism involved in increased roots development in response to topping is still unclear.

To clarify the response of flue-cured tobacco roots to topping, the suppression subtractive hybridization (SSH) library before and after topping was successfully constructed and 273 high quality expressed sequence tags (ESTs) were acquired [9]. These ESTs mainly involved in alkaloid biosynthesis (4%), plant hormone metabolism (3%), signaling/transcription (18%), stress/defense (32%), protein metabolism (9%), carbon metabolism (6%), other metabolism (15%) and function unknown (13%). The results showed that a large number of genes in flue-cured tobacco roots were regulated in response to topping, but their regulation mechanism is still unknown.

Regulation of gene expression can occur at both transcriptional and post-transcriptional levels. In recent years, the discovery of numerous microRNAs (miRNAs) has increased interest in post-transcriptional gene expression regulation during development and other biological processes. Plant miRNA-guided gene regulation has been shown to be involved in multiple plant processes including response to environmental stresses, developmental transitions, phase switch from vegetative growth to reproductive growth, organ polarity, tissue (leaf, root, stem, and flower) differentiation and development, auxin signaling and RNA metabolism [10], [11]. Several miRNA families had been reported to be involved in root development modulation in both Arabidopsis and rice. Consistent with recent notion that numerous signaling pathways are implicated in root development, these miRNAs are implicated in auxin signaling, nutrition metabolism, or stress response and have potential role in mediating the signal interactions. Some miRNA families, such as miR160, miR164, miR167, and miR390, mediated auxin signaling in roots, and they had been demonstrated to be involved in root cap formation, lateral root development, or adventitious rooting [12]. MiR395 had been recognized as a key regulator in sulphate metabolism in both Arabidopsis and rice [13]. MiR398 was found to be involved in copper and zinc homeostasis through its post-transcriptional effects on CSD (copper/zinc superoxide dismutase) genes [14], [15]. MiR399 was a well-characterized modulator implicated in phosphate starvation response in Arabidopsis [16], [17], and the juvenile-to-adult transition in Arabidopsis is mediated by sequentially operating miR156 and miR172 [18]. In rice, miR169 g is induced by drought, and the induction is more prominent in roots than in shoots [19]. It was also reported that miR163 was involved in secondary metabolism in Arabidopsis [20].

Topping is an important and essential cultivating measure for tobacco, and miRNA-guided post-transcriptional regulation might be involved in the response of tobacco to topping. Therefore, the identification of miRNAs could be a critical step to facilitate our understanding of the molecular regulation mechanisms of tobacco response to topping. There have been some studies to discover miRNAs and analyze their functions in tobacco [21], [22], but no studies have been reported on discovering tobacco roots miRNAs before and after topping. In the present study, two sRNA libraries were generated from tobacco roots before and after topping, and a large number of miRNAs (136 conserved miRNAs and 126 new miRNAs) from tobacco roots were identified. The targets of miRNAs which change markedly (>2 folds) belong to 53 miRNA families and have different biological functions including development, response to stress, response to hormone, N metabolism, C metabolism, signal transduction, nucleic acid metabolism and other metabolism. The results indicated that these differential miRNAs play vital roles in the response to topping. Further identification of these differentially expressed miRNAs would allow better understanding of the regulatory mechanisms for tobacco response to topping. Moreover, since miRNAs are evolutionarily conserved across species, our results may become a useful resource for miRNA studies in other plants.

## Materials and Methods

### Plant materials

The flowering head and young leaves were removed when the first flower of inflorescence is blossoming. Roots tissues were collected from tobacco at 24 h before and after topping. After collection, all the samples were immediately frozen in liquid Nitrogen and stored at −80°C until used.

### RNA isolation and RNA sequencing

Total RNA was isolated from roots tissue using Trizol agent (TaKaRa, Dalian, China), according to the manufacturer's instructions. MiRNA cloning was performed as described previously by Sunkar and Zhu [23]. Briefly, 0.5 M NaCl and 10% PEG8000 were used to precipitate and enrich RNAs with low molecular weight. Next, a 15% polyacrylamide denaturing gel was employed to separate the low-molecular weight RNA. During gel electrophoresis, about 100 µg of total RNA was applied to the gel and two labeled RNA oligonucleotides, approximately 18 and 26 nt in length, were used as size standards. After gel electrophoresis, small RNAs with 18–26 nt were excised from the gel and eluted with 0.4 M NaCl overnight at 4°C. The RNA was dephosphorylated using alkaline phosphatase (New England Biolabs, Beijing China) and recovered by ethanol precipitation. The isolated small RNAs were then sequentially ligated to 5′- and 3′-chimeric oligonucleotide adapters, reversely transcribed, and amplified by PCR. Finally, Solexa sequencing technology was employed to sequence the small RNAs from tobacco roots samples (Beijing Genomics Institute, Shenzhen, Guangdong, China).

### Bioinformatic analysis of identified miRNA

The raw sequences were processed as the following method. Only small RNA reads that passed the Illumina pipeline quality control and contained clear adaptor sequences were considered good reads for further processing. After adaptor sequence was trimmed, clean small RNA reads of 18nt or more were combined into unique sequences. Reads that match known plant repeats, rRNAs, tRNAs, snRNAs, and snoRNAs were removed. The unique small RNA reads were used to do a Blastn search against the four genomic sequence resources: 317, 49 tobacco ESTs (www.ncbi.nlm.nih.gov), 300,158 tobacco genomic sequences (ftp.solgenomics.net/tobacco_genome), 44,561 tobacco shotgun sequences (ftp.tigr.org/pub/data/plantta/Nicotiana_tabacum), and 1,420,595 tobacco Genome Survey Sequences (GSS,www.ncbi.nlm.nih.gov). Perfect match was required. Results of these BLASTn searches were then subjected to secondary structure analysis using UNAFOLD version 3.8. We then examined the secondary structure, and a result was considered as a genuine miRNA candidate if it met the miRNA criteria [24]. To identify the conserved miRNAs in tobacco, these miRNA candidates were used to do a Blastn search against the miRNA database (miRBase17.0, http://www.mirbase.org), and only the perfectly matched sequences (number of mismatch<3) were considered to be conserved miRNAs. Except for these conserved miRNAs, other miRNA candidates were considered to be new miRNAs. The sequence reads of each miRNA in two libraries were normalized, and then the fold change of miRNAs between two libraries was obtained.

### miRNA validation

The identified tobacco miRNAs were validated by using quantitative real time PCR (qRT-PCR). In this study, 5 conserved miRNAs (nta-miR171i, nta-miR167d*, nta-miR164a, nta-miR166a*, nta-miR399a) and 2 new miRNAs (nta-miRn47 and nta-miRn49) were validated. The primer for the 5 conserved miRNAs and the 3 new miRNAs were purchased from TaKaRa (Table S5).

The TaKaRa One Step PrimeScript®miRNA cDNA Synthesis Kit (Perfect Real Time) was used in the reverse transcription reaction. The RT-PCR temperature program was adjusted to run for 60 min at 37°C, 5 s at 85°C, and then 4°C until future use. For each miRNA, three biological replicates were performed. After reverse transcription, the products of each reaction were diluted 5 times to avoid potential primer interference in the following qRT-PCR reaction.

Quantitative real time PCR was performed using the TaKaRa SYBR® Premix Ex Taq™ (Perfect Real Time) on a Bio-Rad IQ5 Real-Time PCR Detection System. Each reaction consisted of 2 µL of product from the diluted reverse transcription reaction, 0.5 µL of primers (forward and reverse), 12.5 µL of 2×SYBR® Premix Ex Taq™, and 9.5 µL of nuclease-free water. The reactions were incubated in a 96-well plate at 95°C for 30 s, followed by 40 cycles of 95°C for 5 s, 57°C for 30 s and 72°C for 30 s. After the reactions were completed, the threshold was manually set and the threshold cycle (C_T_) was automatically recorded. The C_T_ is defined as the fractional cycle number at which the fluorescence signal passes the fixed threshold. All reactions were run in three replicates for each sample.

### Prediction of potential target mRNA for tobacco miRNA

MiRNAs regulate gene expression by binding to targeted mRNA sequences in a perfect complementary site, and this makes it possible to predict plant miRNA targets using a homology search. The potential targets mRNA for tobacco miRNAs were predicted using the psRNATarget program (http://bioinfo3.noble.org/psRNATarget/) with default parameters. Nicotiana tabacum (tobacco) SGN unigene database (ftp://ftp.sgn.cornell.edu/unigene_builds/Nicotiana_tabacum.seq) were used as the sequence library for target search. All predicted target genes were evaluated by scoring, and sequences were considered to be miRNA targets if the total score was less than 3.0 points.

## Results

### Small RNA population in tobacco roots

**Figure 1:**
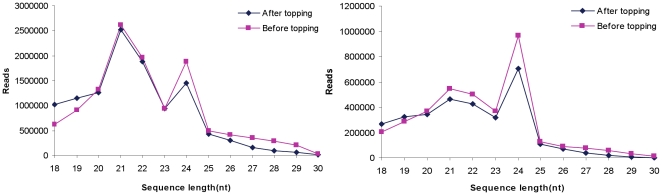
Size distribution of small RNAs in tobacco roots. Left: redundant reads; Right: unique reads.

To identify miRNAs involved in tobacco topping, two sRNA libraries were generated from tobacco roots before and after topping. The two libraries were sequenced by Solexa (Illumina), yielding a total of 12,104,207 and 11,292,018 sRNA raw reads with lengths of 18 to 30nt and consisting of 3,633,398 and 3,084,102 unique sequences (Table 1). After removing rRNA, tRNA, snRNA and snoRNA, a total of 9,979,127 and 9,351,526 small RNA sequences were obtained. This suggests that tobacco roots contain a large and diverse small RNA population. To further compare the average abundance of different sRNAs, we measured the ratio of redundant and unique sequences. After topping, the ratio of redundant and unique conserved miRNA sequences in tobacco roots decreased distinctly (Table 1), which suggests that topping can decrease the abundance of conserved miRNA in tobacco roots. The size distribution of both unique and redundant reads was assessed (Figure 1). From the size distribution of reads, we found that the majority of small RNAs were in the range from 18 to 24nt, and there were two distinct peaks around 21 and 24 nt in the two stages. So 21 and 24 nt small RNAs are the two major size classes, and this result was consistent with those of *Arabidopsis* and *Oryza sativa* [23]. The reads of 24 nt small RNAs in tobacco roots after topping were markedly less than that in tobacco roots before topping, which suggests that 24 nt small RNAs have a critical role in the development of tobacco roots after topping. These observations indicated that the expression of miRNAs and siRNAs significantly altered after topping, suggesting that miRNAs and siRNAs could be involved in the extensive regulation of gene expression in response to topping in tobacco roots.

**Table 1:** SRNAs annotation and distribution.

sRNA class	Before topping			After topping		
	Unique Reads	Redundant Reads	Redundant/Unique	Unique Reads	Redundant Reads	Redundant/Unique
rRNA	121,157	1,206,590	9.96	95,822	1,048,627	10.94
tRNA	63,757	909,903	14.27	37,194	883,721	23.76
snRNA	2,545	5,576	2.19	2,324	5,358	2.31
snoRNA	907	3,011	3.32	814	2,786	3.42
Conserved miRNAs	451	247,850	549.56	470	116,004	246.82
Novel miRNA	221	72,569	328.37	219	65,939	301.09
unannotated	3,444,360	9,658,708	2.80	2,947,259	9,169,583	3.11
total	3,633,398	12,104,207	3.3	3,084,102	11,292,018	3.66

**Figure 2:**
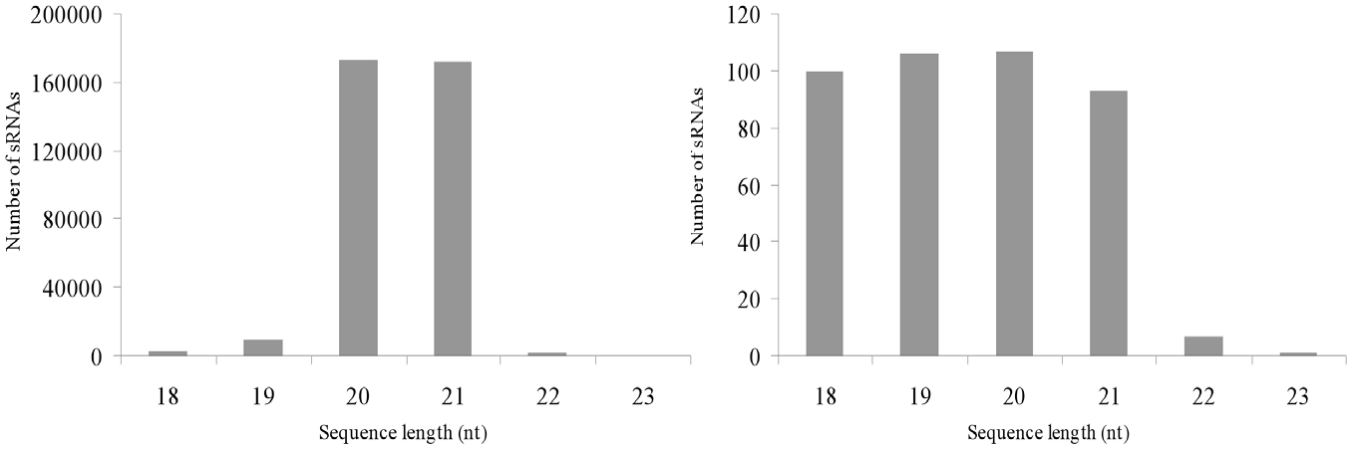
Size distribution of miRNA from tobacco roots. Left: redundant reads; Right: unique reads.

The size distribution of all sRNAs annotated as miRNAs is summarized in Figure 2. Although the sRNAs annotated as miRNAs in the sizes of 18, 19, 20 and 21 nt all had about 93–107 unique reads, the redundant reads of 18 and 19 nt group were surprisingly less than that of 20 and 21 nt group. 20 and 21 nt group had separately 172964 and 172225 redundant reads, which indicated that 20 and 21 nt group are the most abundant miRNA.

### Identifying conserved miRNAs in tobacco roots

**Figure 3:**
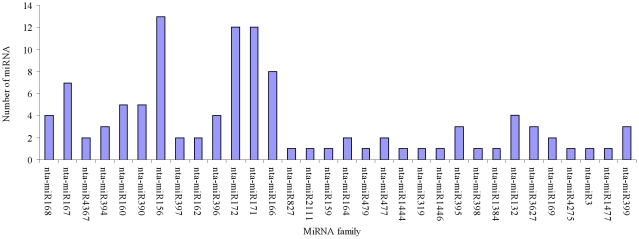
Distribution of conserved miRNA families in tobacco roots.

**Figure 4:**
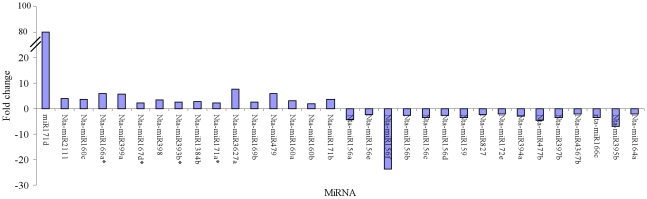
Response of conserved miRNAs in tobacco roots to topping. (Fold change>2).

To identify the conserved miRNAs from tobacco roots, sRNA sequences were compared with the currently known mature plant miRNAs in miRBase. After Blastn searches (number of mismatch<3) and further sequence analysis, 136 sRNAs were identified as conserved miRNAs in tobacco roots (Tables 2 and S1), and we also found 13 miRNA^*^ in our libraries (Tables 3 and S3). The most of the identified miRNA families have been shown to be conserved in a variety of plant species using a comparative genomics-based strategy. For example, miR156 has been found in 41 plant species [25]. These conserved miRNAs belong to 32 families (Figure 3 and Table S1). Of these 32 families, three major families, nta-miR156, nta-miR172 and nta-miR171, were identified to contain 13, 12 and 12 members. All other miRNA families contained fewer than ten members and most only contained 1 or 2 miRNAs per family. The read numbers of all members of nta-miR156 family in the tobacco roots before topping are 2.34 to 23.8 times more than that after topping, and the levels of several miRNAs, such as nta-miR397 (3.34 fold), nta-miR159 (3.52 fold), nta-miR395b (6.85 fold), nta-miR477b (4.56 fold), nta-miR171f (4.39 fold), and nta-miR166c (3.33 fold), were markedly greater in tobacco roots before topping than that after topping. These results suggested that the topping can reduce distinctly the expression levels of some miRNAs and all members of the nta-miR156 family (Figure 4). On the contrary, the read numbers of all members of nta-miR160 family in the tobacco roots after topping are 1.50 to 3.73 times more than that before topping, and the levels of several miRNAs, such as nta-miR479 (5.89 fold), nta-miR2111 (4.02 fold), nta-miR160a (3.08 fold), nta-miR160c (3.73 fold), nta-miR396b (3.73 fold), nta-miR171b (3.73 fold), nta-miR3627a(7.85 fold), nta-miR399a (5.65 fold), nta-miR171d (86.20 fold) and nta-miR398 (3.41 fold), were markedly greater in tobacco roots after topping than that before topping. These results implied that the topping can increase the expression levels of some miRNAs (Figure 4). Of 13 miRNA^*^, nta-miR166^*^(5.95 fold), nta-miR393a^*^ (3.59 fold) and nta-miR393c^*^ (11.19 fold) were markedly greater in tobacco roots after topping than that before topping.

**Table 2:** Conserved miRNAs in tobacco roots.

Family	Mature miRNA	ML	C Reads	T Reads	Total Reads	Fold change
nta-miR168a	TCGCTTGGTGCAGGTCGGGAC	21	10142.6	7722.3	17864.9	−1.31
nta-miR168b	TCGCTTGGTGCAGGTCGGGAT	21	30.2	38.3	68.5	+1.26
nta-miR168c	TCGCTTGGTGCAGGTCGGGACC	22	8.6	15.8	24.4	+1.75
nta-miR167a	TGAAGCTGCCAGCATGATCTA	21	11987.1	6536.2	18523.3	−1.83
nta-miR167b	TATCTGATTGGCGTGGCAAAT	21	7678.2	4404.7	12082.9	−1.74
nta-miR4367a	ACGCAGGAGGGATGATACT	19	169	391.6	560.1	+2.32
nta-miR4367b	TACGCAGGAGAGATGATGCTG	21	725.7	342.1	1067.8	−2.12
nta-miR394a	TTGGCATTCTGTCCACCTCC	20	216	78.8	294.8	−2.72
nta-miR160a	GCGTGCGAGGAGCCAAGCATA	21	1051.8	3241.1	4292.9	+3.08
nta-miR160b	GCGTATGAGGAGCCAAGCATA	21	2816.4	6011.8	8828.2	+2.13
nta-miR160c	CGTATGAGGAGCCAAGCATA	20	6.5	27	33.5	+3.73
nta-miR160d	TGCCTGGCTCCCTGTATGCCA	21	41	27	68	−1.5
nta-miR390a	AAGCTCAGGAGGGATAGCACC	21	665.2	709	1374.2	+1.07
nta-miR390b	AAGCTCAGGAGGGATAGCGCC	21	149	247.6	396.6	+1.66
nta-miR390c	AGCTATGTTGCTCGGACTCTC	21	19.4	18	37.4	−1.07
nta-miR156a	TTGACAGAAGATAGAGAGCAC	21	127864.8	30725	158589.8	−4.16
nta-miR156b	TGACAGAAGAGAGTGAGCACC	21	207.3	83.3	290.6	−2.47
nta-miR156c	TGACAGAAGAGAATGAGCAC	20	7756	2349.8	10105.8	−3.3
nta-miR156d	TTGACAGAAGAGAGAGAGCAC	21	34.6	13.5	48.1	−2.46
nta-miR156e	TTGATAGAAGATAGAGAGCAC	21	54	22.5	76.5	−2.34
nta-miR156f	AGTGACAGAAGAGAGTGAGCA	21	23.8	0	23.8	−23.8
nta-miR397a	TCATCTGCGCTGCACTCAATCA	22	19.4	29.3	48.7	+1.49
nta-miR397b	ATTGAGTGCAGCGTTGATGAA	21	295.9	87.8	383.7	−3.34
nta-miR162a	TCGATAAACCTCTGCATCCAG	21	1043.2	650.5	1693.7	−1.6
nta-miR396a	TTCCACAGCTTTCTTGAA	18	127.4	308.4	435.8	+2.41
nta-miR396b	TTAGAGGAAGGAGAAGTT	18	6.5	27	33.5	+3.73
nta-miR396c	AAGCTGTGGGAAAATATGGCA	21	1056.2	515.4	1571.6	−2.05
nta-miR172a	AGAATCATGATGATGCTGCAT	21	95	56.3	151.3	−1.68
nta-miR172b	GGGAATCTTGATGATGCTGCA	21	103.7	171.1	274.8	+1.64
nta-miR172c	AGAATCTTGATGATGCTGCAT	21	11941.8	6464.2	18406	−1.85
nta-miR172d	GGAATCTTGATGATGCTGCAT	21	455.7	234.1	689.8	−1.94
nta-miR172e	TGAATCTTGATGATGCTGCAT	21	8546.5	4098.6	12645.1	−2.08
nta-miR171a	TGATTGAGCCGTGCCAATATC	21	172.8	191.3	364.1	+1.11
nta-miR171b	TGATTGAGCCGCGTCAATATC	21	6.5	27	33.5	+3.73
nta-miR171c	TTGAGCCGCGCCAATATCACT	21	84.2	49.5	133.7	−1.69
nta-miR171d	TGAGCCGGACCAATATCACT	20	47.5	4179.6	4227.1	+86.2
nta-miR171e	CGATGTTGGTGAGGTTCAATC	21	36.7	60.8	97.5	+1.64
nta-miR171f	ATTGATGCGACTCAATCTGAA	21	62.6	13.5	76.1	−4.39
nta-miR166a	TTCGGACCAGGCTTCATTCCC	21	490.3	360.1	850.4	−1.36
nta-miR166b	TCTCGGACCAGGCTTCATTCC	21	993.5	783.3	1776.8	−1.27
nta-miR166c	TTGAGGGGAATGTTGTCTGGC	21	17.3	4.5	21.8	−3.33
nta-miR166d	AATGAAGACTGATCCAAGATC	21	1274.3	1811.9	3086.2	+1.42
nta-miR827	TTAGATGAACATCAACAAACA	21	190.1	85.5	275.6	−2.21
nta-miR2111	TAATCTGCATCCTGAGGTTTA	21	4.3	20.3	24.6	+4.02
nta-miR159	TTTGGATTGAAGGGAGCTCTA	21	1665.2	472.7	2137.9	−3.52
nta-miR164a	TGGAGAAGCAGGGCACGTGCA	21	18816.5	9453.2	28269.7	−1.99
nta-miR164b	CATGTGCCTGTCTTCCCCATC	21	25.9	33.8	59.7	+1.29
nta-miR479	CGTGATATTTGTTTGGCTCATC	22	32.4	195.8	228.2	+5.89
nta-miR477a	ACTCTCCCTCAAGGGCTTCT	20	84.2	141.8	226	+1.68
nta-miR477b	TGTCTCTCCCTCAAGGGCTTC	21	116.6	24.8	141.4	−4.56
nta-miR1444	ACATTCCGGCAATCTTCTCC	20	25.9	13.5	39.4	−1.86
nta-miR319	TGGGAGCCGTAAGATTGAG	19	17.3	13.5	30.8	−1.26
nta-miR1446	TGAACTCTCTCCCTCAATGGCT	22	7170.7	7443.2	14613.9	+1.04
nta-miR395a	CTGAACTCGGTGTAACAAATC	21	8.6	18	26.6	+1.98
nta-miR395b	ATACCTGGCGCTATACATTAA	21	21.6	2.3	23.9	−6.85
nta-miR398	GAATTGTAAGAACATGTAAAA	21	142.5	488.4	630.9	+3.41
nta-miR1384a	AGGAGAATGACAAACCTGACA	21	451.4	630.2	1081.6	+1.4
nta-miR1384b	AGGAGAATCACAAACCTGACA	21	17.3	51.8	69.1	+2.89
nta-miR132a	ATTGTTACATGTAGCACTGGC	21	97.2	49.5	146.7	−1.94
nta-miR132b	ATTGTTACATGTAGCACTGGA	21	157.7	119.3	277	−1.32
nta-miR132c	ATTGTTACATGTAACACTGGC	21	79.9	56.3	136.2	−1.41
nta-miR132d	TATTGTTATATGTTGCACTGGC	22	190.1	128.3	318.4	−1.48
nta-miR3627a	TGTCGCTGGAGAGATGGCACTT	22	1101.5	8658.6	9760.1	+7.85
nta-miR3627b	TCGCAGGAGAGATGGCACTTGC	22	200.9	213.8	414.7	+1.06
nta-miR169a	TGGCAAGCATCTTTGGCGACT	21	47.5	47.3	94.8	+1
nta-miR169b	AACTTGAAGGGTCGTGTA	18	6.5	18	24.5	+2.53
nta-miR4275	ATAAGTGTTCATTGGACAAA	20	34.6	31.5	66.1	−1.1
nta-miR3	GTGTTCATGTTATAATTC	18	6.5	15.8	22.3	+2.24
nta-miR1477	ATGGATAGAAATGAAGGGAGA	21	19.4	11.3	30.7	−1.66
nta-miR399a	GGGCTACTTTCTATTGGCATG	21	15.1	90	105.1	+5.65
nta-miR399b	GGGTAGCTCTCCGTTTGGCAGA	22	79.9	69.8	149.7	−1.14
nta-miR399c	GGGTTACTCTTTATTGGCATG	21	51.8	101.3	153.1	+1.94

ML, Length of mature miRNA, C Reads, Reads of miRNAs before topping, T Reads, Reads of miRNAs after topping.

“+”in fold changes means up-regulation,“−”in fold changes means down-regulation.

**Table 3:** miRNA*s in tobacco roots.

Family	Mature miRNA	ML	C Reads	T Reads	Total Reads	Fold change
nta-miR171*	TGATGTTGGAATGGCTCAATC	21	224.6	506.4	731	+2.25
nta-miR396*	GTTCAAGAAAGCTGTGGGAAA	21	151.2	155.3	306.5	+1.03
nta-miR162*	GGAGGCAGCGGTTCATCGATC	21	54	67.5	121.5	+1.25
nta-miR166a*	GGAATGTTGTCTGGCTCGAGG	21	2533.5	15091.3	17624.8	+5.95
nta-miR393a*	ATCATGCTATCCCTTTGGA	19	71.3	258.8	330.1	+3.59
nta-miR393b*	ATCATGTTATCCCTTTGGA	19	30.2	81	111.2	+2.63
nta-miR393c*	ATCATGCTATCCCTTTGG	18	19.4	227.3	246.7	+11.19
nta-miR172*	GCAGCATCTTCAAGATTCACA	21	49.7	36	85.7	−1.37
nta-miR167d*	AGGTCATCTAGCAGCTTCAAT	21	153.3	337.6	490.9	+2.19

ML, Length of mature miRNA, C Reads, Reads of miRNAs before topping, T Reads, Reads of miRNAs after topping.

“+”in fold changes means up-regulation,“−”in fold changes means down-regulation.

### Identifying new potential miRNAs in tobacco roots

Beside 136 conserved miRNA, we identified a total of 126 new miRNAs candidates in tobacco roots. A homology search (number of mismatch<3) for them in miRBase revealed no known miRNA. Thus, we classified these miRNAs as new tobacco miRNAs and named them as ‘new’ (Table S4). All of the new pre-miRNAs have secondary structures of characteristic stem-loop hairpins (Figure S1) and their alignments with sequenced small RNAs further support the identification of their precursors (Figure 6 and Table S6). The length of the newly identified miRNAs range from 18 to 24 bp in length, and the negative folding free energies vary from −126 to −35 kcal mol^−1^ (with an average of −48.4 kcal mol^−1^) according to MFOLD, which is similar to the free energy values of other plant miRNA precursors. These new miRNAs belong to 77 families (Table S4). Of these 77 families, two major families, nta-miRn2 and nta-miRn26, were identified to contain 11 and 7 members. All other miRNA families contained fewer than five members and most only contained 1 or 2 miRNAs per family.

The levels of some miRNAs, such as nta-miRn1a (4.31 fold), nta-miRn1b (4.43 fold),nta-miRn8 (3.06 fold), nta-miRn13b (5.31 fold), nta-miRn28 (13.77 fold), nta-miRn52 (3.13 fold), nta-miRn57 (14.24 fold), nta-miRn59 (3.13 fold) and nta-miRn66 (9.96 fold), were markedly greater in tobacco roots after topping than that before topping (Figure 7). On the other hand, the levels of several miRNAs, such as nta-miRn11b (4.22 fold), nta-miRn12 (3.26 fold), nta-miRn19 (3.56 fold), nta-miRn60a (6.47 fold) and nta-miRn70 (5.33 fold) were markedly greater in tobacco roots before topping than that after topping (Figure 7).

### Validation of miRNAs in tobacco roots

**Figure 5:**
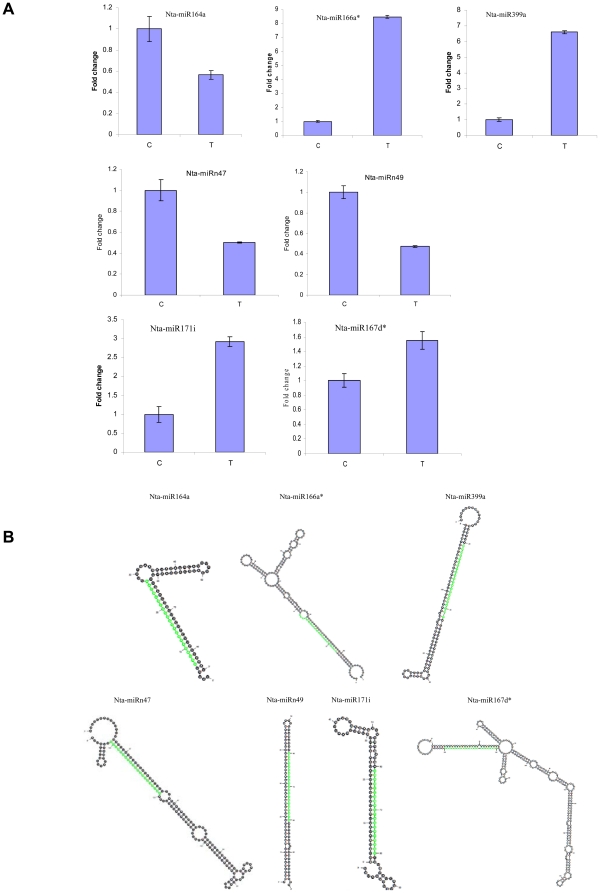
Validation of some miRNAs in tobacco roots. **A**: QPCR products of miRNA. (C, before topping. T, after topping. Error bars indicate one standard deviation of three different biological replicates (n  =  3)). **B**: Predicted hairpin structures of miRNA precursors. (Mature miRNA positions were highlighted in green color.).

**Figure 6:**
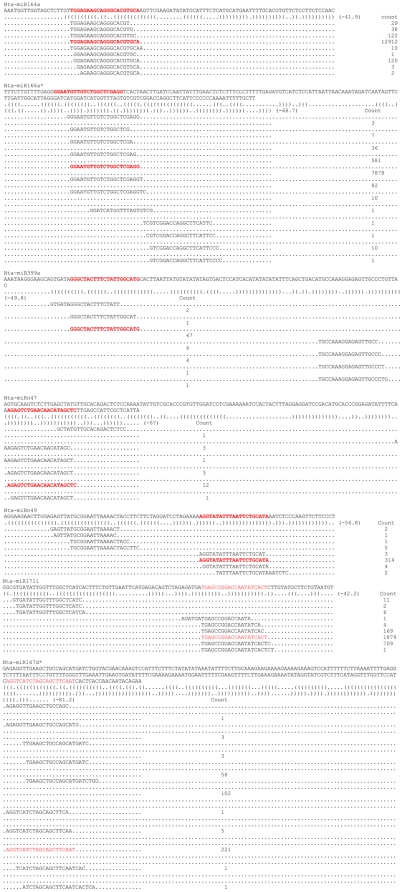
MiRNA precursors and the sequencing reads of miRNA. (Nucleotide bases of mature miRNAs are highlighted with red color.).

To verify the existence and expression change of the identified tobacco miRNAs, the same RNA preparation used in the Solexa sequencing was subjected to quantitative RT-PCR (qRT-PCR) assay. In this study, 5 conserved miRNAs (nta-miR164a, nta-miR166a^*^, nta-miR167d^*^, nta-miR399a and nta-miR171i) and 2 tobacco roots specific miRNAs (nta-miRn47 and nta-miRn49) were validated and measured using qRT-PCR (Figure 5A). As shown in the Figure 5A, the expression changes of these miRNAs before and after topping are similar to the results of Solexa sequencing. These results suggest that miRNAs had been successfully and accurately discovered from tobacco roots with Solexa sequencing. All of these identified miRNAs can be folded into characteristic miRNA stem-loop secondary hairpin structures (some results shown in Figure 5B). Figure 6 shows the sequencing reads patterns and precursors of several miRNAs, and Table S6 shows the sequencing reads patterns and precursors of all new miRNAs, which provide strong evidences to support the identity of these miRNAs in tobacco roots.

**Figure 7:**
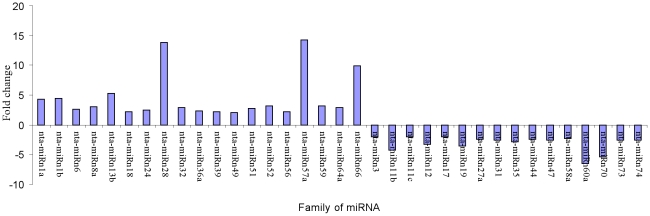
Response of new miRNAs in tobacco roots to topping. (Fold change>2)

### Target prediction of miRNAs in tobacco roots

MiRNAs play an important role in regulating a variety of biological processes. The regulation mechanisms include repression of translation and cleavage of targeted mRNAs. MiRNAs may directly target transcription factors which affect plant development, and also specific genes which control metabolism [26]. To better understand the functions of the identified miRNAs, the potential targets mRNA for tobacco miRNAs were predicted using the psRNATarget program (http://bioinfo3.noble.org/psRNATarget/) with default parameters. Putative targets were identified for 133 out of 136 conserved miRNAs (Table S1) and 126 new miRNAs (Table S2). A few genes with unknown function were predicted as targets for nta-miR4367a, nta-miR390a, nta-miRn1b, nta-miRn8a, nta-miRn17, nta-miRn59, and et al. It is interesting that nta-miRn1b, nta-miRn8a,nta-miRn59 have unknown function, and these miRNAs were markedly induced by topping. No targets can be found in tobacco for nta-miR3, nta-miR319, nta-miR393b^*^, which could be due to limitation of Nicotiana tabacum (tobacco) SGN unigene database. Careful analysis of these potential targets will contribute to our understanding of the role of miRNAs in tobacco roots.

Of the miRNA families whose changes are marked (>2 folds), there are 15 miRNA families involved in tobacco development, 9 miRNA families involved in tobacco N metabolism, 7 miRNA families involved in tobacco response to stress, 3 miRNA families involved in tobacco response to hormone, 1 miRNA families involved in tobacco C metabolism, 10 miRNA families involved in tobacco signal transduction, 3 miRNA families involved in nucleic acid metabolism and 5 miRNA families involved in other metabolisms (Table 4 and Table 5).

**Table 4:** Predicted target functions for up-regulated miRNAs in tobacco roots.

Name of miRNA	Targets	Accession number of targets	Inhibition	Change Fold
(1)Plant development				
Nta-miR171d	scarecrow transcription factor family protein	SGN-U386275	Cleavage	86.2
Nta-miR2111	kelch repeat-containing F-box family protein	SGN-U368810	Cleavage	4.02
Nta-miR160c	myb family transcription factor (MYB48)	SGN-U386631	Cleavage	3.73
Nta-miRn32	aminocyclopropane-1-carboxylate synthase 2/ACC synthase 2	SGN-U387598	Cleavage	2.92
Nta-miRn64	tubulin folding cofactor E	SGN-U368841	Cleavage	2.92
Nta-miR399a	4-coumarate–CoA ligase 2	SGN-U369319	Cleavage	5.65
Nta-miR167*	WRKY family transcription factor	SGN-U363577	Cleavage	2.19
Nta-miRn28	tubulin folding cofactor E	SGN-U368841	Cleavage	13.77
(2)Response to stress				
Nta-miR166a*	ABA-responsive element-binding protein 2(AREB2)	SGN-U374702	Cleavage	5.95
Nta-miRn6	wound-responsive family protein	SGN-U380256	Translation	2.56
Nta-miRn66	zinc finger homeobox family protein	SGN-U377142	Translation	9.96
nta-miRn52	disease resistance family protein	SGN-U383867	Translation	3.13
(3) N metabolism				
Nta-miR398	U2 snRNP auxiliary factor large subunit	SGN-U371373	Cleavage	3.41
Nta-miRn24	branched-chain amino acid transaminase 3 (BCAT3)	SGN-U364225	Cleavage	2.53
Nta-miRn51	methylmalonate-semialdehyde dehydrogenase	SGN-U369274	Cleavage	2.75
Nta-miRn39	protease inhibitor	SGN-U373131	Cleavage	2.22
Nta-miR393b*	elongation factor 1-alpha/EF-1-alpha	SGN-U363242	Cleavage	2.63
(4) Signal transduction				
Nta-miRn18	protein kinase family protein	SGN-U386144	Cleavage	2.22
Nta-miR1384b	protein kinase family protein	SGN-U384902	Cleavage	2.89
Nta-miRn36a	prenylated rab acceptor (PRA1) family protein	SGN-U376290	Cleavage	2.31
Nta-miR171a*	TRAF-type zinc finger-related	SGN-U384164	Cleavage	2.25
Nta-miR3627a	calcium-transporting ATPase	SGN-U371584	Cleavage	7.85
Nta-miR169b	calmodulin-related protein	SGN-U383482	Cleavage	2.53
(5) Nucleic acid metabolism				
Nta-miR479	SWIB complex BAF60b domain-containing protein	SGN-U376283	Translation	5.89
Nta-miR160a	PAZ domain-containing protein/piwi domain-containing protein	SGN-U364054	Cleavage	3.08
Nta-miR160b	PAZ domain-containing protein/piwi domain-containing protein	SGN-U364054	Cleavage	2.13
Nta-miRn1a	TFIIH basal transcription factor complex p34 subunit	SGN-U386915	Cleavage	4.31
(6) Other metabolisms				
Nta-miR171b	transporter protein	SGN-U372710	Cleavage	3.73
Nta-miRn56	sterol desaturase family protein	SGN-U372201	Cleavage	2.24

**Table 5:** Predicted target functions for down-regulated miRNAs in tobacco roots.

Name of miRNA	Targets	Accession number of targets	Inhibition	Change Fold
(1)Plant development				
Nta-miR156a	squamosa promoter-binding protein-like 9 (SPL9)	SGN-U384021	Cleavage	4.16
Nta-miR156e	squamosa promoter-binding protein-like 9 (SPL9)	SGN-U384021	Cleavage	2.34
Nta-miR156f	squamosa promoter-binding protein-like 4 (SPL4)	SGN-U367182	Cleavage	23.8
Nta-miR156b	squamosa promoter-binding protein-like 9 (SPL9)	SGN-U384021	Cleavage	2.47
Nta-miR156c	squamosa promoter-binding protein-like 9 (SPL9)	SGN-U384021	Cleavage	3.30
Nta-miR156d	squamosa promoter-binding protein-like 9 (SPL9)	SGN-U384021	Cleavage	2.46
Nta-miR159	myb family transcription factor	SGN-U373553	Cleavage	3.52
Nta-miR827	WRKY family transcription factor	SGN-U369920	Cleavage	2.21
Nta-miR172e	floral homeotic protein APETALA2 (AP2)	SGN-U378175	Cleavage	2.08
Nta-miRn47	protein tyrosine phosphatase-like protein	SGN-U375304	Translation	2.62
Nta-miRn60a	autophagocytosis-associated family protein	SGN-U379415	Cleavage	6.47
Nta-miRn31	zinc finger (C2H2 type) family protein	SGN-U372557	Cleavage	2.50
(2)Response to stress				
Nta-miRn11b	disease resistance protein (CC-NBS class)	SGN-U375884	Cleavage	4.22
Nta-miRn11c	calcium-binding EF hand family protein	SGN-U364357	Cleavage	2.02
Nta-miRn35	phytochelatin synthetase	SGN-U383096	Cleavage	2.91
Nta-miRn70	UDP-glucosyl transferase family protein	SGN-U362891	Translation	5.33
nta-miR166c	leucine-rich repeat family protein	SGN-U372178	Translation	3.33
(3)Response to hormone				
Nta-miR395b	dehydration-responsive protein (RD22)	SGN-U370839	Translation	6.85
Nta-miRn49	axi 1	SGN-U378766	Translation	2.00
Nta-miR164a	transcription activator NAC1 (NAC1)	SGN-U387801	Cleavage	2.00
(4) N metabolism				
Nta-miRn73	nitrate transporter	SGN-U384171	Cleavage	2.54
Nta-miR394a	AAA-type ATPase family protein	SGN-U365301	Cleavage	2.72
Nta-miRn19	glutamine-tRNA ligase	SGN-U387453	Cleavage	3.56
Nta-miRn27a	ubiquitin family protein	SGN-U367623	Cleavage	2.45
(5) C metabolism				
Nta-miRn3	cellulose synthase	SGN-U368912	Cleavage	2.06
(6) Signal transduction				
Nta-miRn58a	mitogen-activated protein kinase kinase (MAPKK)	SGN-U363248	Cleavage	2.28
Nta-miRn74	ATPase, plasma membrane-type	SGN-U371340	Translation	2.54
Nta-miRn44	protein kinase (ATN1)	SGN-U372350	Cleavage	2.46
Nta-miR477b	protein kinase family protein	SGN-U375246	Cleavage	4.56
(7) Other metabolisms				
Nta-miRn12	proteasome maturation factor UMP1 family protein	SGN-U365900	Cleavage	3.26
Nta-miR397b	laccase	SGN-U374824	Cleavage	3.34
Nta-miR4367b	flavin reductase	SGN-U364353	Cleavage	2.12

## Discussion

MiRNAs provide global regulations both through posttranscriptional and translational regulation and chromatin modification. Identification of entire set of miRNAs and their targets will lay the foundation to unravel the complex miRNAs mediated regulatory networks controlling development and other physiological processes. Recently, a large number of miRNA have been found in various species. For example, the identified number of miRNAs in Arabidopsis, rice, maize and wheat were 232, 491, 170 and 58, respectively (miRBase13.0, http://www.mirbase.org). But most species-specific miRNAs are still unidentified and much fewer miRNAs from tobacco have been identified. In present study, our Solexa high-throughput sequencing of tobacco roots small RNAs revealed a diverse and complex small RNA population, and expression of the 136 conserved miRNAs and 126 new miRNAs were determined. Therefore, the miRNAs sequenced in this study can definitely provide the information of tobacco miRNAs for further study on their gene regulation function in response to topping.

To assess and define a putative function for a miRNA in plant, a further step of target identification is necessary. Currently, the most efficient tool available for this is the bioinformatics approach facilitated by the high degree of homology between miRNA and its target sequences in plants [27]. In this study, putative targets were identified for 133 out of 136 conserved miRNAs and 126 new miRNAs. Although targets can be predicted for many new miRNAs, the rate of false positives is usually higher for new miRNAs than for conserved miRNAs [28]. Therefore, the targets of new miRNAs need to be further validated. Of these miRNAs whose targets had been identified, the miRNAs which change markedly (>2 folds) belong to 53 families and their targets have different biological functions including plant development, response to stress, response to hormone, N metabolism, C metabolism, signal transduction, nucleic acid metabolism and other metabolism.

### 1. Plant development

15 miRNA families may be involved in tobacco development, which is consistent with tobacco transition from reproductive to vegetative phase induced by topping. Of these families, nta-miR156f was only expressed in tobacco roots before topping. Squamosa promoter-binding protein-like (SPL) is the target of nta-miR156 family. SPL genes encode plant-specific transcription factors that play important roles in plant phase transition, plant architecture and gibberellins signaling [29]. Autophagocytosis-associated family protein is the target of nta-miRn60a, and it reduces shoot anthocyanin accumulation in response to cytokinin feeding to the roots, having a role in cytokinin regulated root-shoot communication. Leaf senescence can also be accelerated by the disruption of an Arabidopsis autophagy gene [30]. In the study, the expression of nta-miR156 family and nta-miRn60a were significantly repressed by topping. Scarecrow (SCR) is a member of the plant-specific GRAS family and plays a significant role in the radial patterning of both roots and shoots [31]. Nta-miR171d targets the scarecrow transcription factor family protein. In the study, nta-miR171d was markedly induced by topping. Therefore topping can affect the radial patterning of tobacco roots. Since nta-miRn60a and nta-miR171d are involved in root development, it is easy to understand the increase in the activity, number and biomass of the roots after topping. Nta-miR160c targets myb48 which regulates the secondary growth [32]. Nta-miR399a targets 4-Coumarate-coenzyme A ligase which functions early in the general phenylpropanoid pathway by producing the monolignol precursor p-coumaroyl-CoA. This metabolite is also a precursor for the production of secondary metabolites such as stilbenes and flavonoids [33]. Therefore, the secondary metabolism in tobacco roots can be regulated by miRNAs to respond to topping.

The differential miRNAs were also found to be involved in other tobacco development metabolism after topping. Nta-miRn31 targets zinc finger (C2H2 type) family protein which has an important role in plant development including floral organogenesis, leaf initiation, lateral shoot initiation, gametogenesis and seed development [34]. Nta-miRn47 targets tyrosine phosphatase-like protein which is associated with cell de-differentiation and proliferation [35]. Floral homeotic protein APETALA2 (AP2) is the target of nta-miRn172e. AP2, a transcription factor known to act in floral patterning and seed development, regulates stem cell maintenance in the SAM through the CLV–WUS pathway. It had been found to have a recruitment of miR172 in the building of a flower in evolution [36]. In this study, nta-miRn49, nta-miRn31 and nta-miR172e were markedly repressed by topping. Nta-miRn32 targets aminocyclopropane-1-carboxylate synthase 2 (ACS2) which has a role in the regulation of plant maturing [37]. Nta-miRn64 and nta-miRn28 target tubulin folding cofactor E which is necessary for continuous microtubule organisation, mitotic division and cytokinesis [38]. Kelch repeat-containing F-box family is target of nta-miR2111, and it affects the circadian clock and flowering time [39]. In the study, nta-miR2111, nta-miRn32, nta-miRn64 and nta-miRn28 were significantly induced by topping.

### 2. Response to stress

7 miRNA families may be involved in tobacco response to stress. Nta-miRn70 targets UDP-glucosyl transferase family protein. Glycosyltransferases (GTs) plays important roles in stress responses of plants by glycosylating hormones and secondary metabolites [40]. Nta-miRn11b targets disease resistance protein (CC-NBS class), and nta-miRn11c targets calcium-binding EF hand family protein which is part of a cellular response to oxidative stress [41]. Nta-miR166c targets leucine-rich repeat family protein which has functions in disease resistance [42]. Nta-miRn35 targets phytochelatin synthetase which is induced by heavy metals [43]. In this study, nta-miRn11b, nta-miRn11c, nta-miRn166c, nta-miRn35 and nta-miRn70 were significantly repressed by topping. Nta-miR166a^*^ targets AREB2 which is a transcription factors that regulates ABRE-dependent gene expression for ABA signaling under conditions of water stress [44]. Nta-miRn66 targets zinc finger homeobox family protein which has a role in the plant defense-signaling pathway [45]. Nta-miRn52 targets disease resistance family protein. In this study, nta-miR166a*, nta-miRn66 and nta-miRn52 were markedly induced by topping. Since topping is considered as wounding stress [2], it is easy to understand the changes in expression of miRNAs involved in response to stress.

### 3. N metabolism

9 miRNA families may be involved in N metabolism. Nta-miRn73 targets nitrate transporter. Inorganic nitrogen is a vital nutrient for plants. Plants take up and assimilate both nitrate and ammonium with nitrate being the predominant form in most agricultural soils. Nitrate is taken up by roots then transported into cells via transporters from the NRT1 and NRT2 family of nitrate transporters [46]. In the study, nta-miRn73 was markedly repressed by topping, which means that topping can promote roots to take up nitrate, and the result is consistent with the previous report [47]. Nta-miRn51 targets methylmalonate-semialdehyde dehydrogenase (MMSDH) which participates in 3 metabolic pathways: inositol metabolism, valine, leucine and isoleucine degradation, and propanoate metabolism. It has been proved that MMSDH may play a role in root development and leaf sheath elongation in rice [48]. In this study, nta-miRn51 was markedly induced by topping.

Nta-miR394a targets AAA-type ATPase family protein which plays an important role in protein unfoldase activity, including the dissociation of protein complexes [49]. Glutamine-tRNA ligase is the target of nta-miRn19, which is related to amino acid metabolism. Nta-miRn27a targets ubiquitin family protein which participates in the degradation of protein. In this study, nta-miRn19, nta-miRn27a and nta-miR394a were significantly repressed by topping. Nta-miR398 targets U2 snRNP auxiliary factor (U2AF) large subunit. The poly(A)-limiting element (PLE) restricts the length of the poly(A) tail to <20 nt when present in the terminal exon of a pre-mRNA. U2AF may have a role in PLE regulation of poly(A) tail length [50]. Nta-miRn39 targets protease inhibitor which inhibits the degradation of proteins. Elongation factor 1-alpha (EF-1-alpha) is the target of miRn393b^*^. EF-1-alpha participates in protein biosynthesis. Branched-chain amino acid transaminase 3 (BCAT3) is the target of nta-miRn24. BCAT3 has the dual role in amino acid and glucosinolate biosynthesis [51]. In this study, nta-miR398, nta-miRn39, nta-miRn24 and nta-miRn393b^*^ were markedly induced by topping.

### 4. C metabolism

1 miRNA families may be involved in C metabolism. Nta-miRn3 targets cellulose synthase which is required for cell wall integrity during root formation [52]. In the study, nta-miRn3 was markedly repressed by topping.

### 5. Response to hormone

3 miRNA families may be involved in tobacco response to hormone. Nta-miR395b targets dehydration-responsive protein (RD22) which is induced by ABA [53]. Nta-miRn49 targets axi 1 which plays a role in auxin action [54]. Nta-miR164a targets transcription activator NAC1 (NAC1) which mediates auxin signaling to promote lateral root development [55]. In this study, nta-miR395b, nta-miR164a and nta-miRn49 were markedly repressed by topping. Since topping can induce some complex changes of phytohormones in tobacco [1], it is easy to understand the changes in expression of miRNAs involved in response to hormone.

### 6. Signal transduction

10 miRNA families may be involved in signal transduction in tobacco. Nta-miRn58a targets mitogen-activated protein kinase kinase (MAPKK). Sequential activation of kinases within MAPKK cascades is a common and evolutionary-conserved mechanism of signal transduction [56]. Nta-miRn74 targets plasma membrane-type ATPase which participates in signaling pathways. Nta-miR477b and nta-miRn44 target protein kinase family protein. These protein kinases are involved in signal transduction. In this study, nta-miRn74, nta-miRn58a, nNta-miR477b and nta-miRn44 were markedly repressed by topping. Nta-miRn36a targets prenylated rab acceptor (PRA1) family protein which has a function in both secretory and endocytic intracellular trafficking pathways [57]. Nta-miR3627a targets calcium-transporting ATPase which has an important role in Ca^2+^-dependent signaling pathways. Calmodulin-related protein is the target of nta-miR169b, and it plays a role in the signal transduction pathways activated by the inductive stimuli [58]. Nta-miR171a^*^ targets TRAF-type zinc finger-related protein which is a signal mediator of cell surface receptors [59]. Nta-miRn18 and nta-miRn1384b target protein kinase family protein which has a role in the signal transduction pathways. In this study, nta-miRn18, nta-miRn1384b, nta-miRn36a, nta-miR3627a, nta-miR169b and nta-miR171a^*^ were markedly induced by topping. Therefore, there is a complex signal transduction in response of tobacco to topping.

### 7. Nucleic acid metabolism

3 miRNA families may be involved in tobacco RNA metabolism. Nta-miR160a and nta-miR160b target polyadenylate-binding protein which plays roles in mRNA stability and translation [60]. SWIB complex BAF60b domain-containing protein is the target of nta-miR479. The SWI/SNF chromatin remodelling complexes are important regulators of transcription, and BAF60b bridges interactions between transcription factors and SWI/SNF complexes [61]. Nta-miRn1a targets TFIIH basal transcription factor which catalyzes Phosphorylation of RNA polymerase II [62]. In this study, nta-miR479, nta-miRn1a, nta-miR160a and nta-miR160b were markedly induced by topping. Therefore, transcriptional regulation is an important regulation in response of tobacco to topping.

### 8. Other metabolisms

There are 5 miRNA families involved in other metabolisms. Nta-miRn56 targets sterol desaturase family protein which catalyzes the biosynthesis of unsaturated sterols [63]. In this study, nta-miRn56 was markedly induced by topping, therefore topping may also change sterols metabolism in tobacco roots. Nta-miRn12 targets proteasome maturation factor UMP1 family protein. Ump1 is responsible for maturation of the catalytic core of the 26S proteasome [64]. Nta-miR397b targets laccase which are often able to catalyze oxidation of a broad range of substrates, such as phenols and amines in vitro, their precise physiological/biochemical roles in higher plants remain largely unclear [65]. Nta-miR4367b targets flavin reductase which catalyzes electron transfer processes. In this study, nta-miR4367b, nta-miRn12 and nta-miR397b were markedly repressed by topping.

## Supporting Information

Figure S1 **Secondary structures of new pre-miRNAs.**
(PDF)

Table S1 **Details of conserved miRNAs from tobacco roots.** ML, Length of mature miRNA, C Reads, Reads of miRNAs before topping, T Reads, Reads of miRNAs after topping. “+”in fold changes means up-regulation,“−”in fold changes means down-regulation.
(XLS)

Table S2 **Details of new miRNAs from tobacco roots.** ML, Length of mature miRNA, C Reads, Reads of miRNAs before topping, T Reads, Reads of miRNAs after topping. “+”in fold changes means up-regulation,“−”in fold changes means down-regulation.
(XLS)

Table S3 Details of miRNA* from tobacco roots. ML, Length of mature miRNA, C Reads, Reads of miRNAs before topping, T Reads, Reads of miRNAs after topping. “+”in fold changes means up-regulation,“−”in fold changes means down-regulation.
(XLS)

Table S4 **New miRNAs in tobacco roots.** ML, Length of mature miRNA, C Reads, Reads of miRNAs before topping, T Reads, Reads of miRNAs after topping. “+”in fold changes means up-regulation,“−”in fold changes means down-regulation.
(XLS)

Table S5 **The primer of miRNAs for qPCR.**
(XLS)

Table S6 **The sequencing reads patterns and precursors of all new miRNAs.**
(XLS)
