# Supplementary material for: Differential Expression of miRNAs in Response to Topping in Flue-Cured Tobacco (Nicotiana tabacum) Roots
Source: PLoS One. 2011 Dec 14;6(12):e28565. doi: 10.1371/journal.pone.0028565 (PMC3237444; doi:10.1371/journal.pone.0028565)
Supplement: Figure S1 — Secondary structures of new pre-miRNAs. (PDF) [file pone.0028565.s001.pdf]

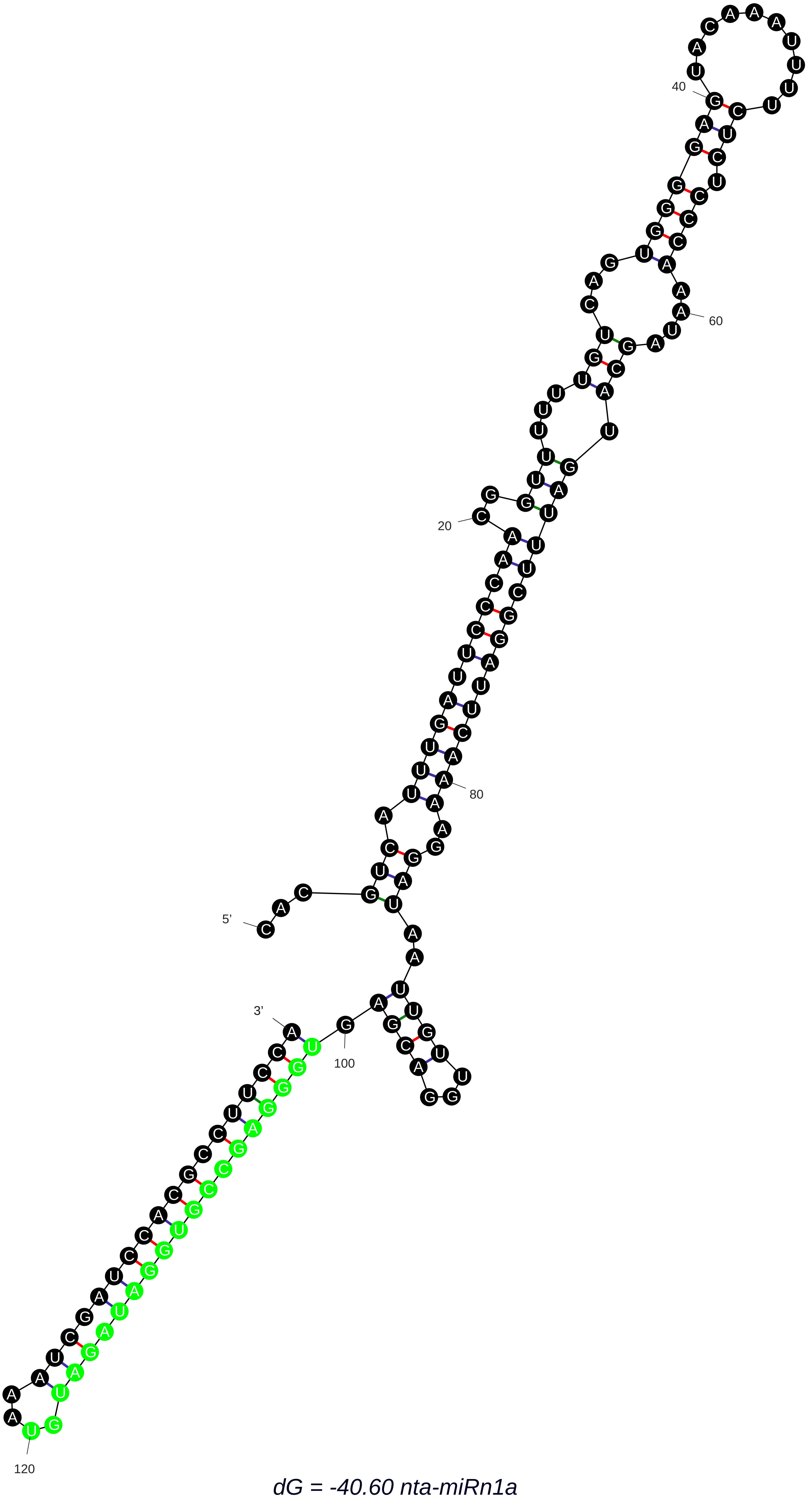

dG = -40.60 nta-miRn1a

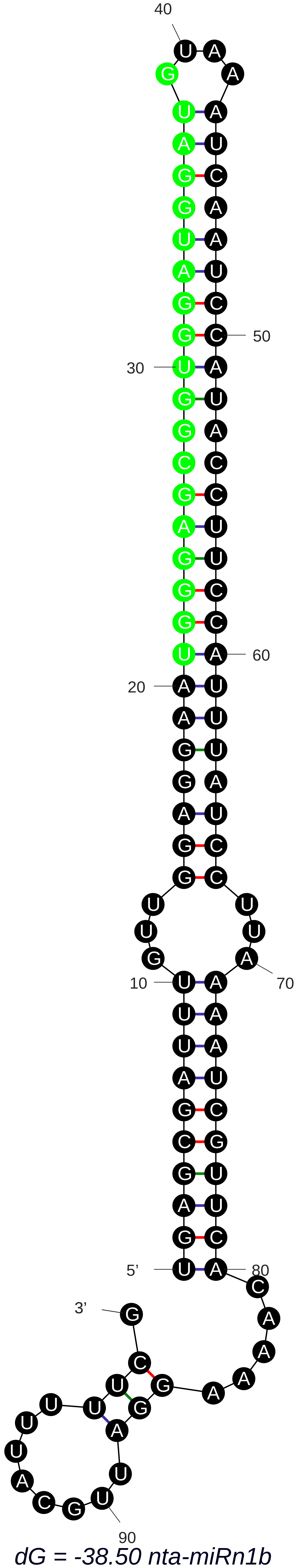

dG = -38.50 nta-miRn1b

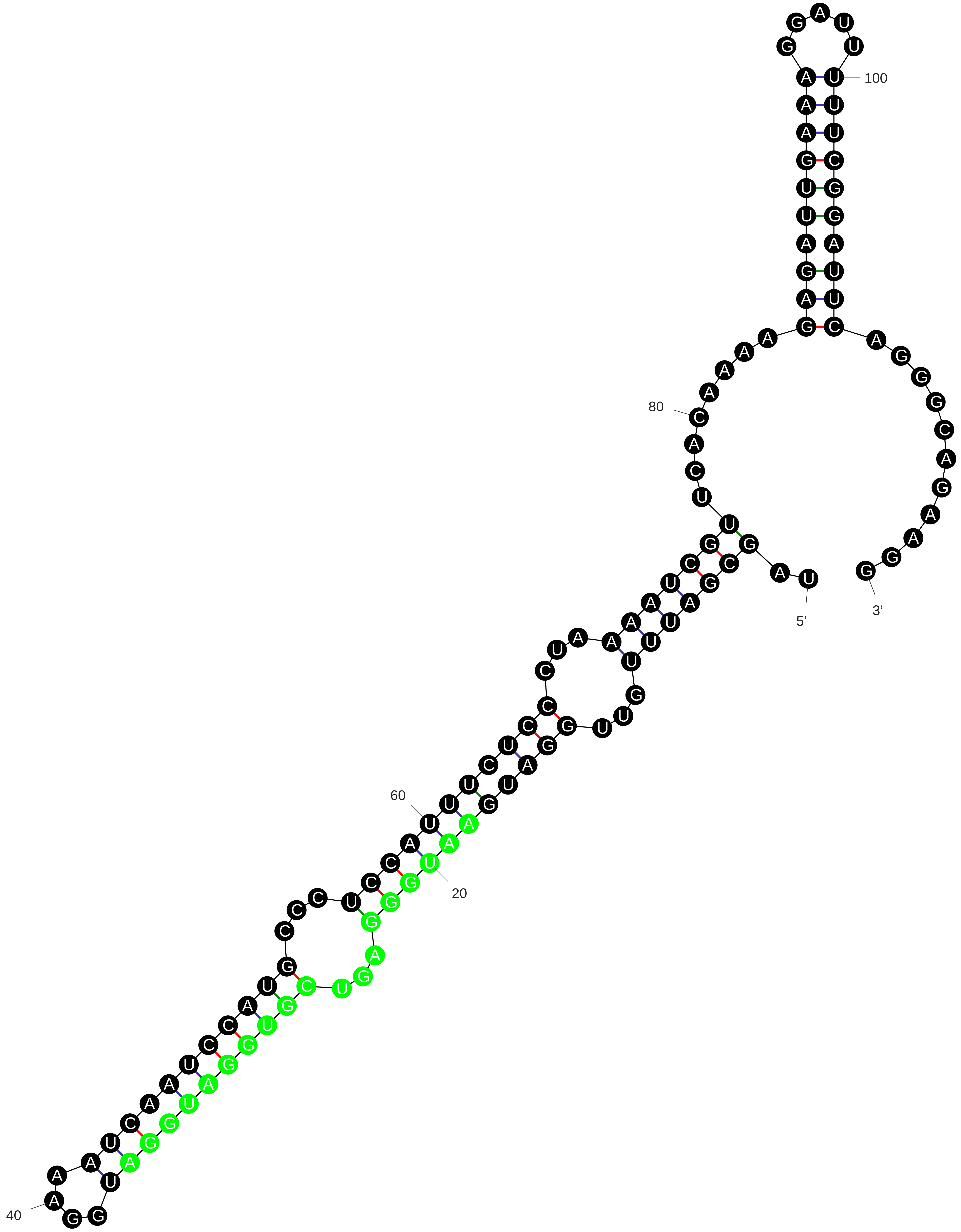

dG = -36.30 nta-miRn1c

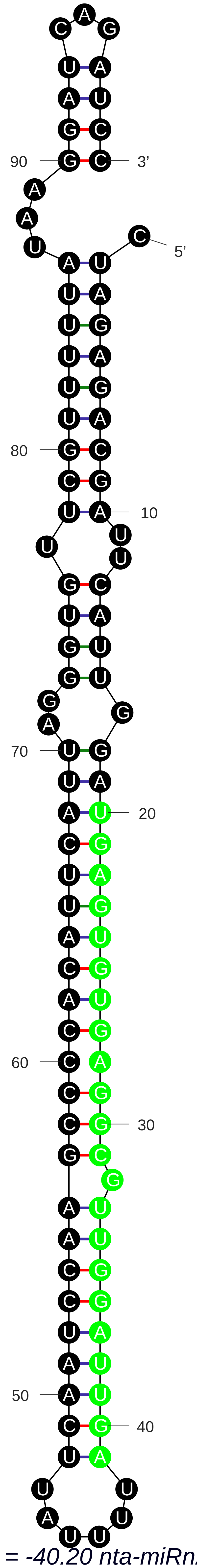

dG = -40.20 nta-miRn2a

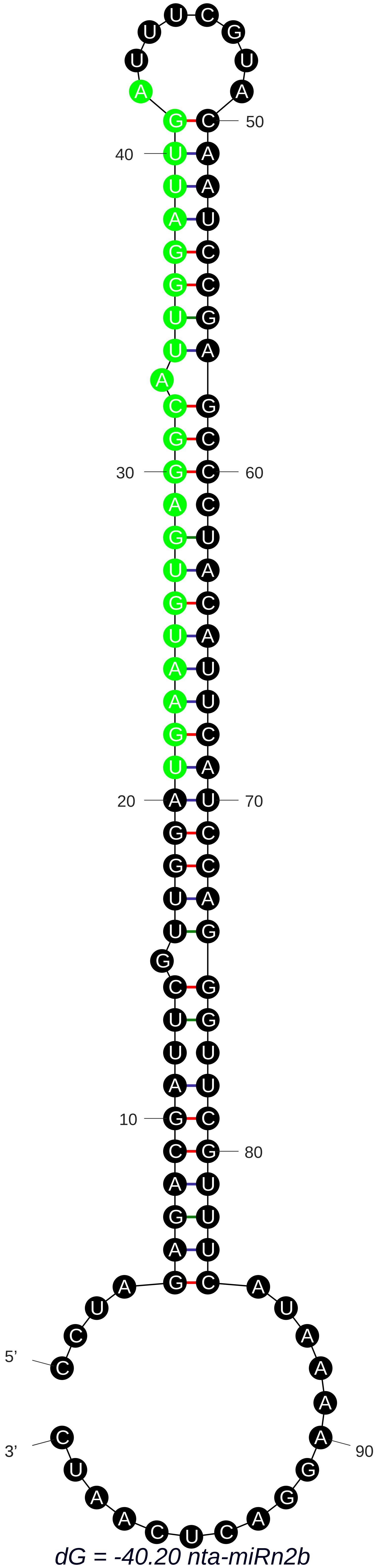

dG = -40.20 nta-miRn2b

Output of sir_graph (©)
mfold_util 4.6

Created Mon Oct 17 22:33:20 2011

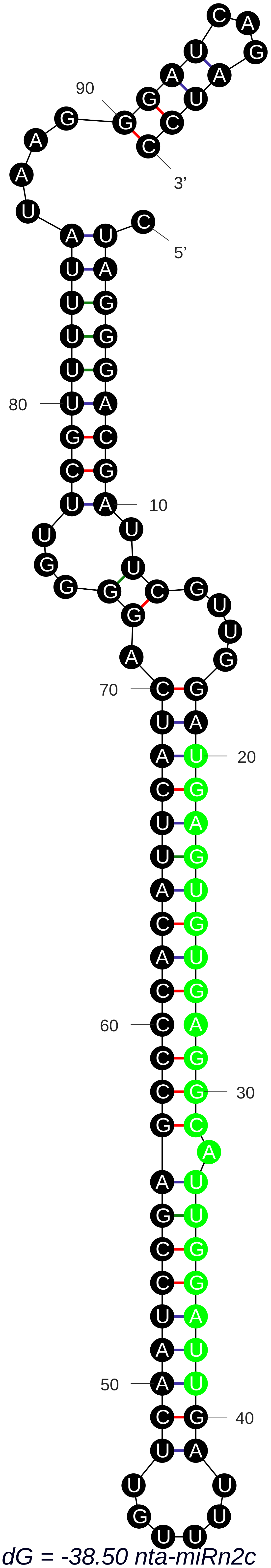

dG = -38.50 nta-miRn2c

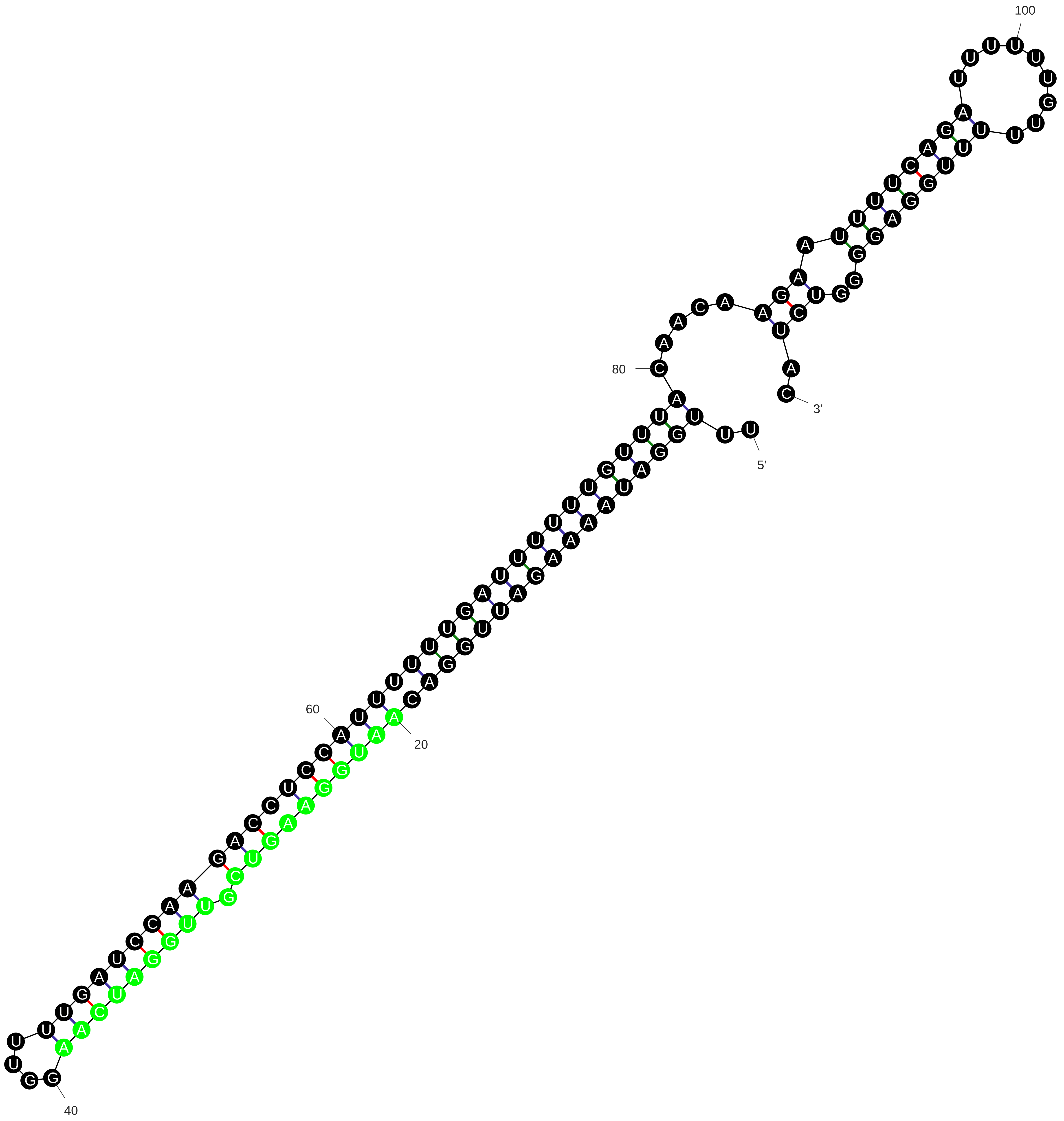

dG = -39.00 nta-miRn3

Output of sir_graph (©)
mfold_util 4.6

Created Mon Oct 17 22:38:24 2011

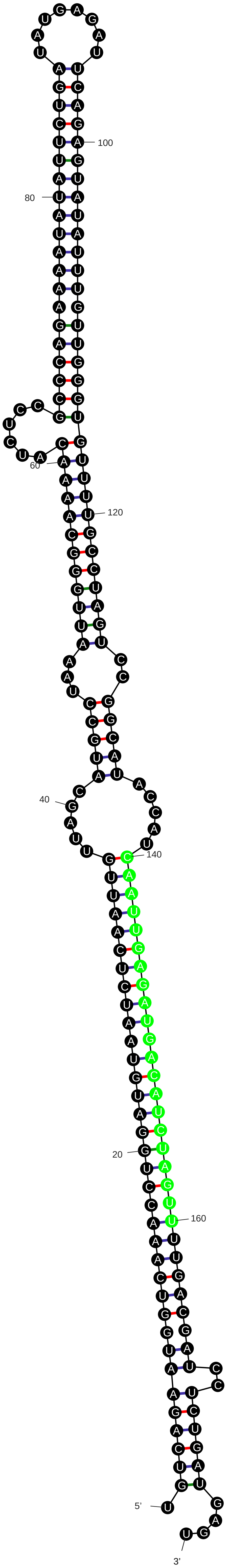

dG = -74.60 nta-miRn4

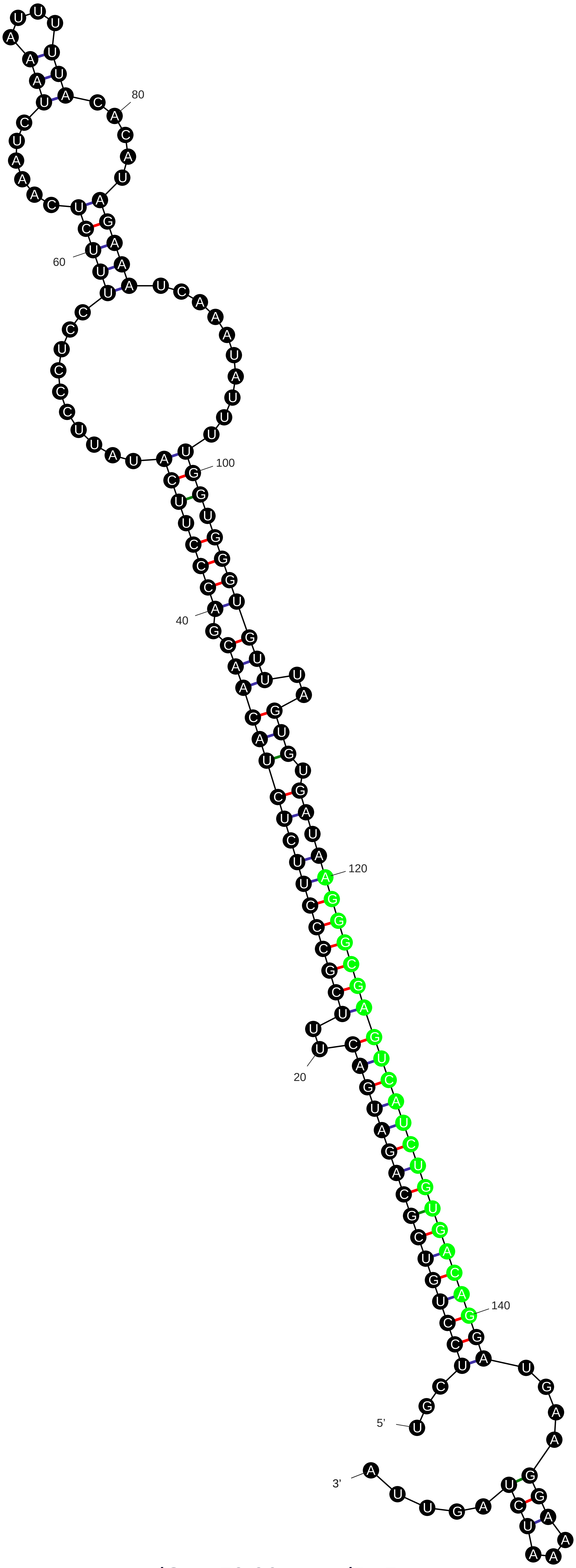

dG = -52.20 nta-miRn5a

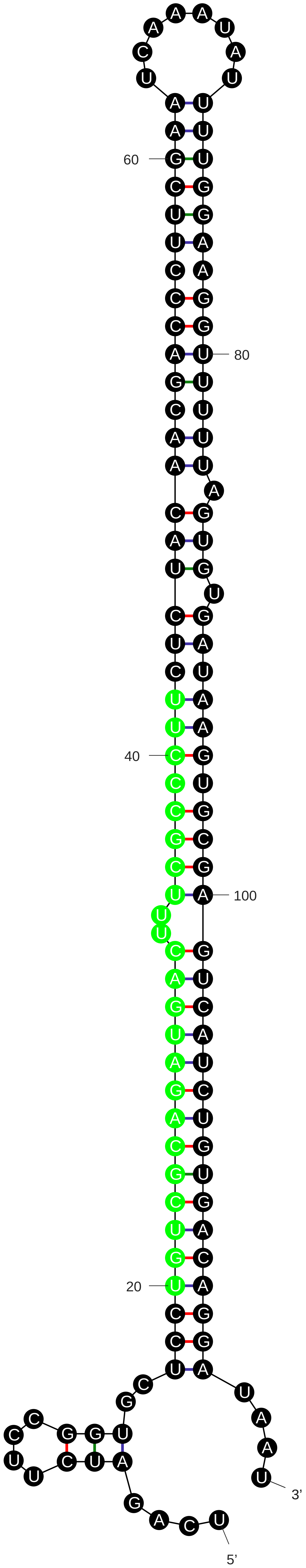

dG = -43.60 nta-miRn5b

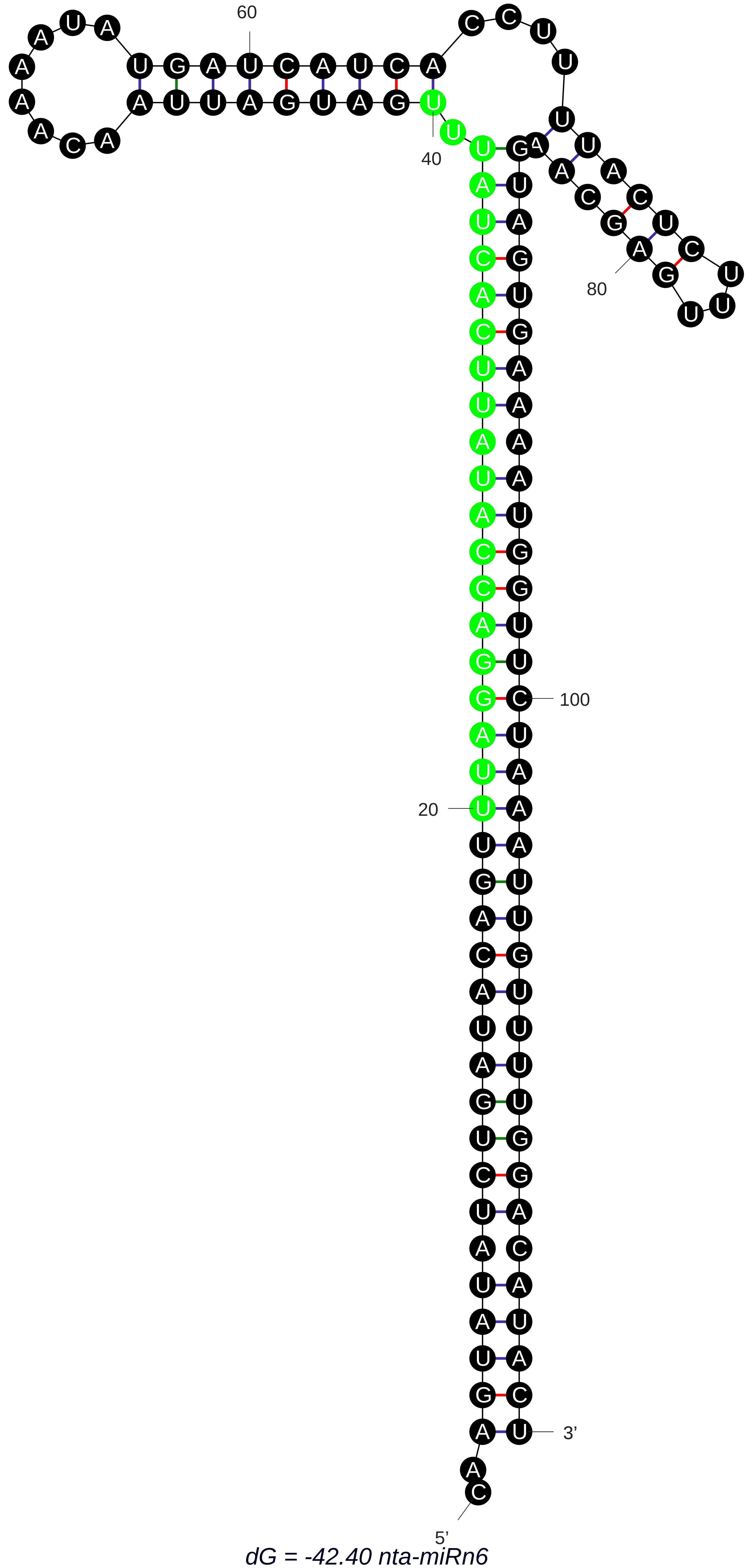

dG = -42.40 nta-miRn6

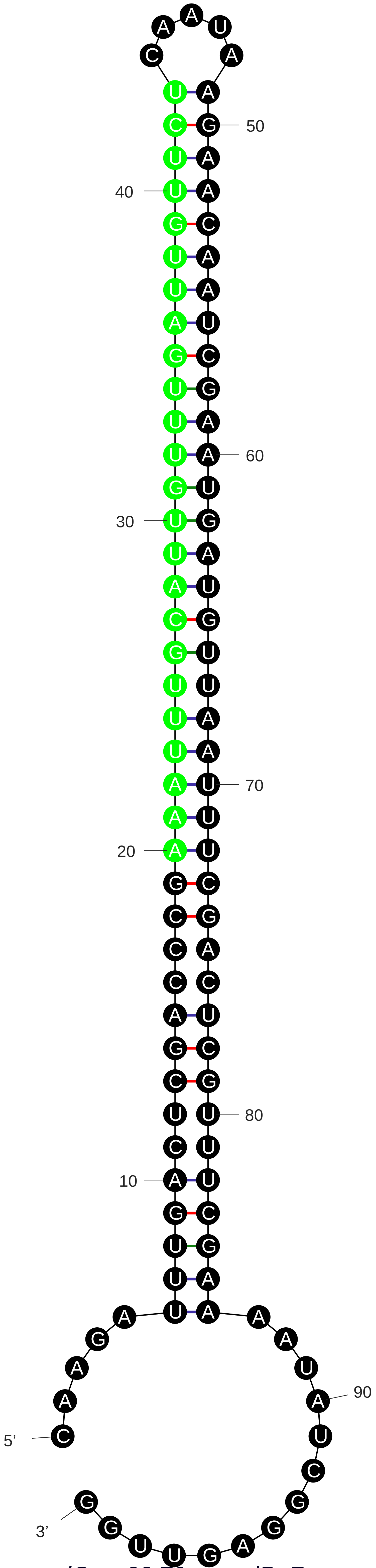

dG = -33.70 nta-miRn7

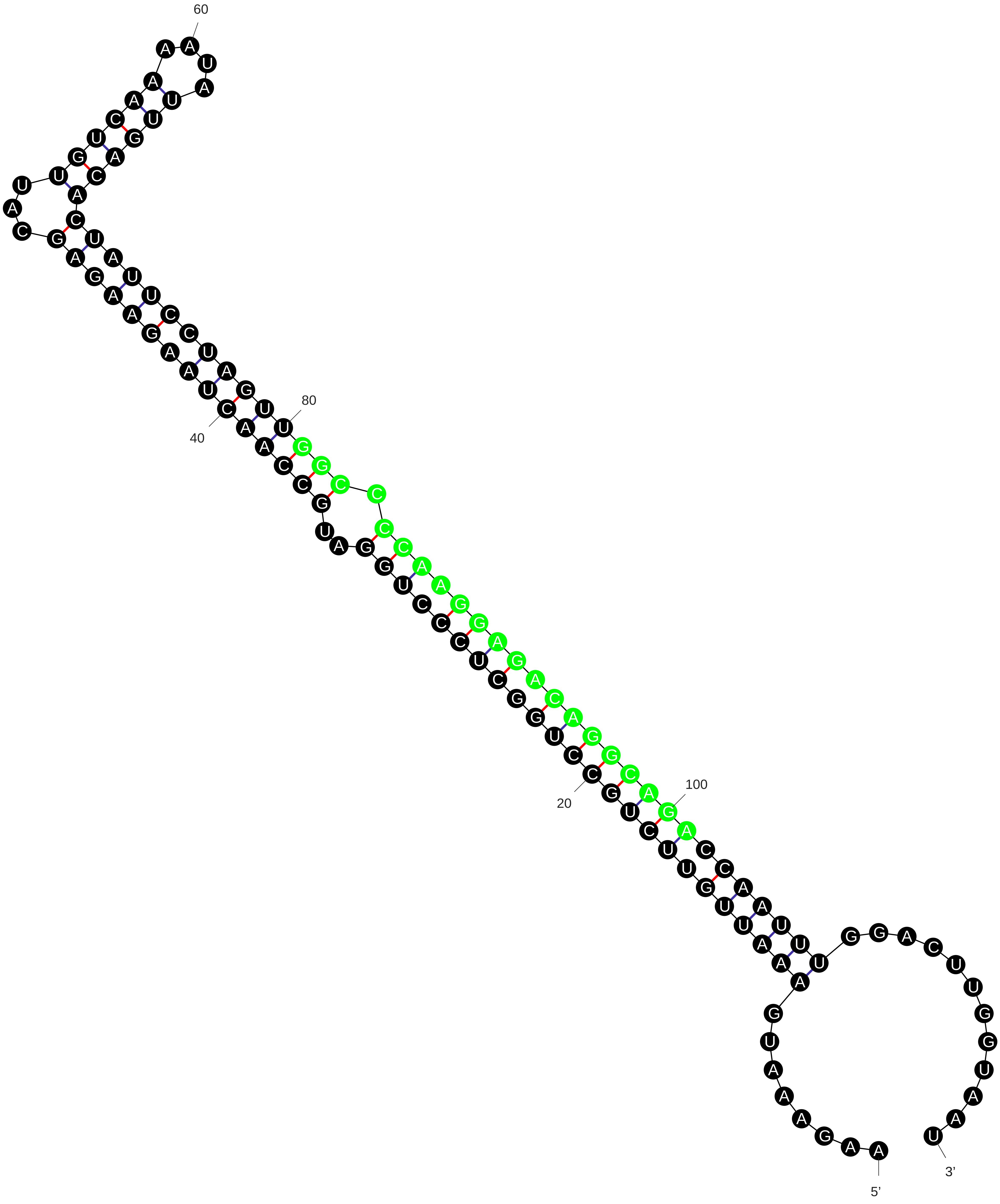

dG = -47.40 nta-miRn8

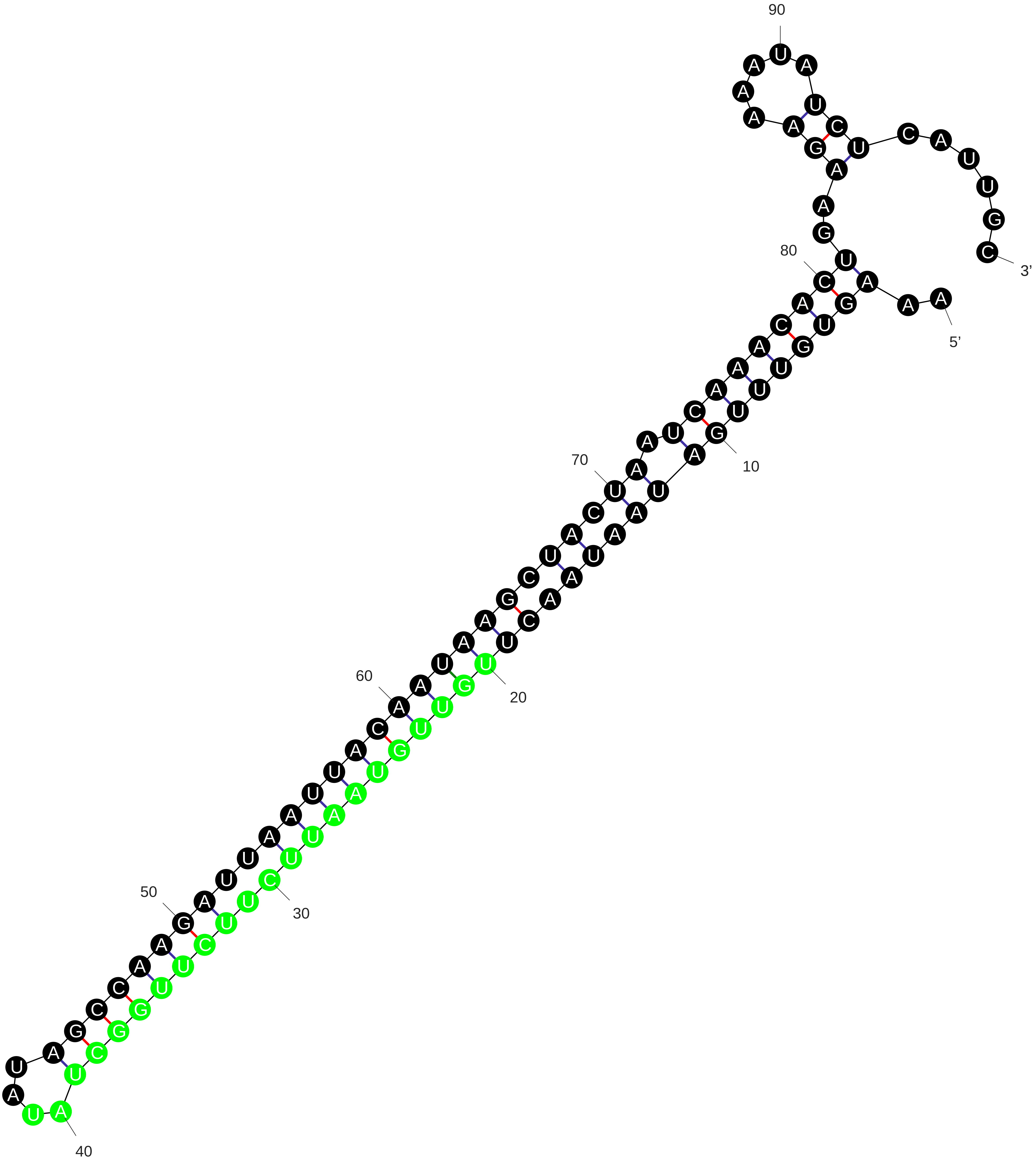

dG = -34.80 nta-miRn9

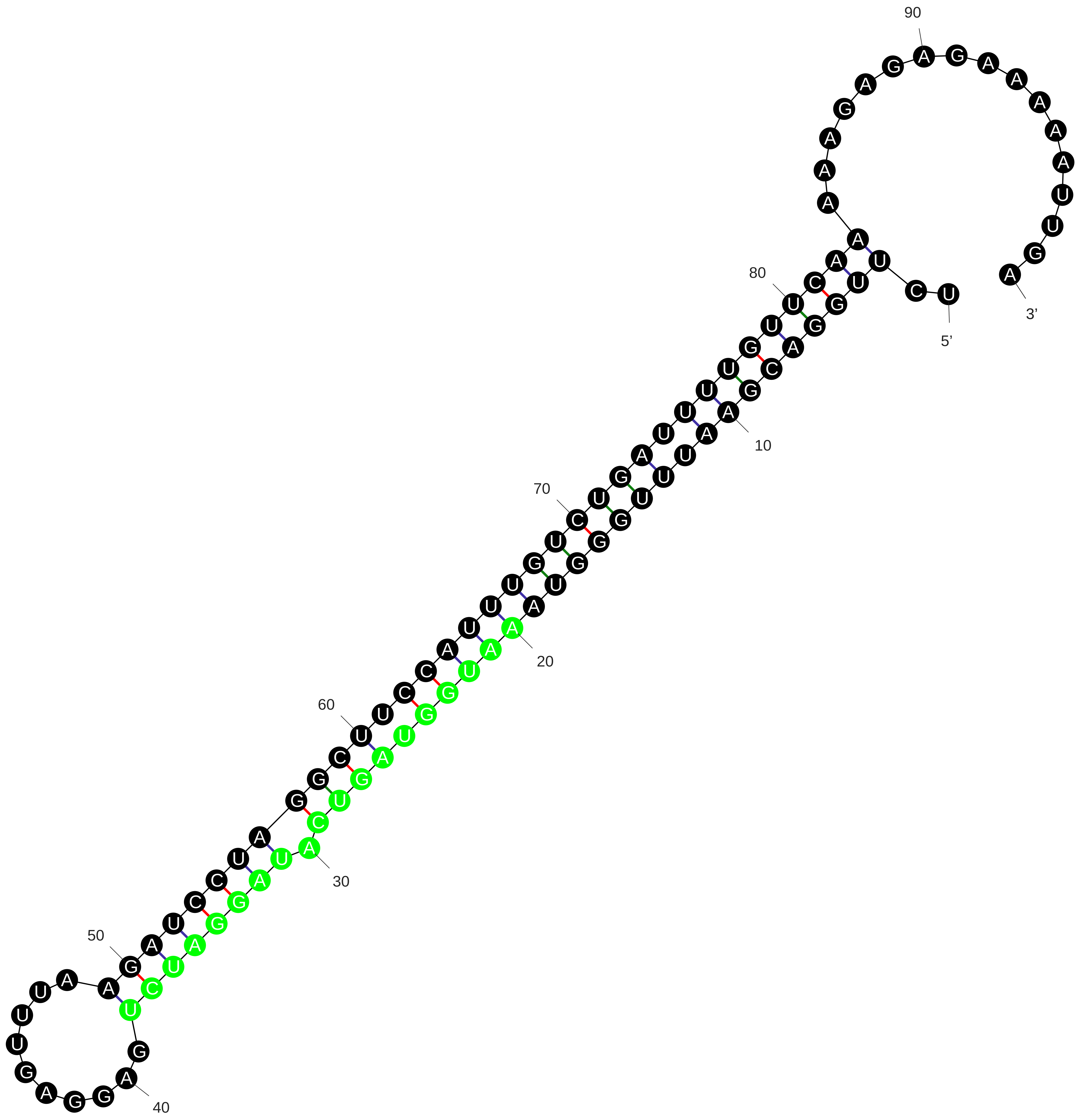

dG = -33.50 nta-miRn10

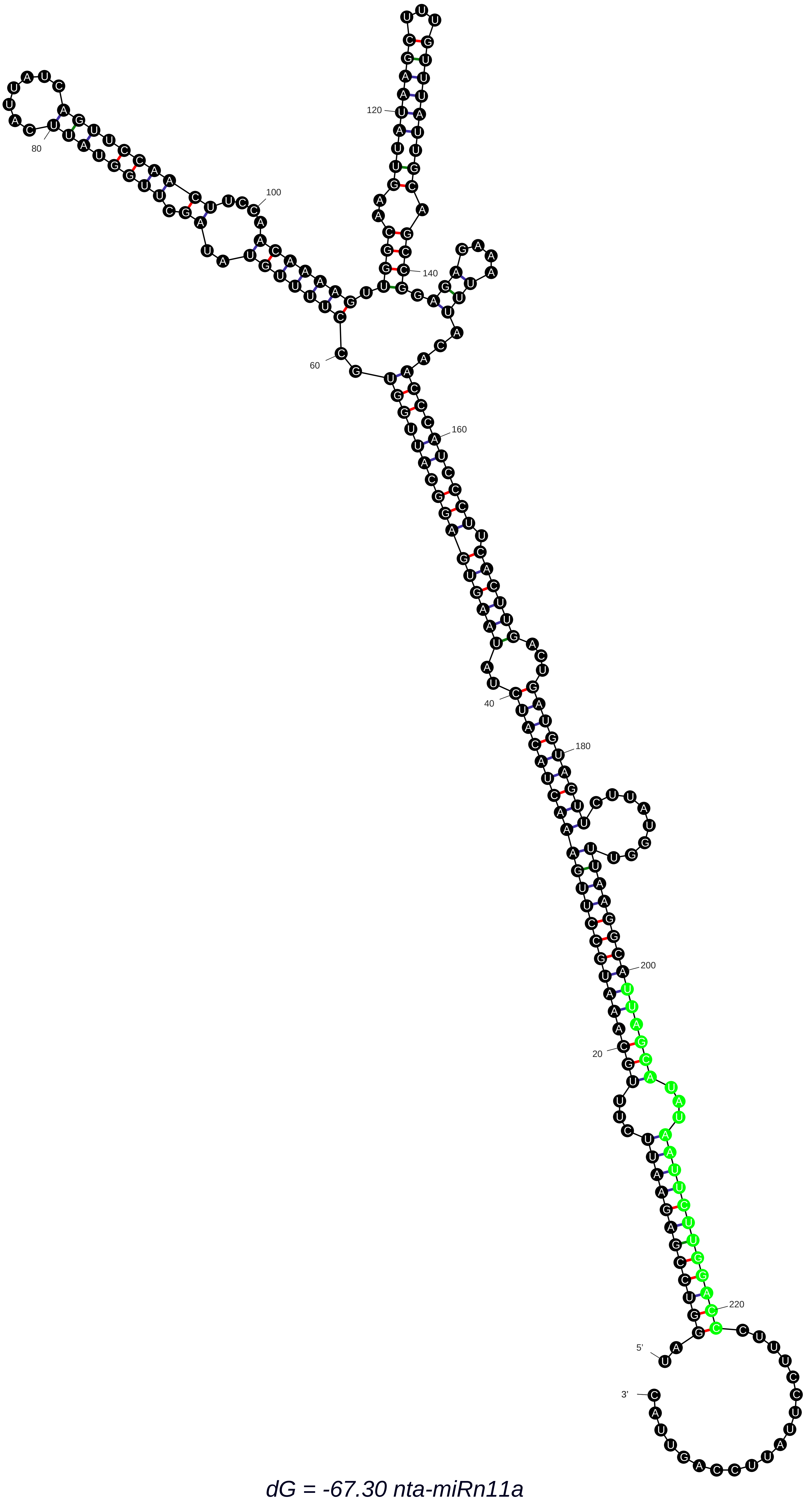

dG = -67.30 nta-miRn11a

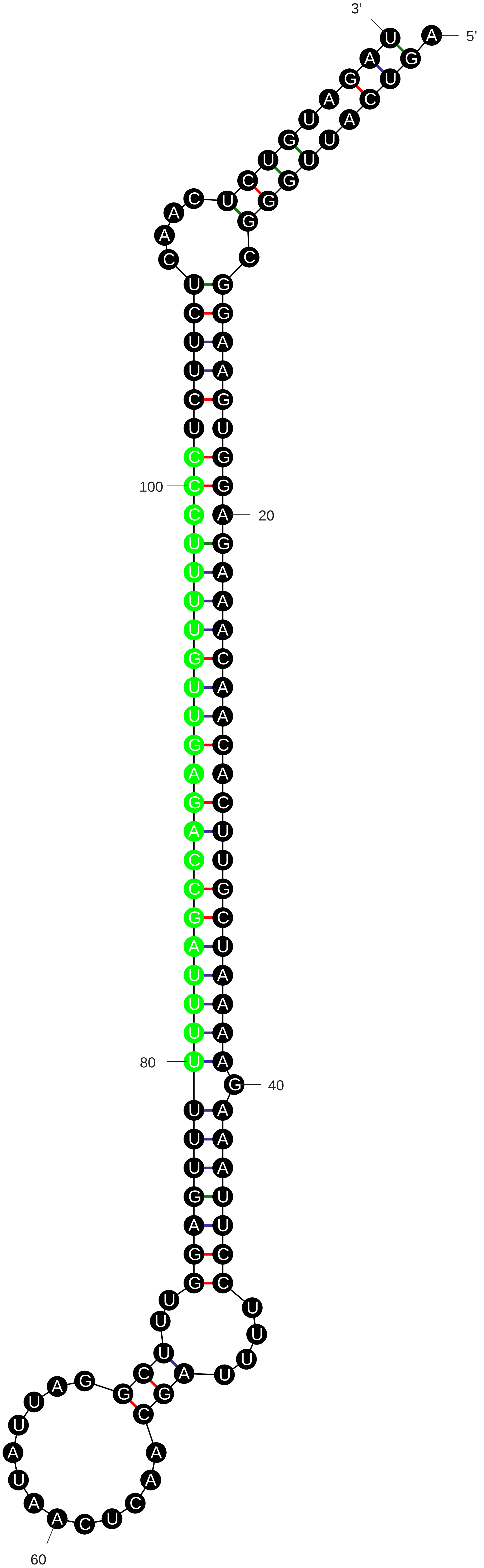

dG = -34.60 nta-miRn11b

Output of sir_graph (©)
mfold_util 4.6

Created Mon Oct 17 23:37:31 2011

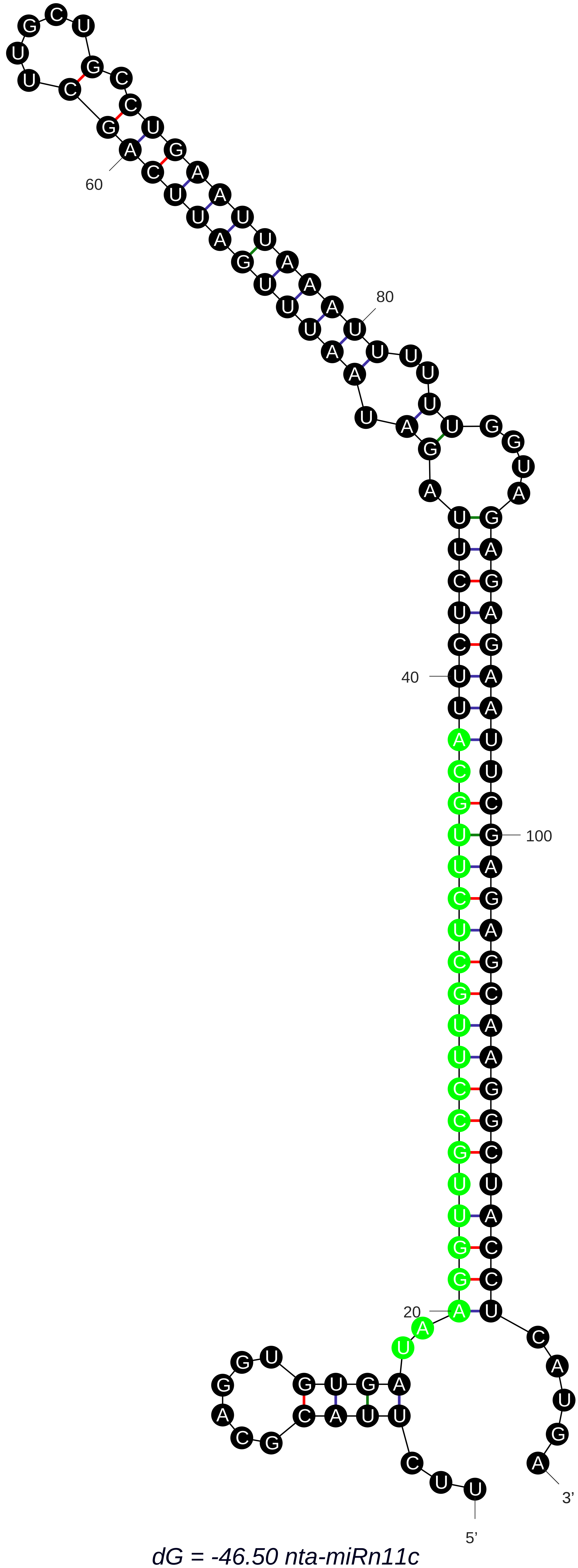

dG = -46.50 nta-miRn11c

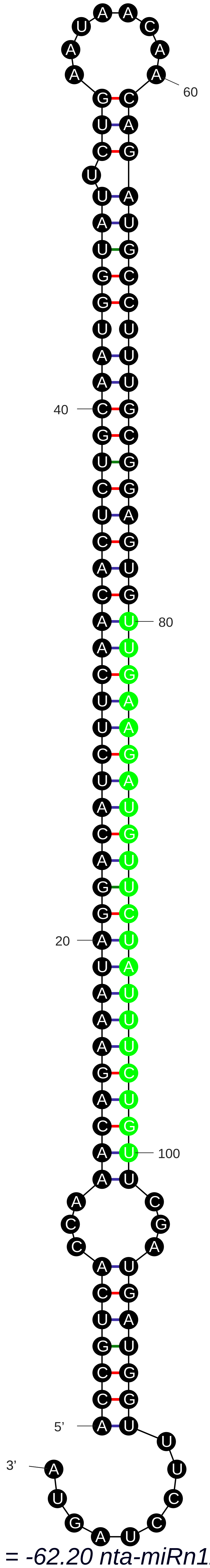

dG = -62.20 nta-miRn12

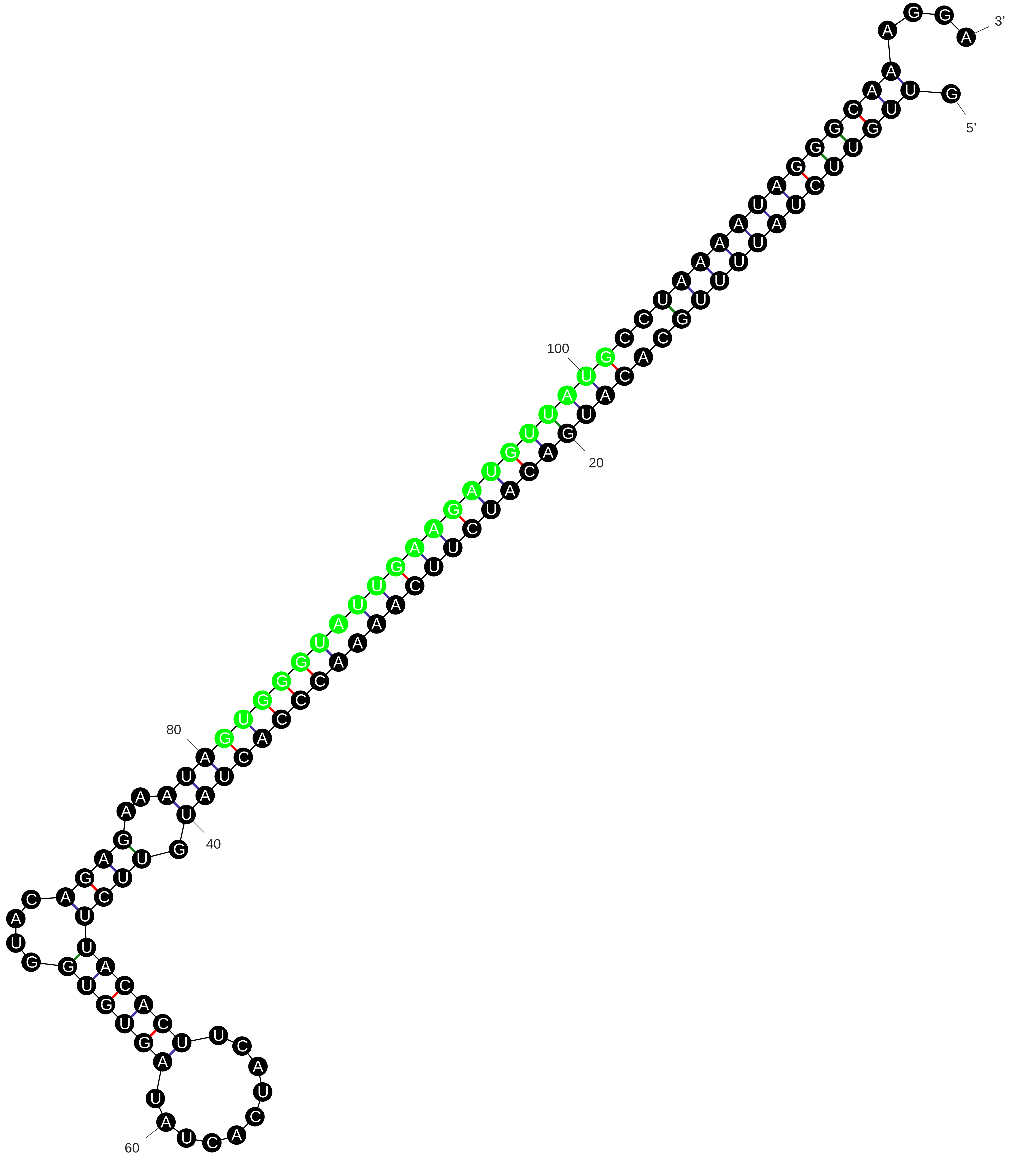

dG = -50.40 nta-miRn13a

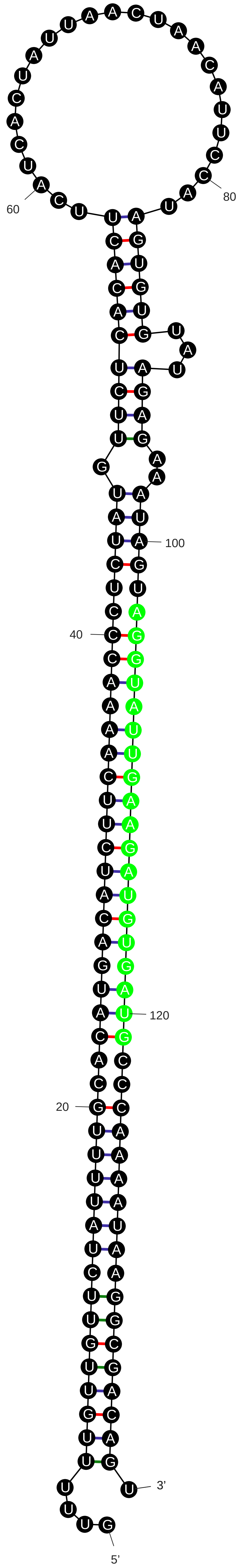

dG = -41.60 nta-miRn13b

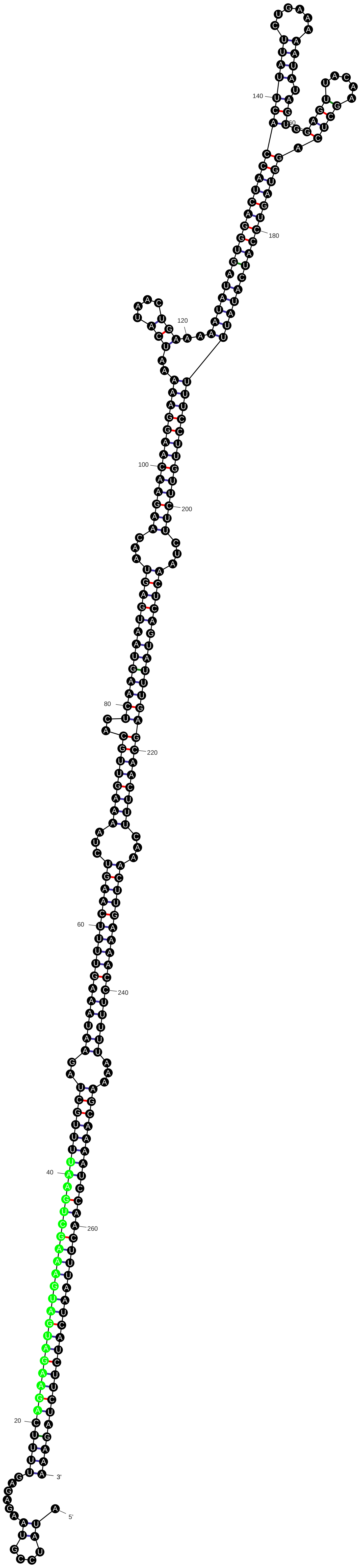

dG = -92.80 nta-miRn14

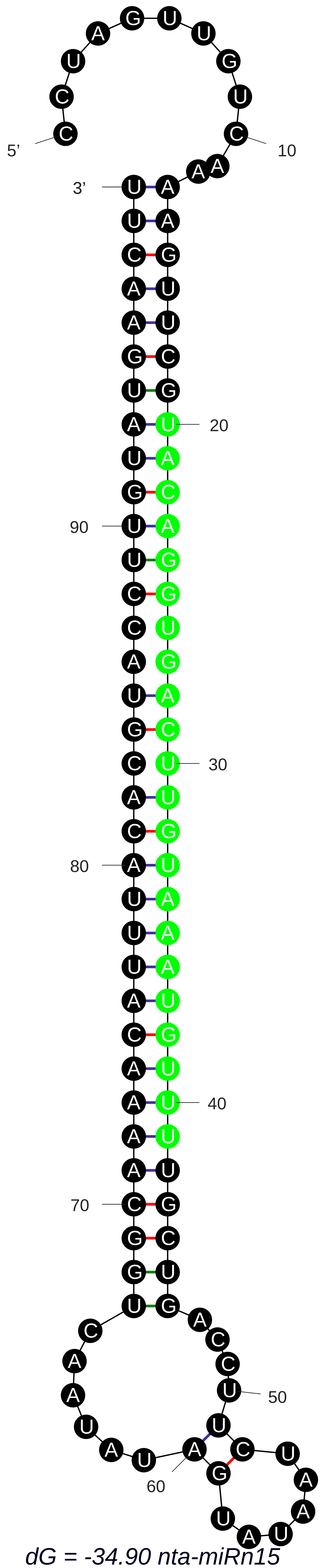

dG = -34.90 nta-miRn15

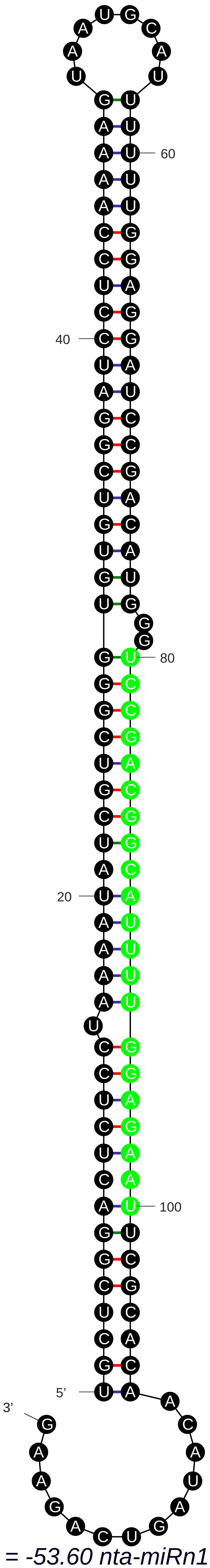

dG = -53.60 nta-miRn16

Output of sir_graph (©)
mfold_util 4.6

Created Tue Oct 18 00:08:44 2011

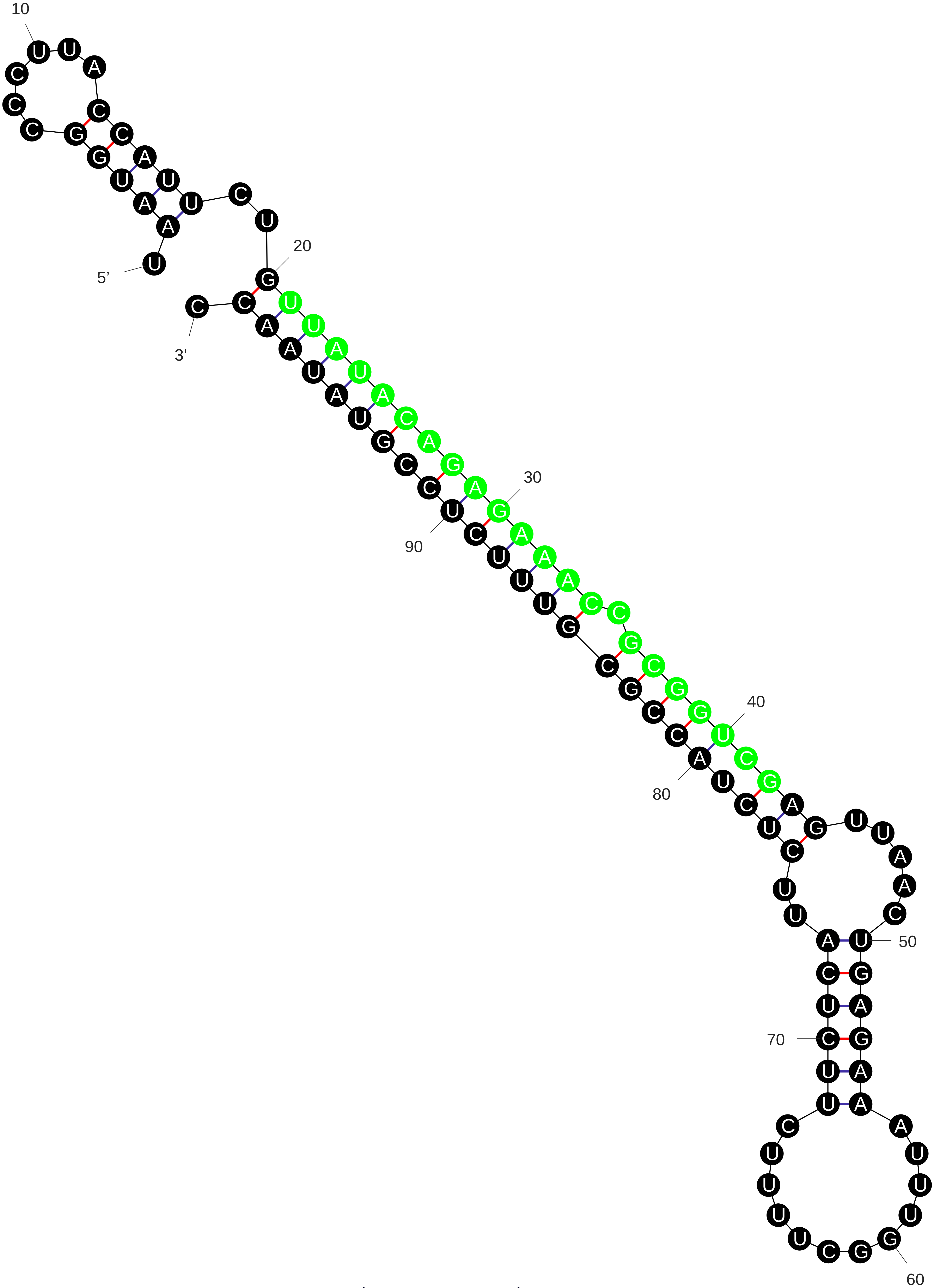

dG = -34.50 nta-miRn17

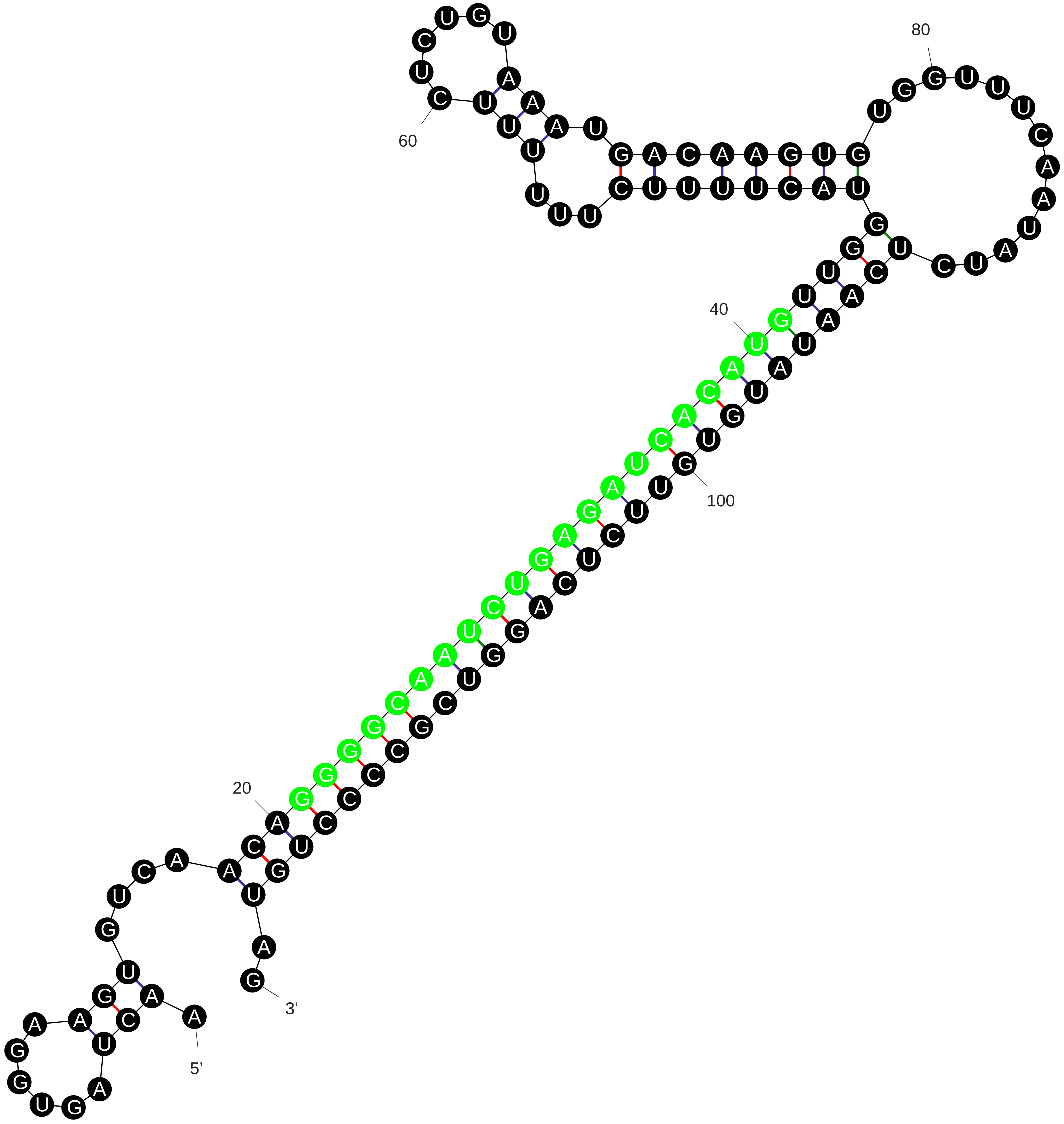

dG = -40.70 nta-miRn18

Output of sir_graph (©)
mfold_util 4.6

Created Tue Oct 18 02:46:38 2011

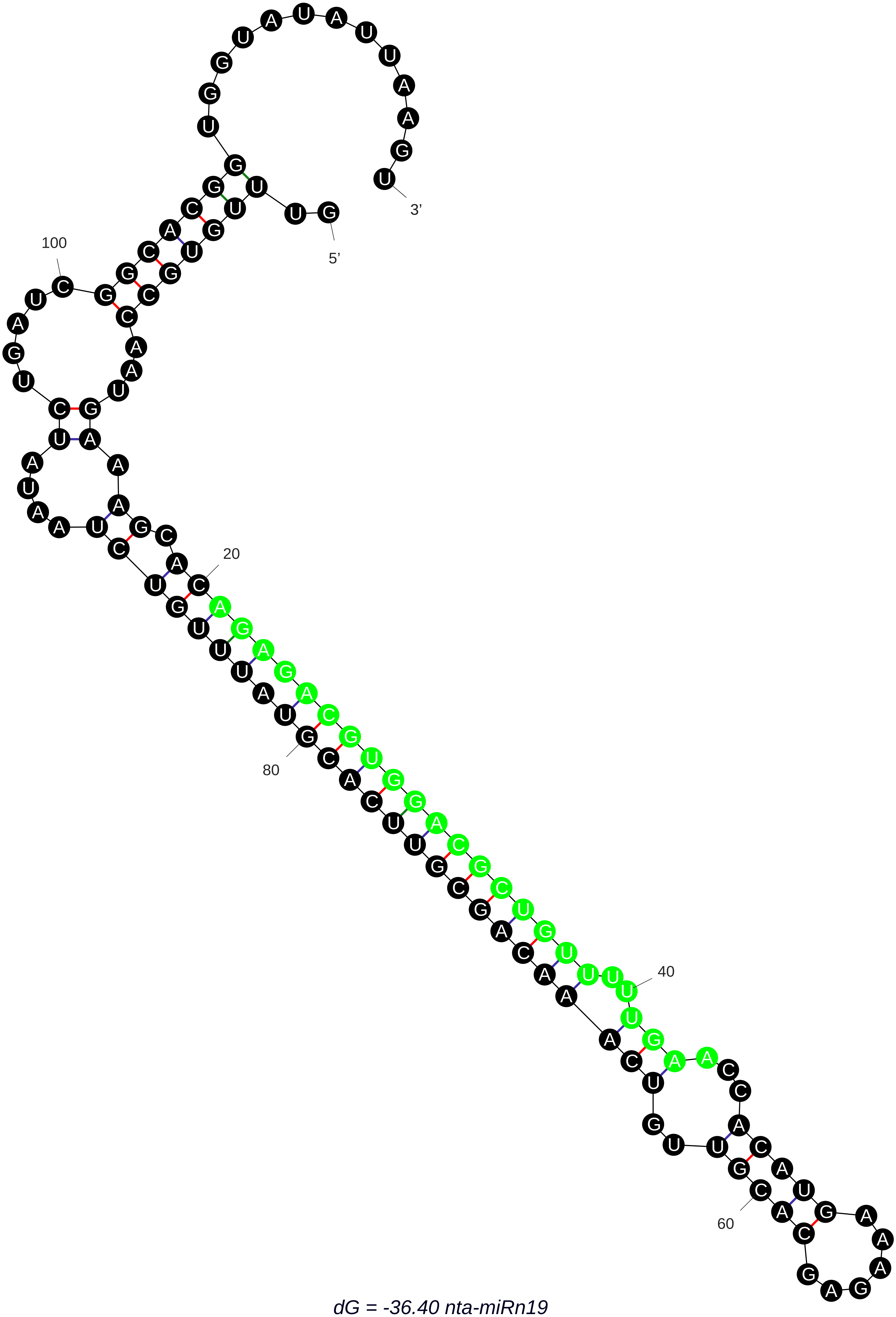

dG = -36.40 nta-miRn19

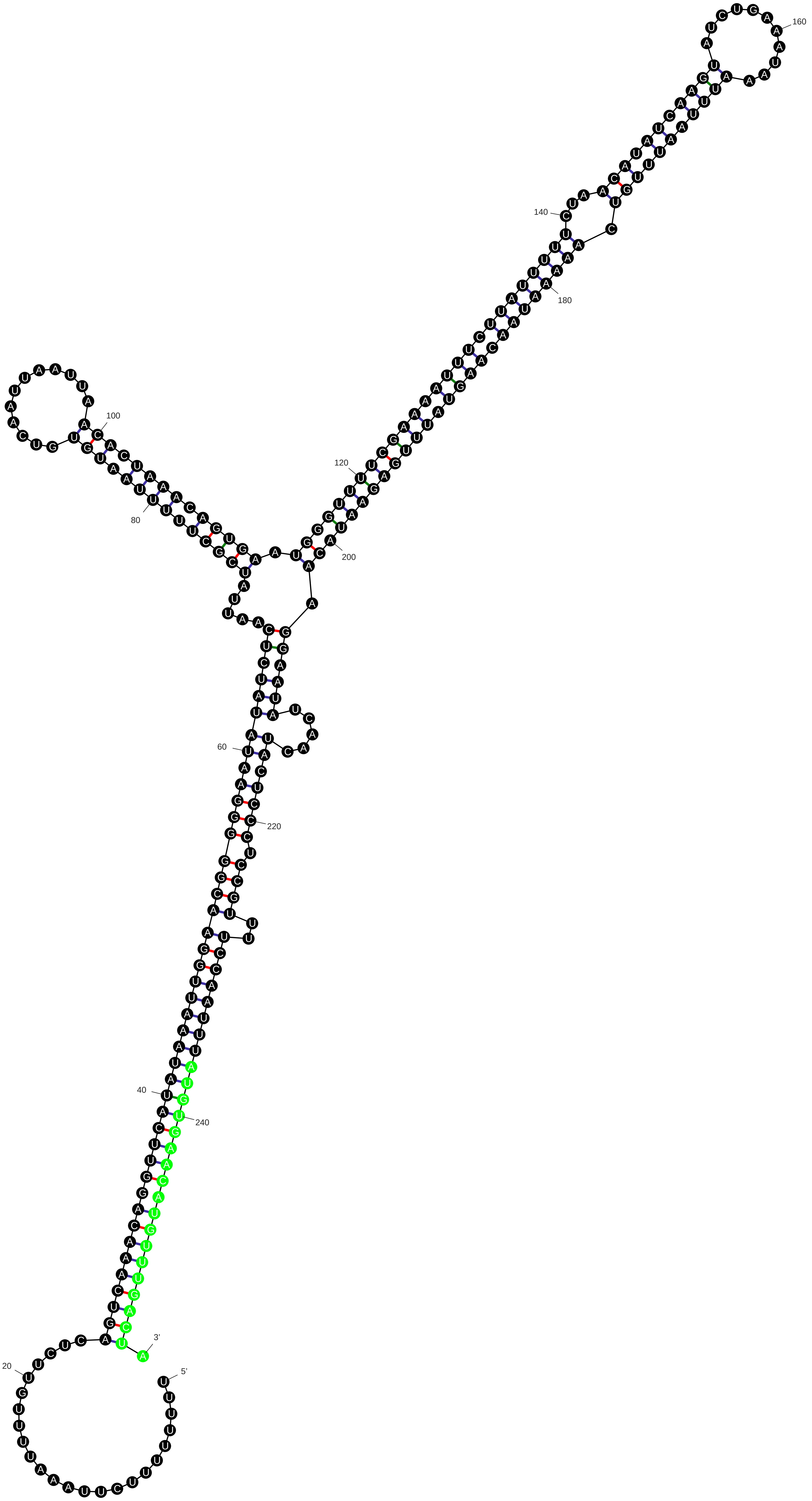

dG = -64.10 nta-miRn20

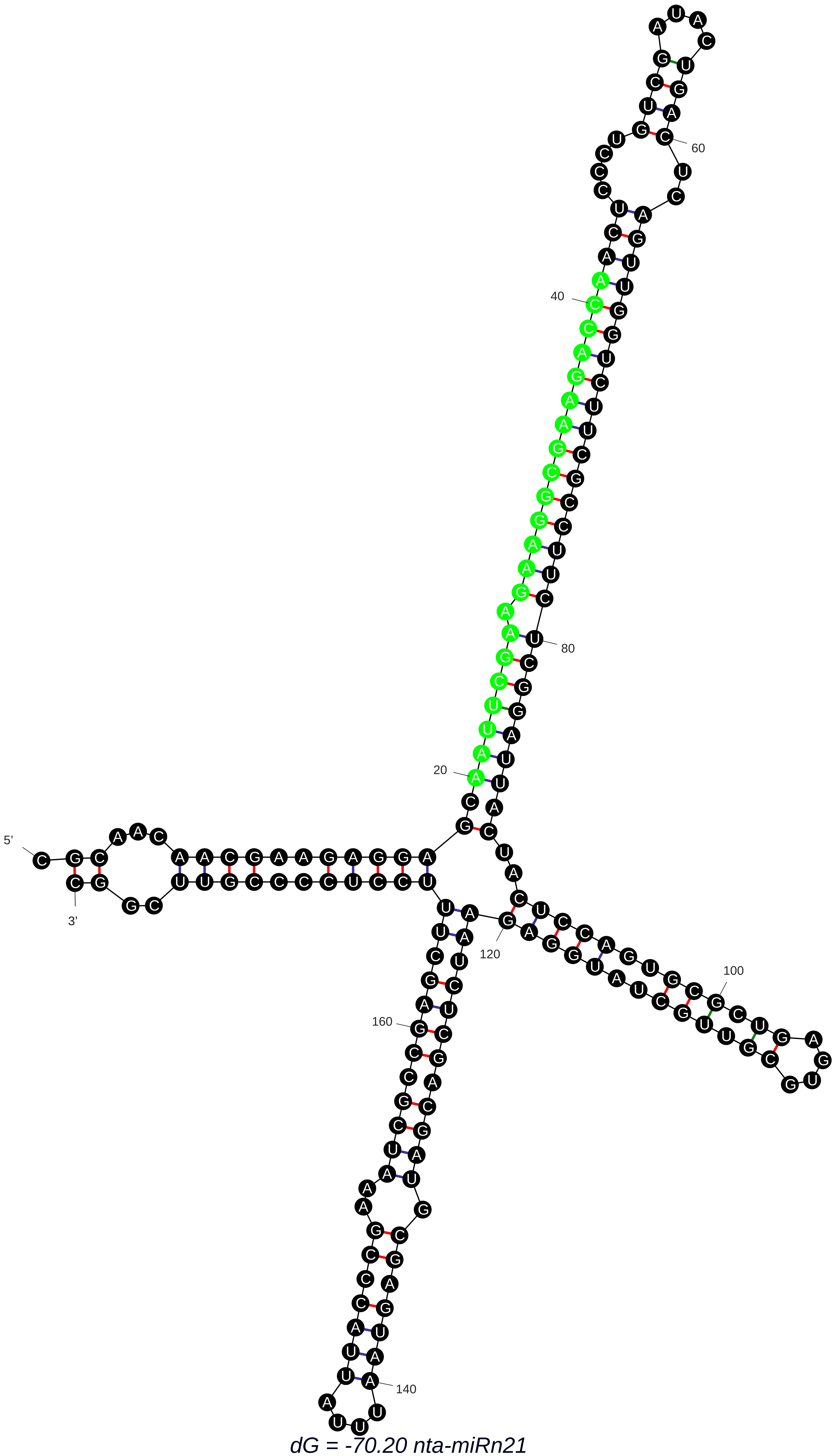

dG = -70.20 nta-miRn21

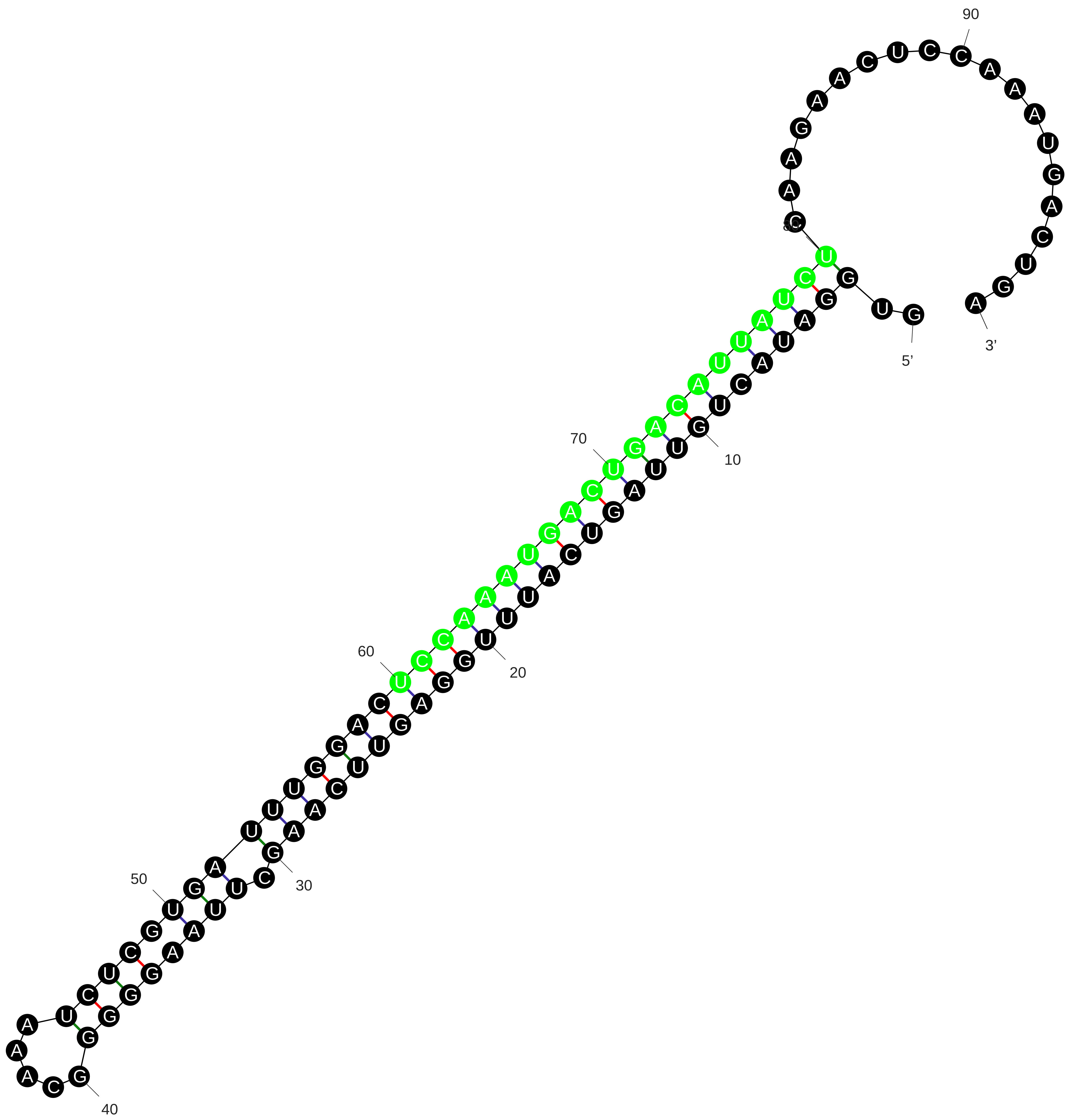

dG = -39.00 nta-miRn22

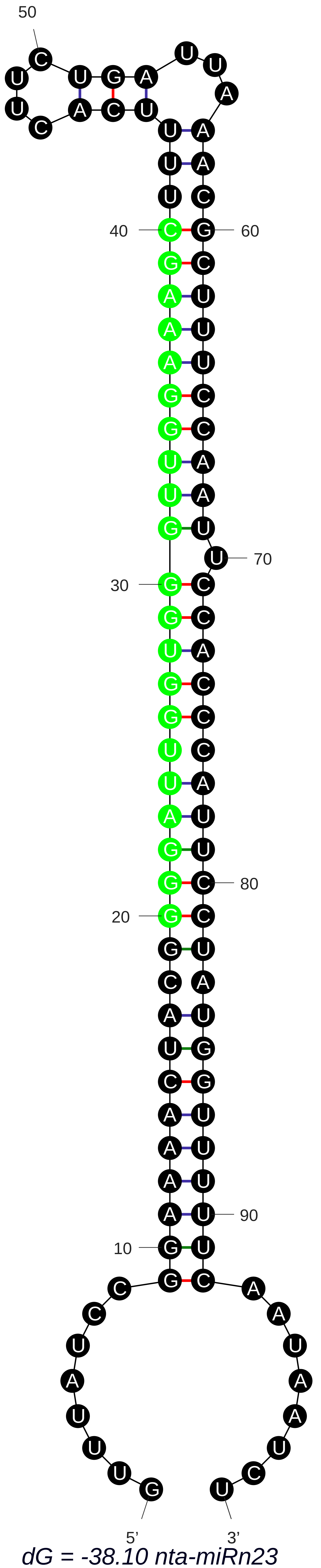

dG = -38.10 nta-miRn23

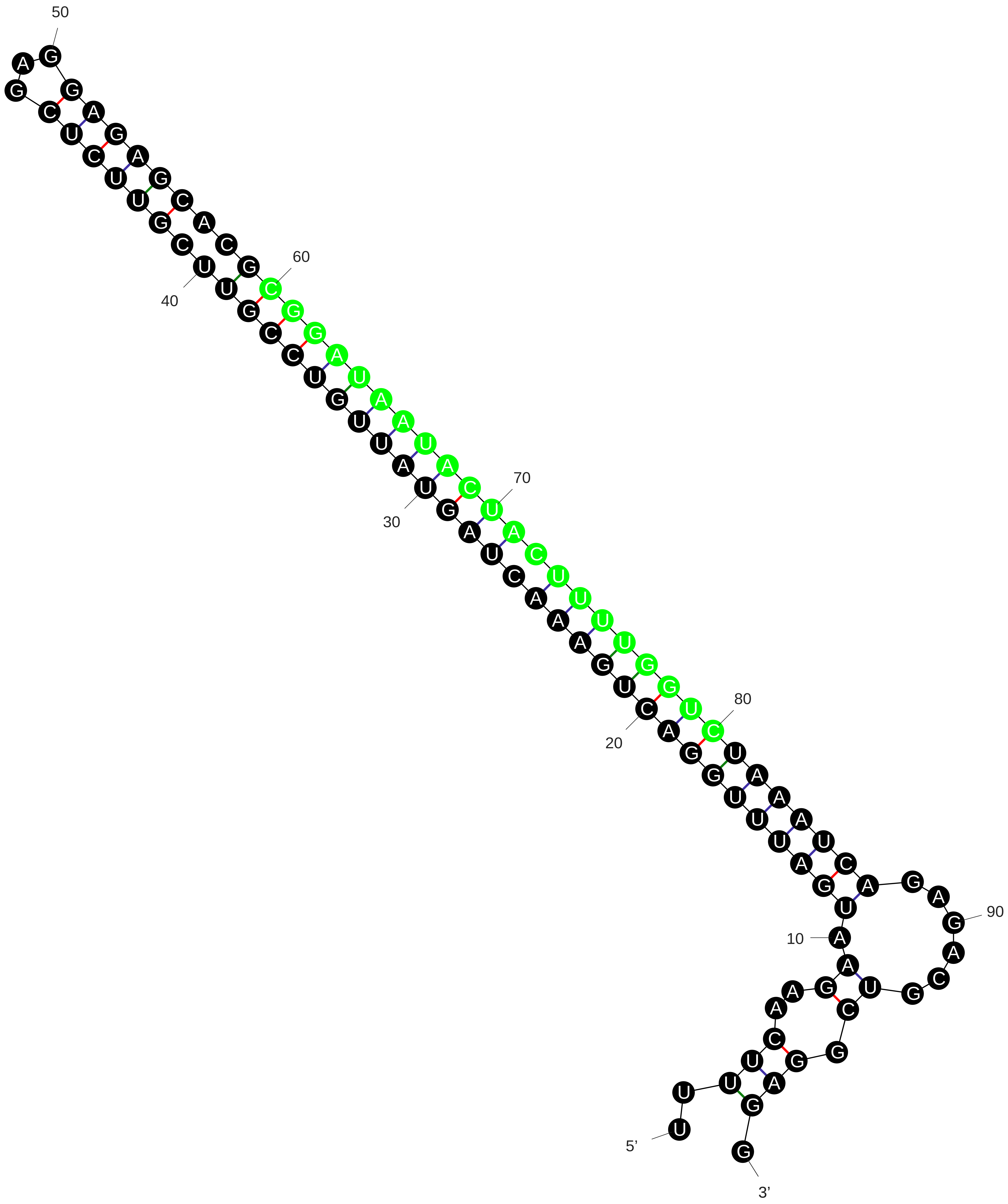

dG = -42.50 nta-miRn24

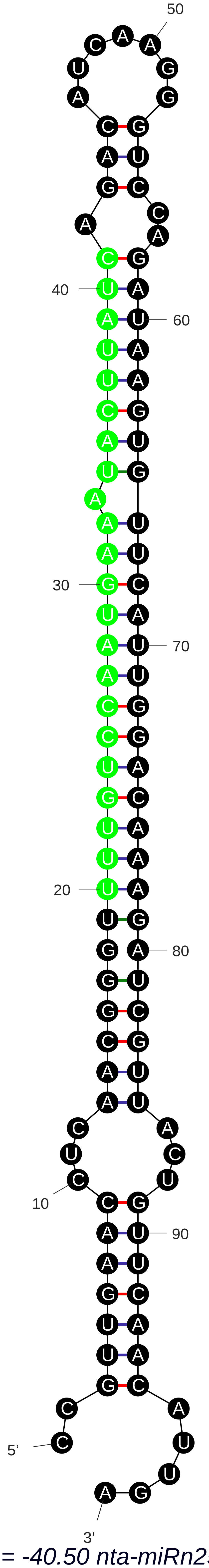

dG = -40.50 nta-miRn25

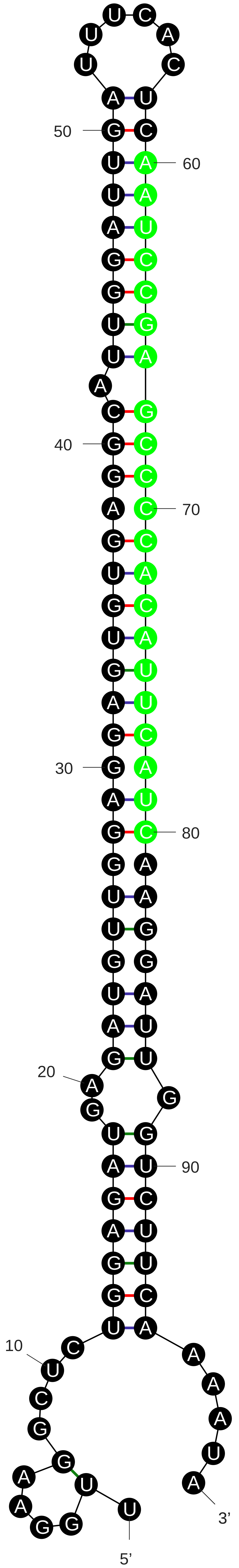

dG = -32.80 nta-miRn26a

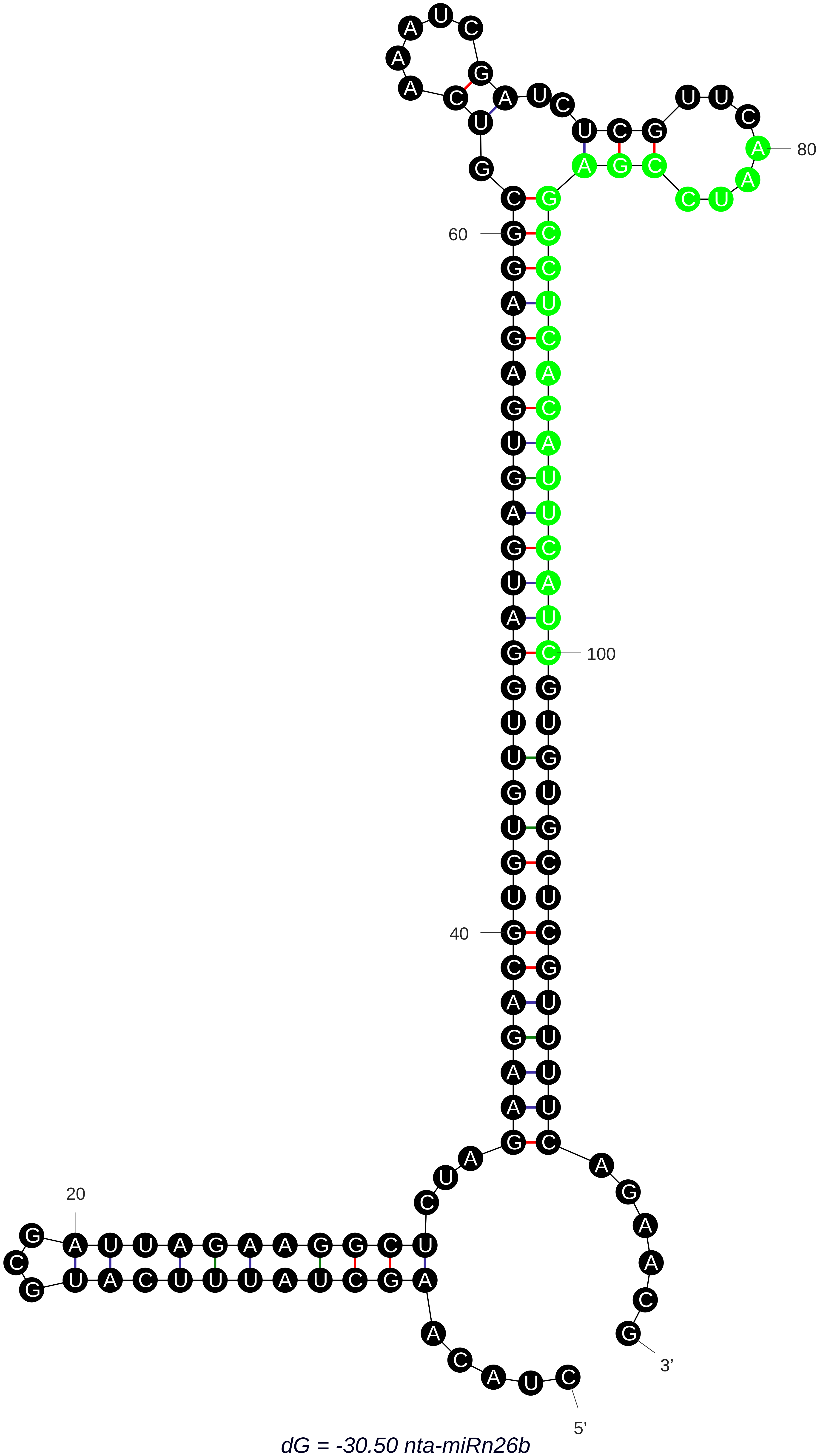

dG = -30.50 nta-miRn26b

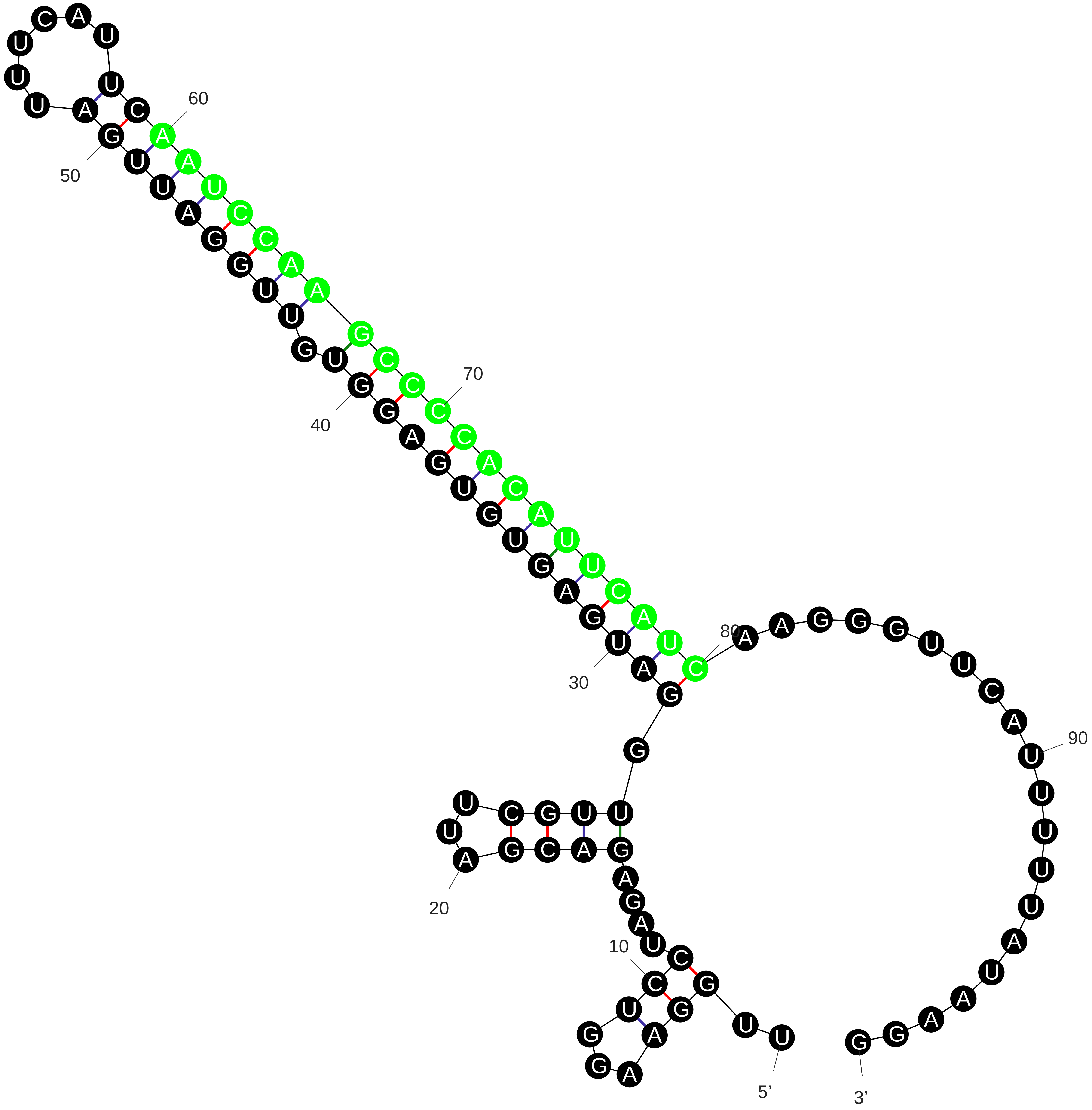

dG = -31.70 nta-miRn26c

Output of sir_graph (©)
mfold_util 4.6

Created Tue Oct 18 03:10:36 2011

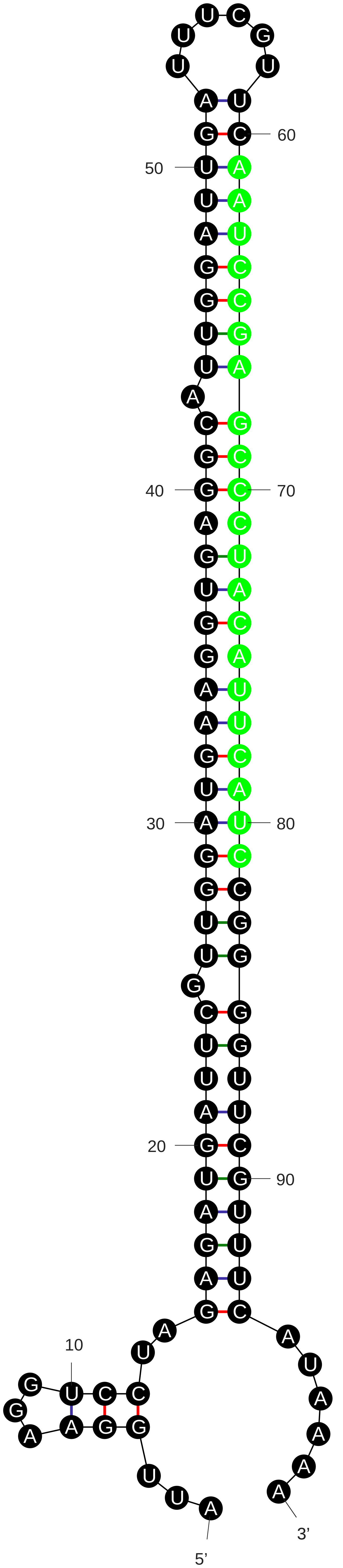

dG = -38.80 nta-miRn26d

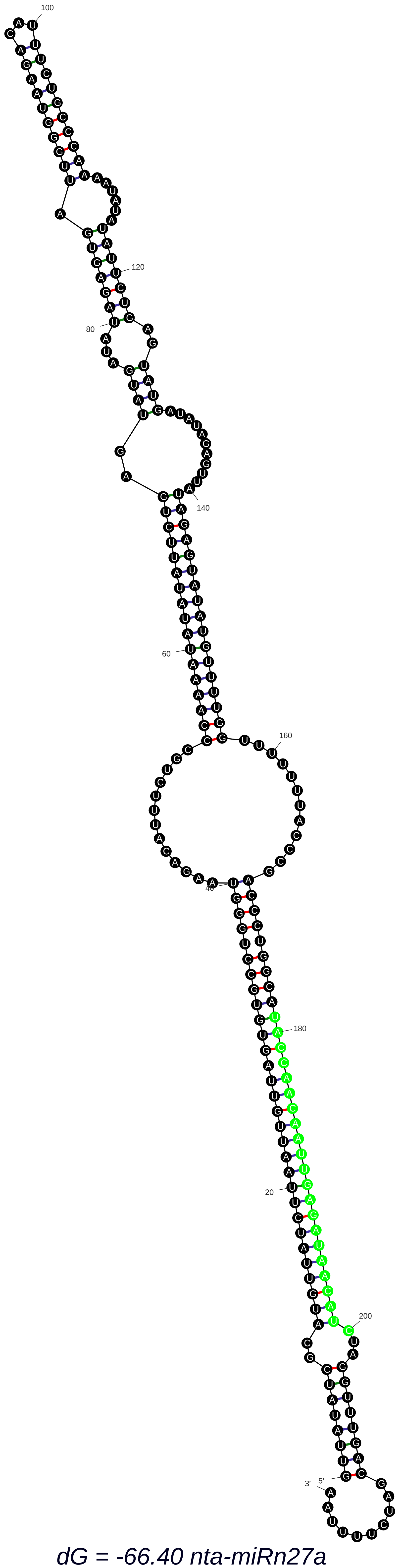

dG = -66.40 nta-miRn27a

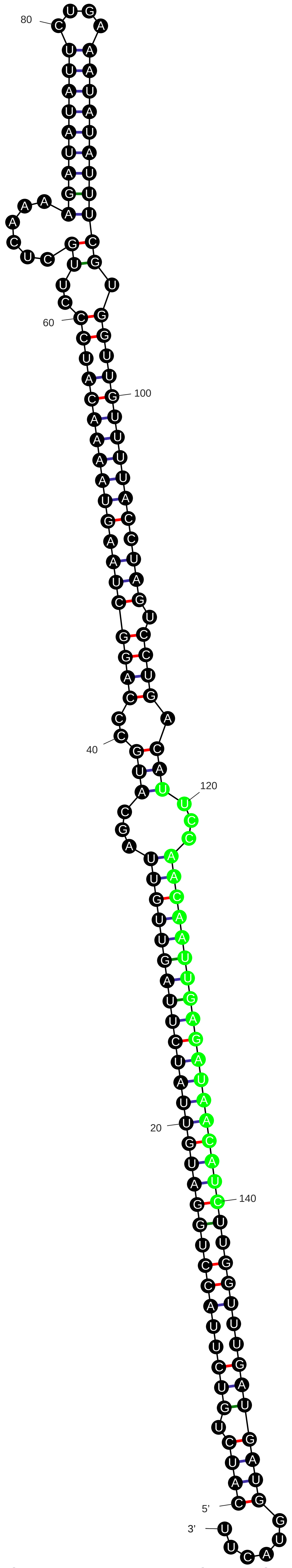

dG = -50.10 nta-miRn27b

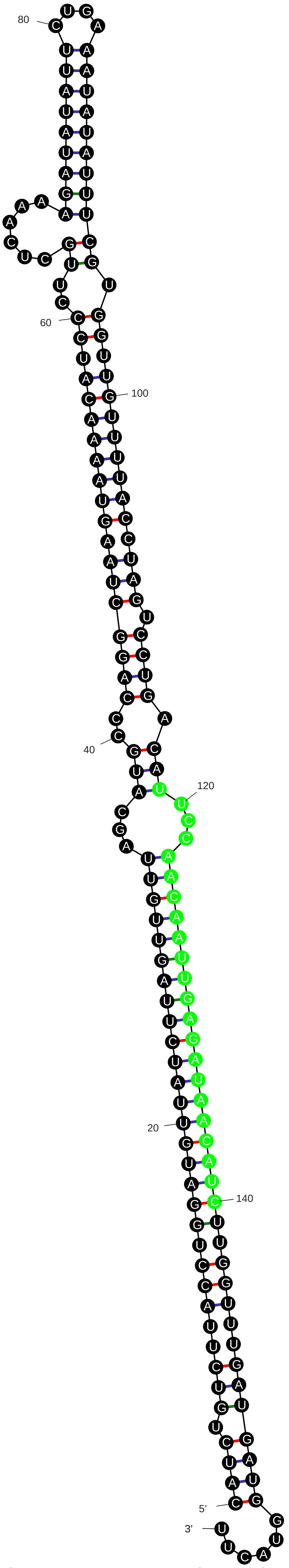

dG = -50.10 nta-miRn27b

Output of sir_graph (©)
mfold_util 4.6

Created Tue Oct 18 03:17:30 2011

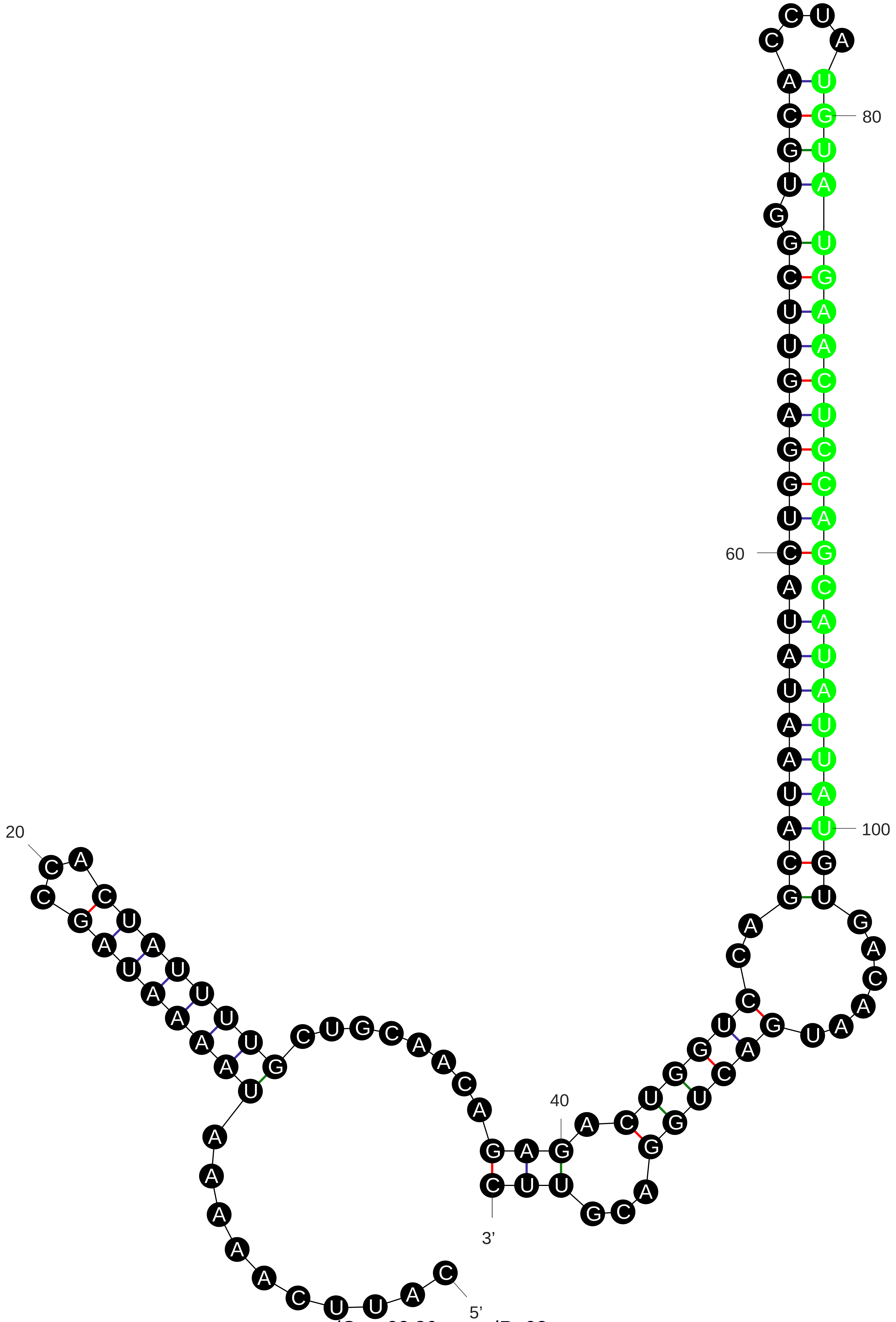

dG = -33.80 nta-miRn28

Output of sir_graph (©)
mfold_util 4.6

Created Tue Oct 18 03:20:22 2011

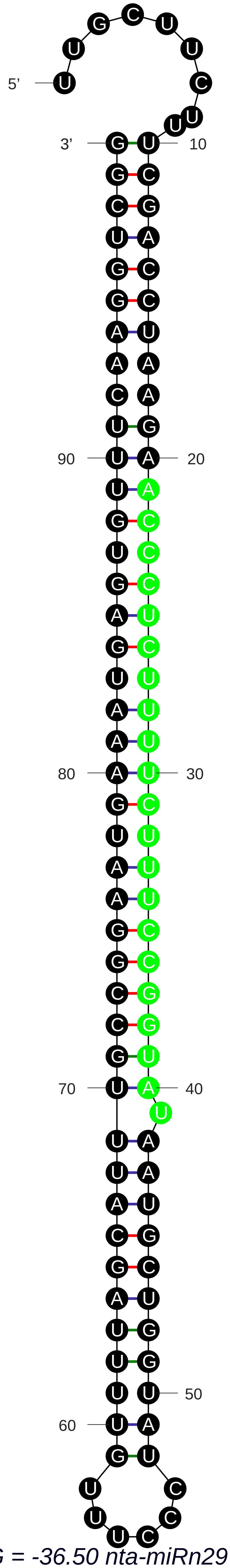

dG = -36.50 nta-miRn29

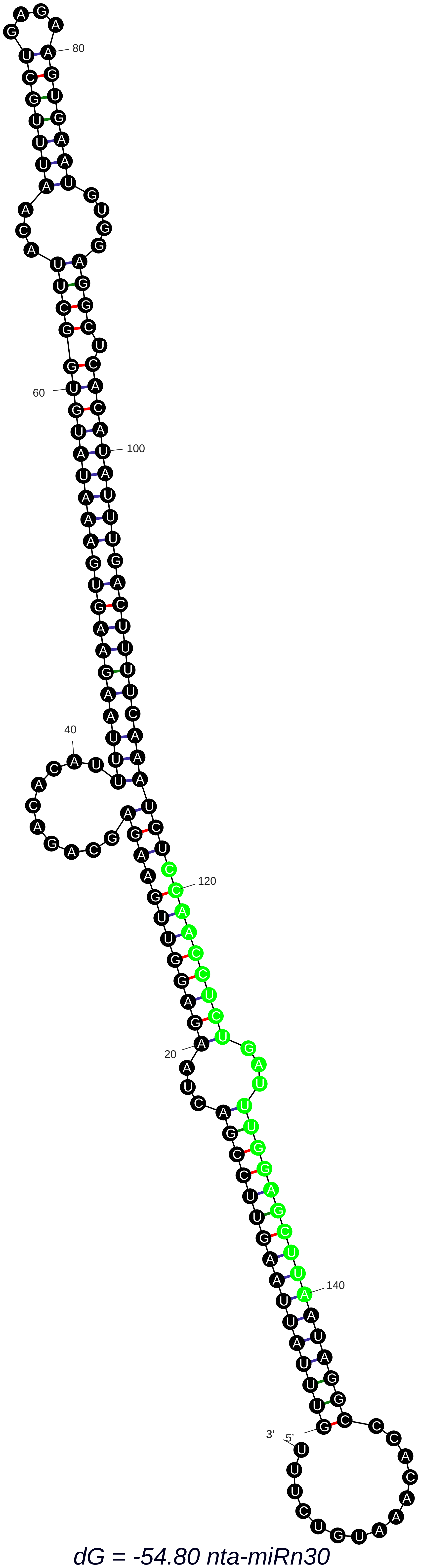

dG = -54.80 nta-miRn30

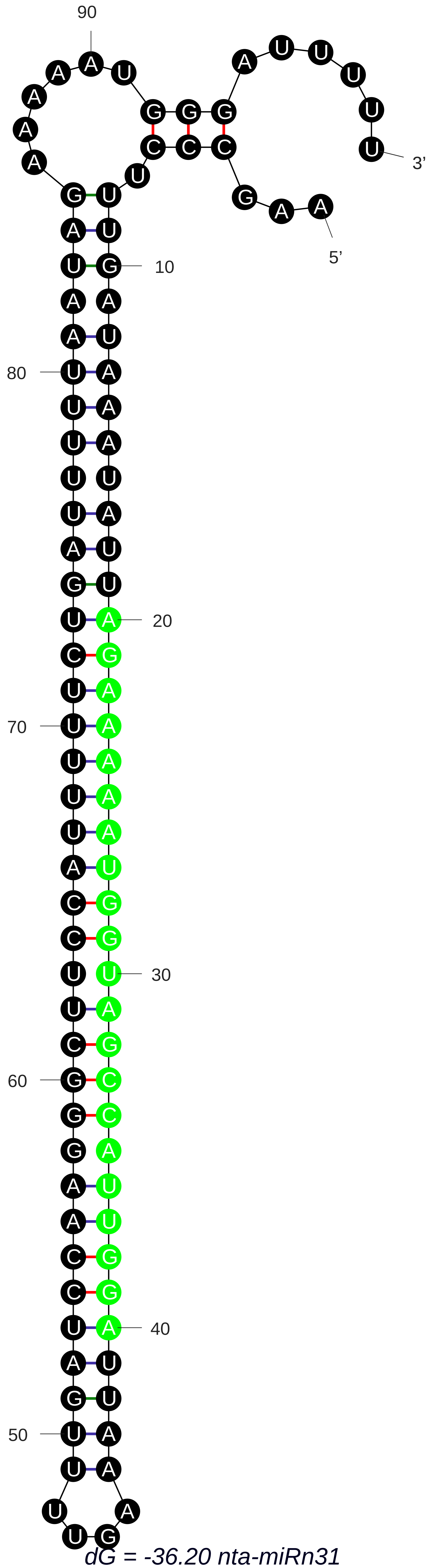

dG = -36.20 nta-miRn31

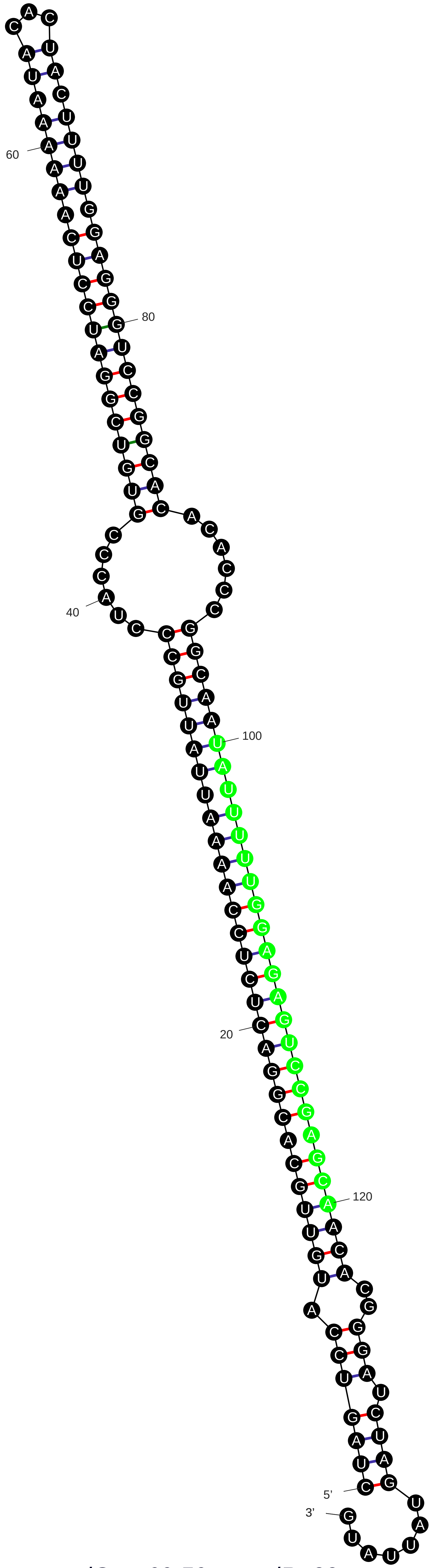

dG = -69.50 nta-miRn32

Output of sir_graph (©)
mfold_util 4.6

Created Tue Oct 18 03:30:13 2011

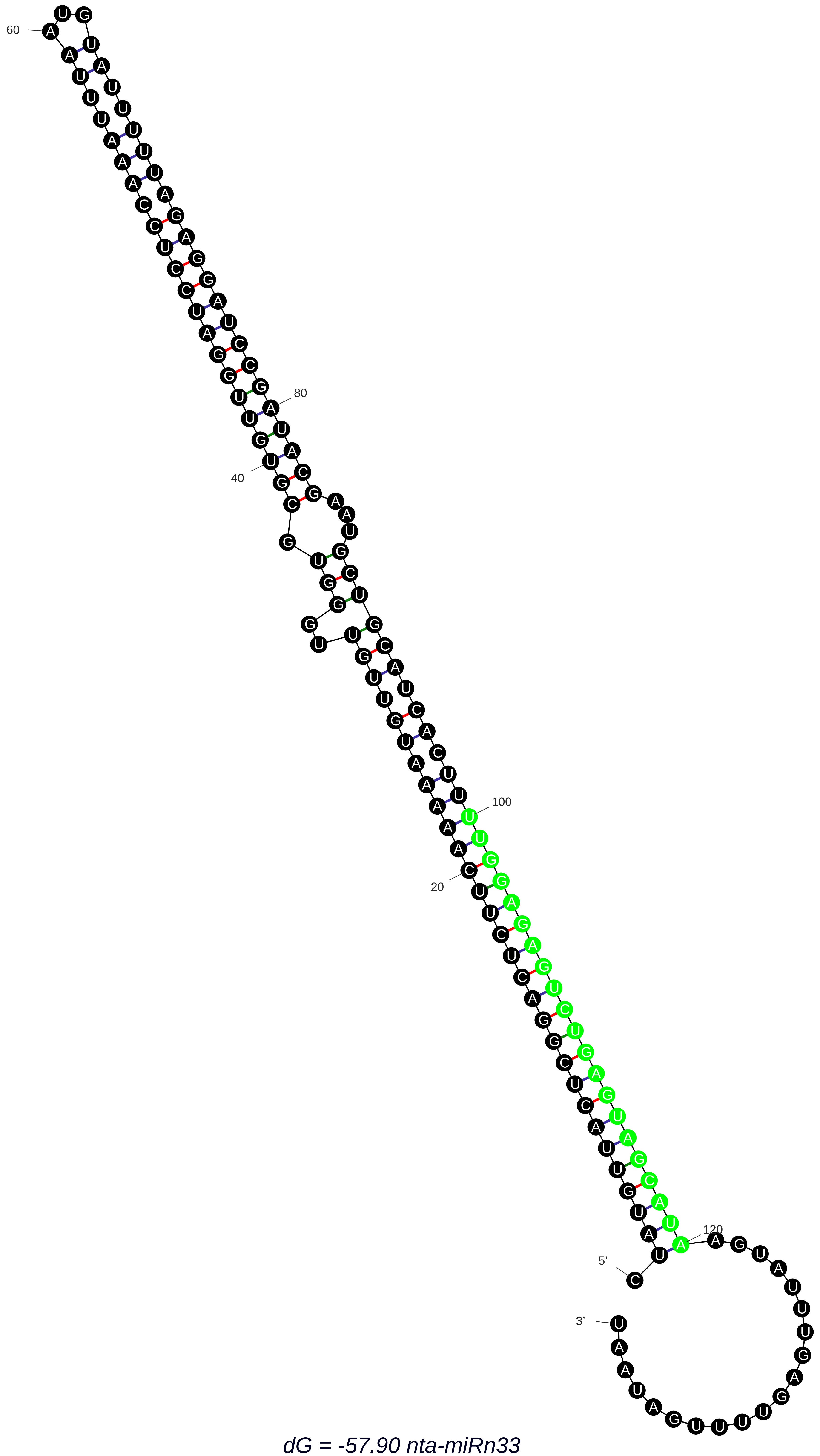

dG = -57.90 nta-miRn33

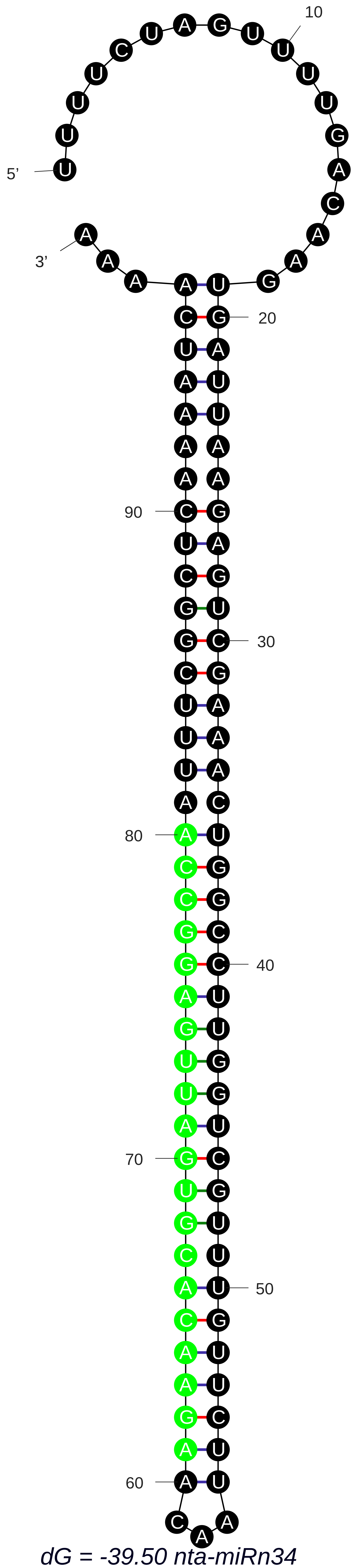

dG = -39.50 nta-miRn34

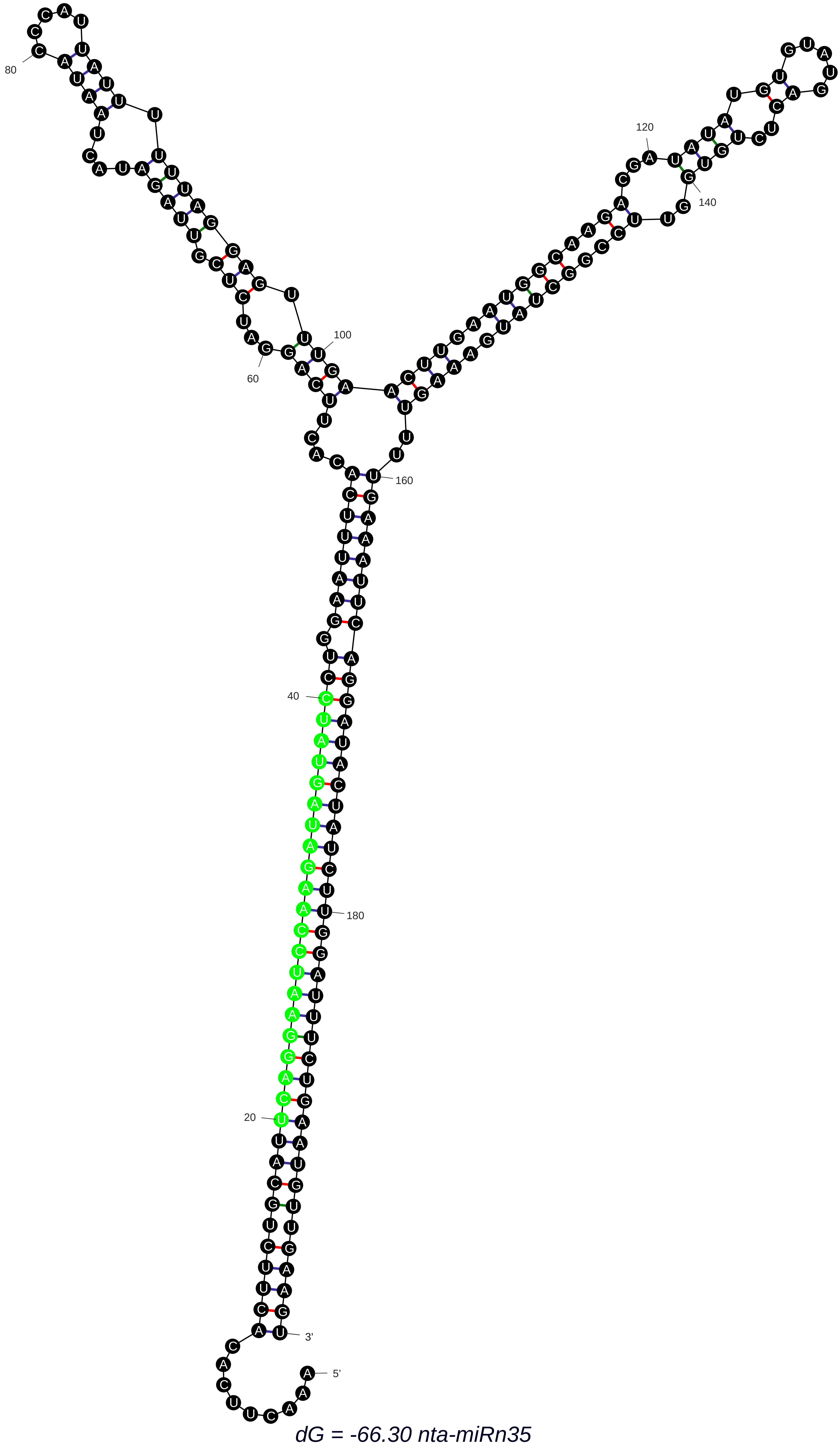

dG = -66.30 nta-miRn35

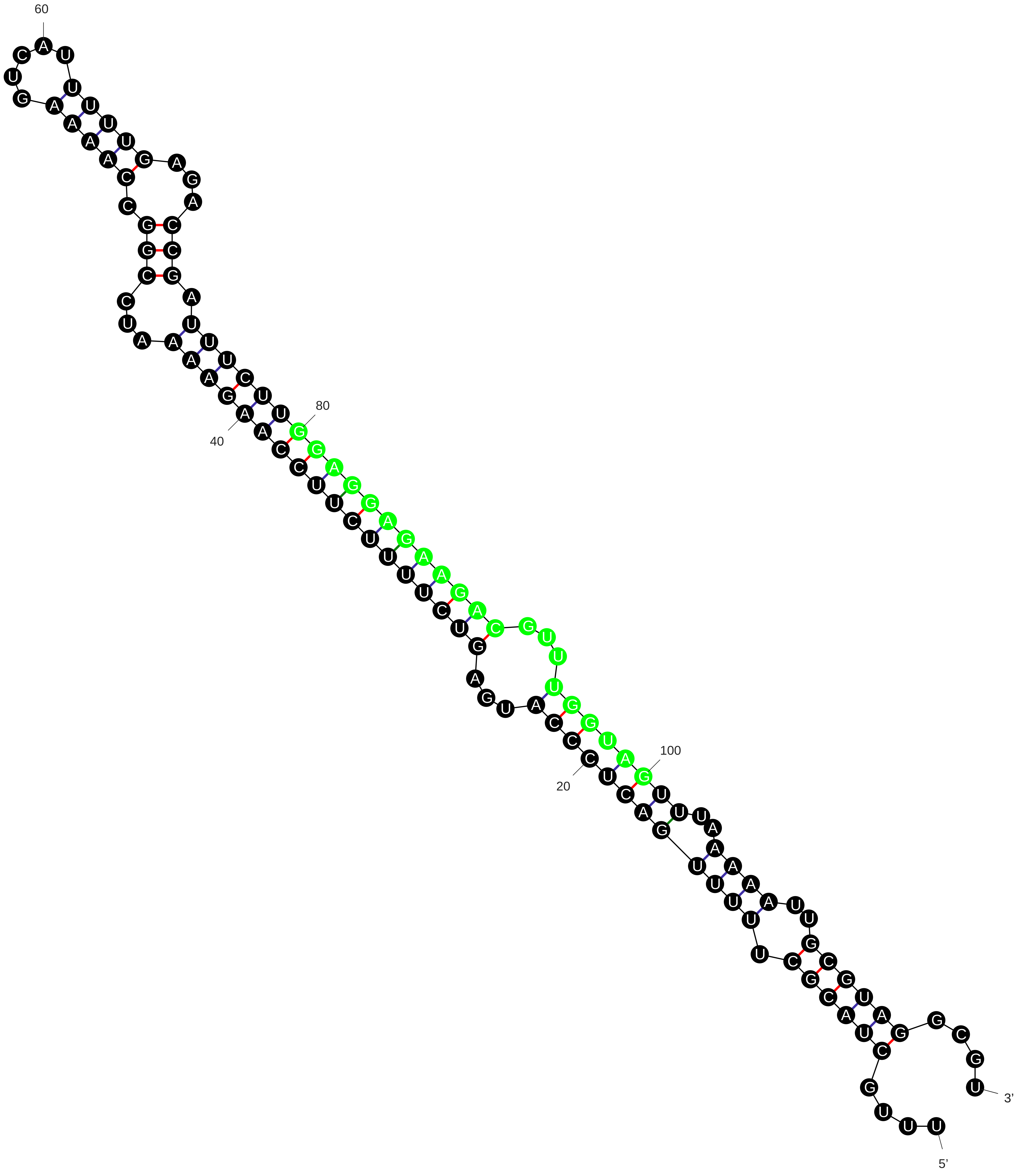

dG = -45.80 nta-miRn36

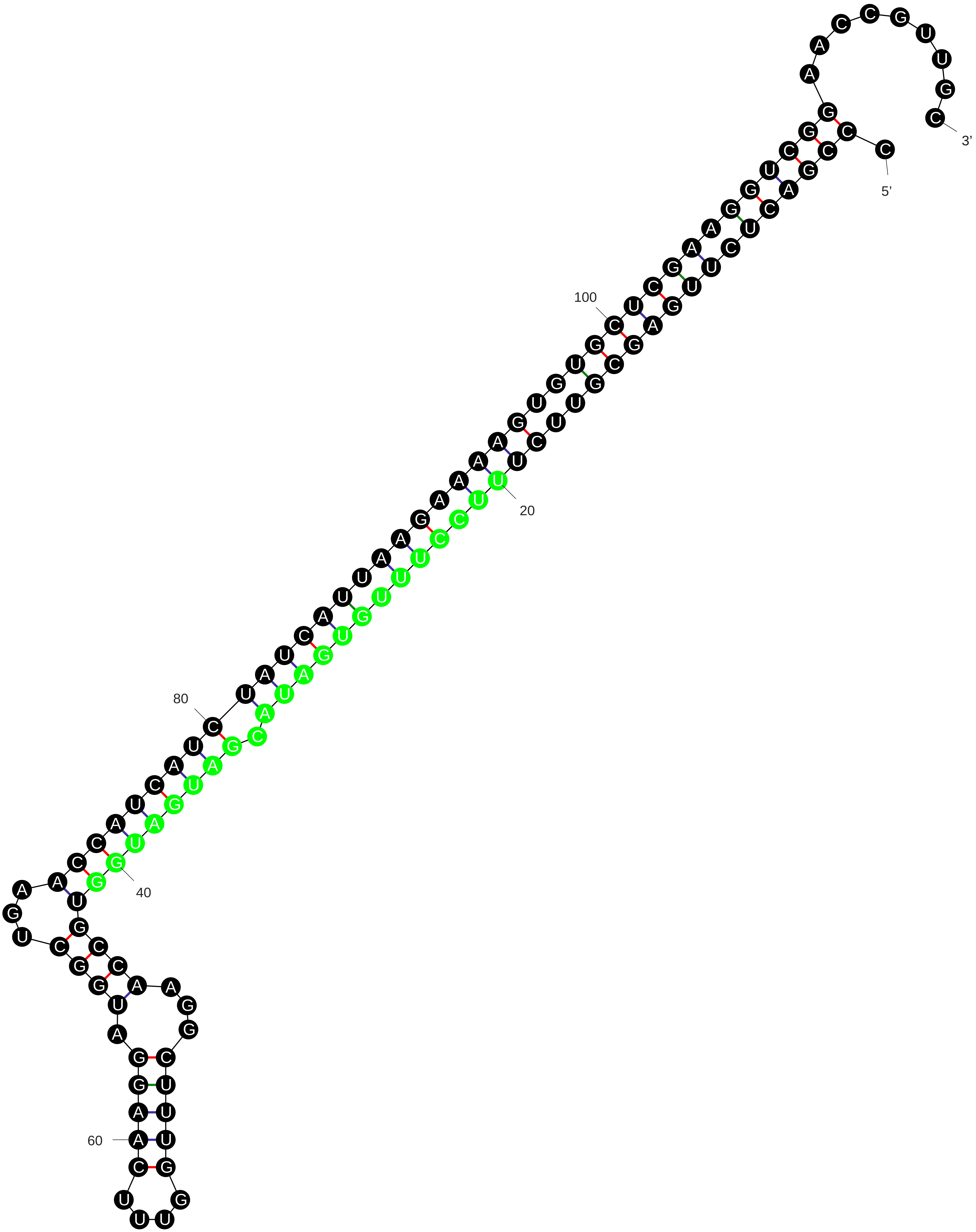

dG = -50.00 nta-miRn37

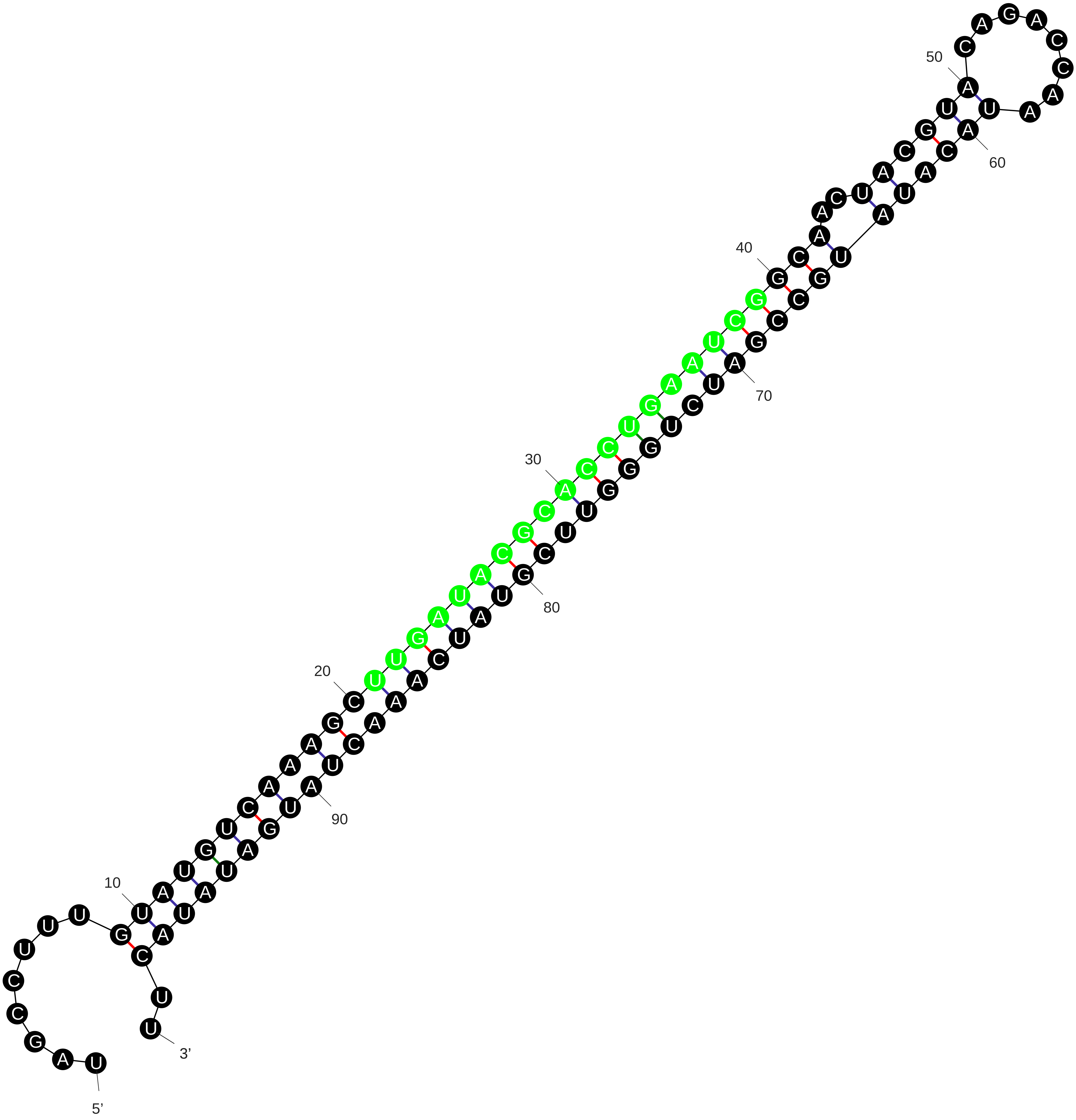

dG = -37.20 nta-miRn38

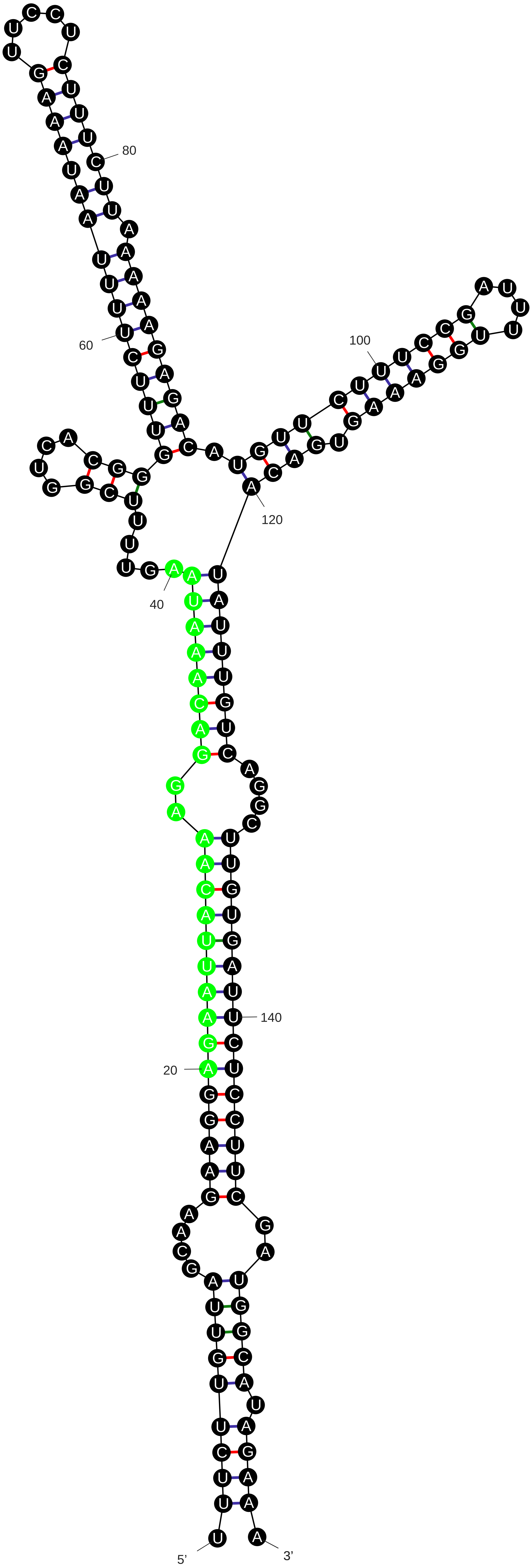

dG = -50.70 nta-miRn39

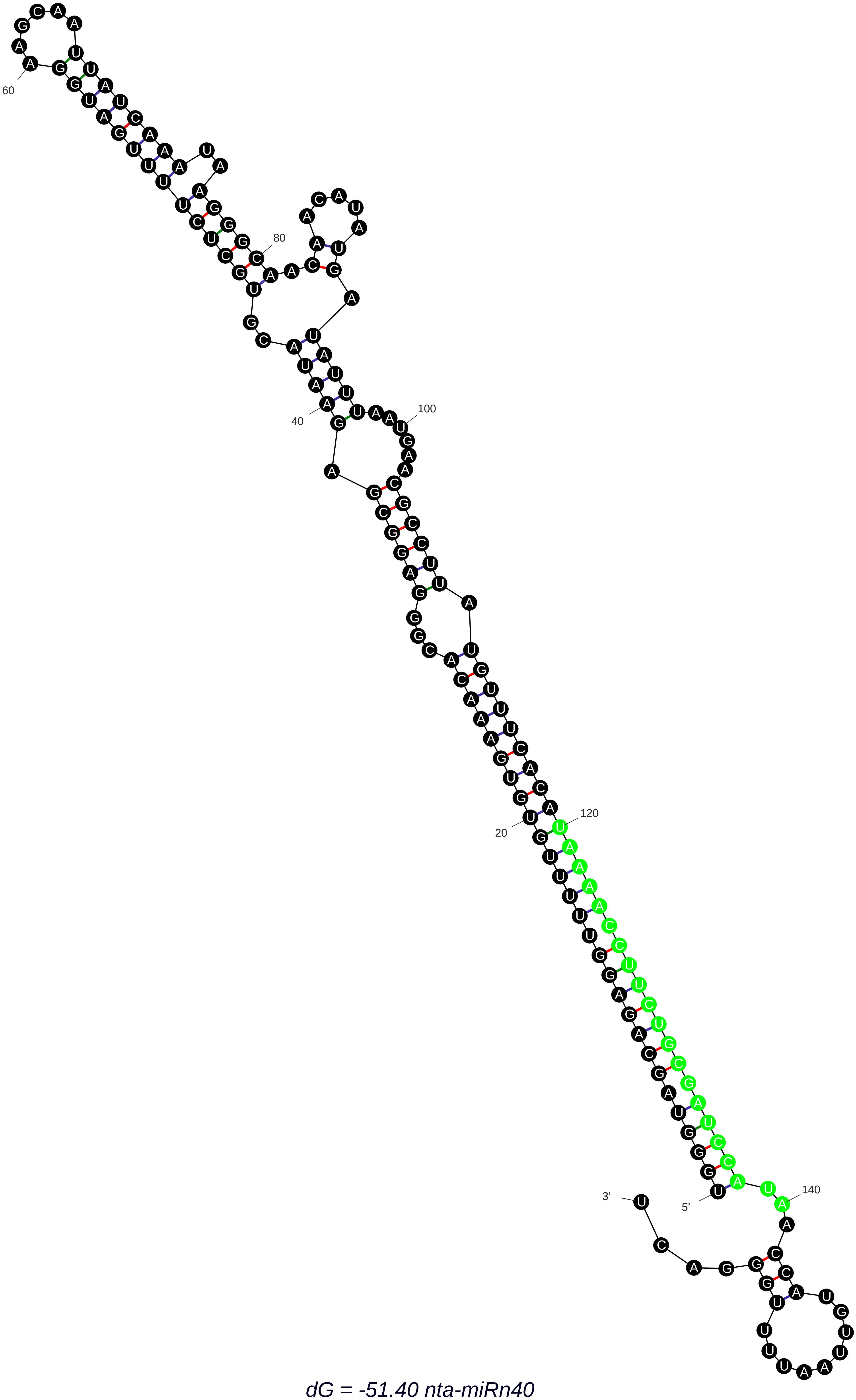

dG = -51.40 nta-miRn40

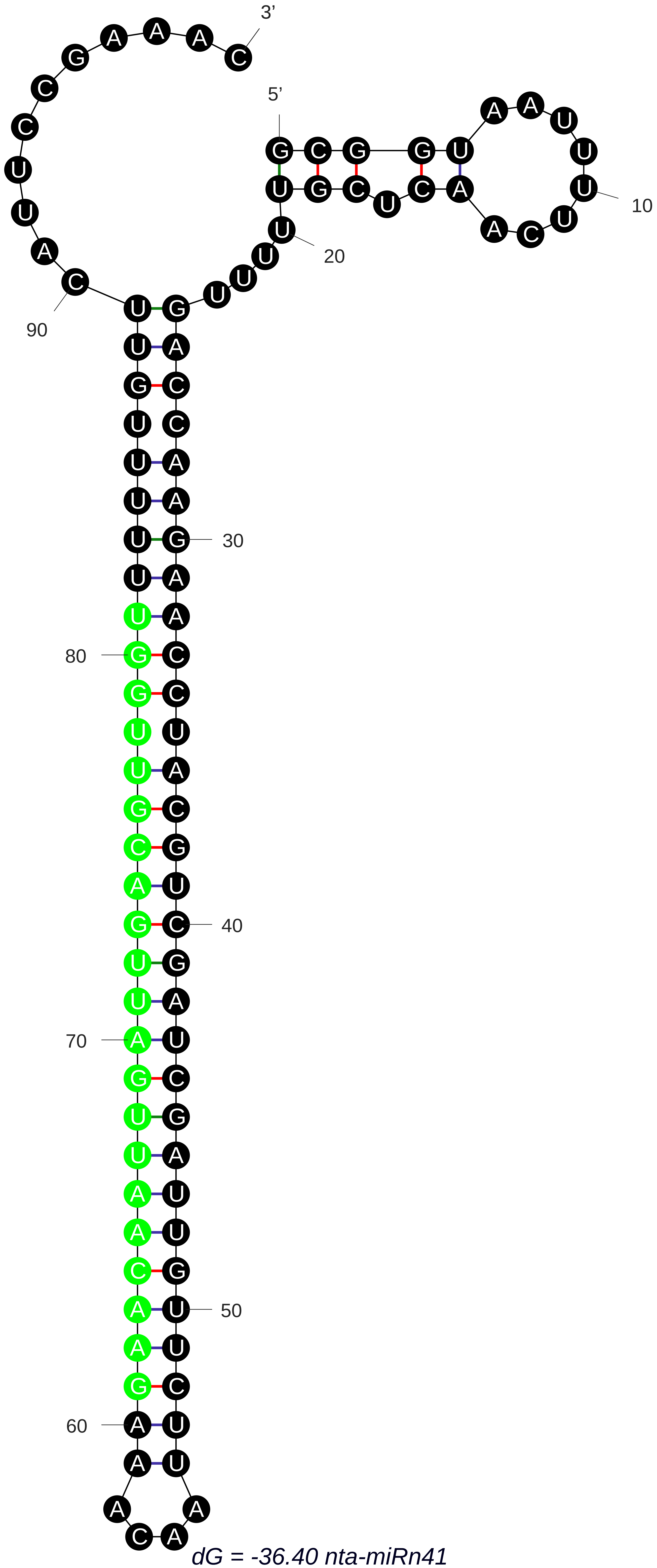

dG = -36.40 nta-miRn41

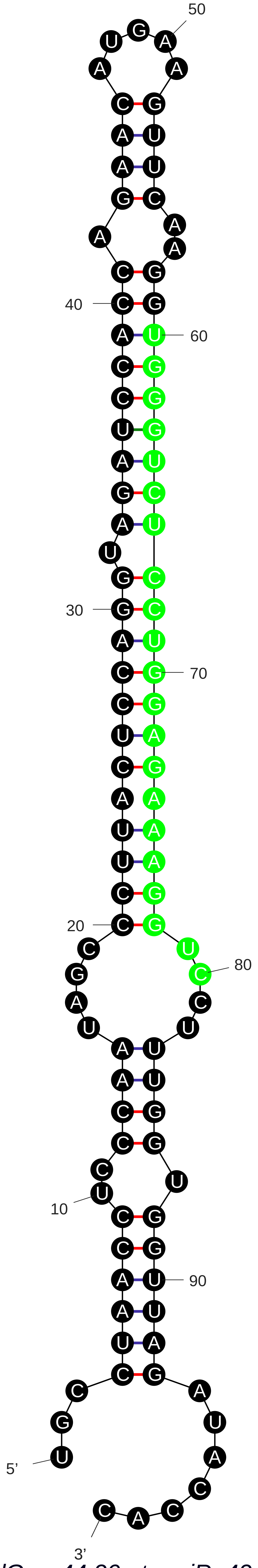

dG = -44.20 nta-miRn42

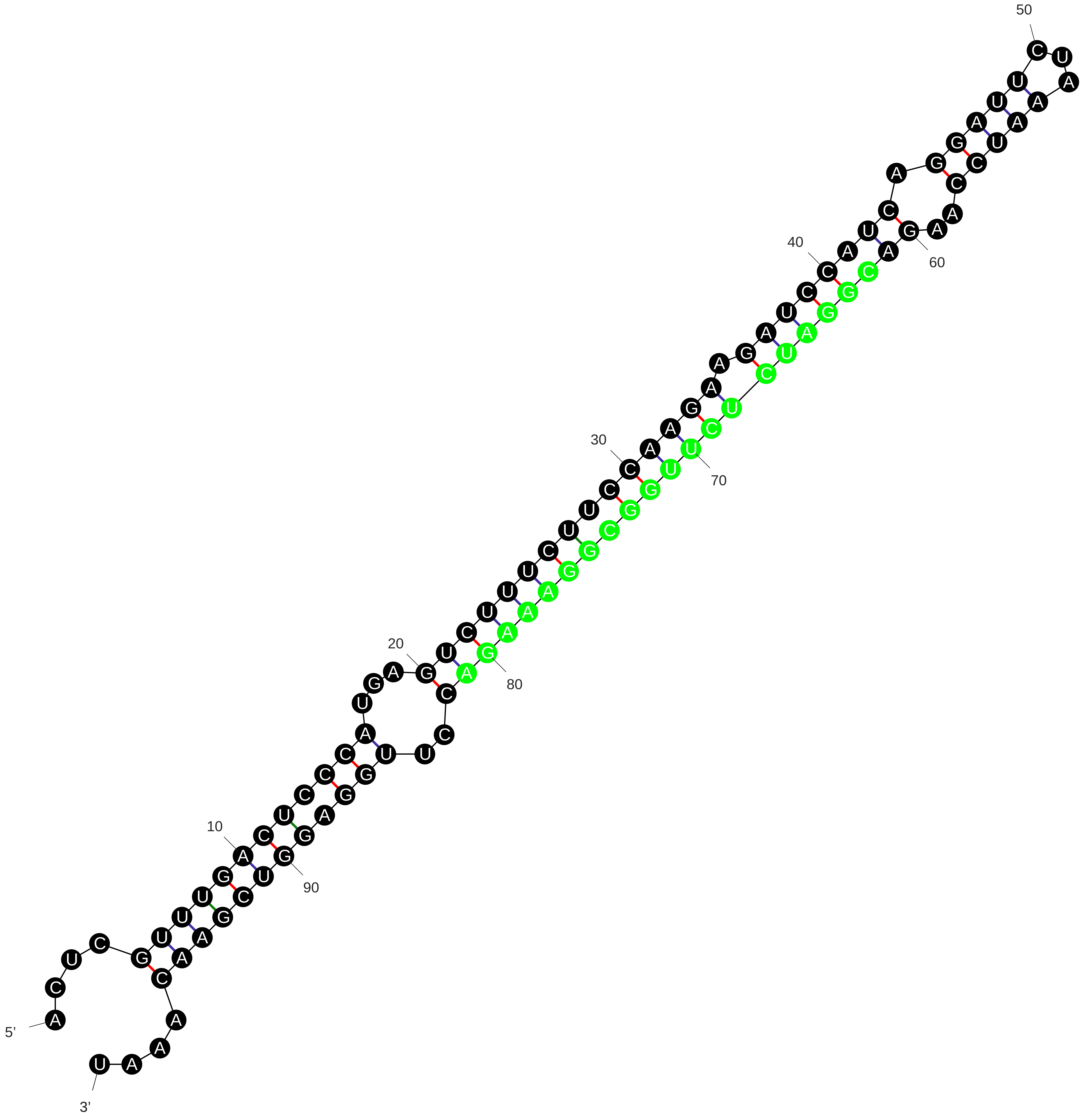

dG = -44.50 nta-miRn43

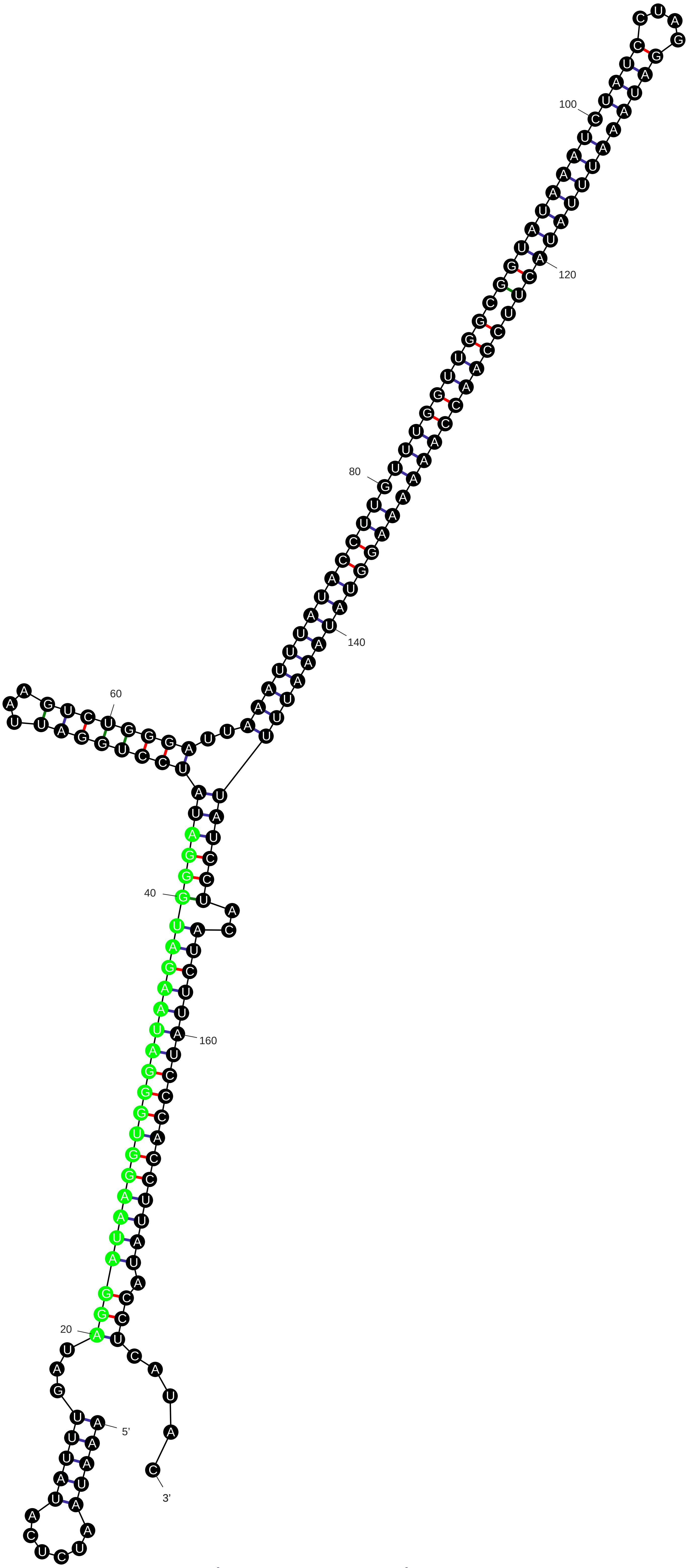

dG = -77.90 nta-miRn44

Output of sir_graph (©)
mfold_util 4.6

Created Mon Oct 17 23:19:11 2011

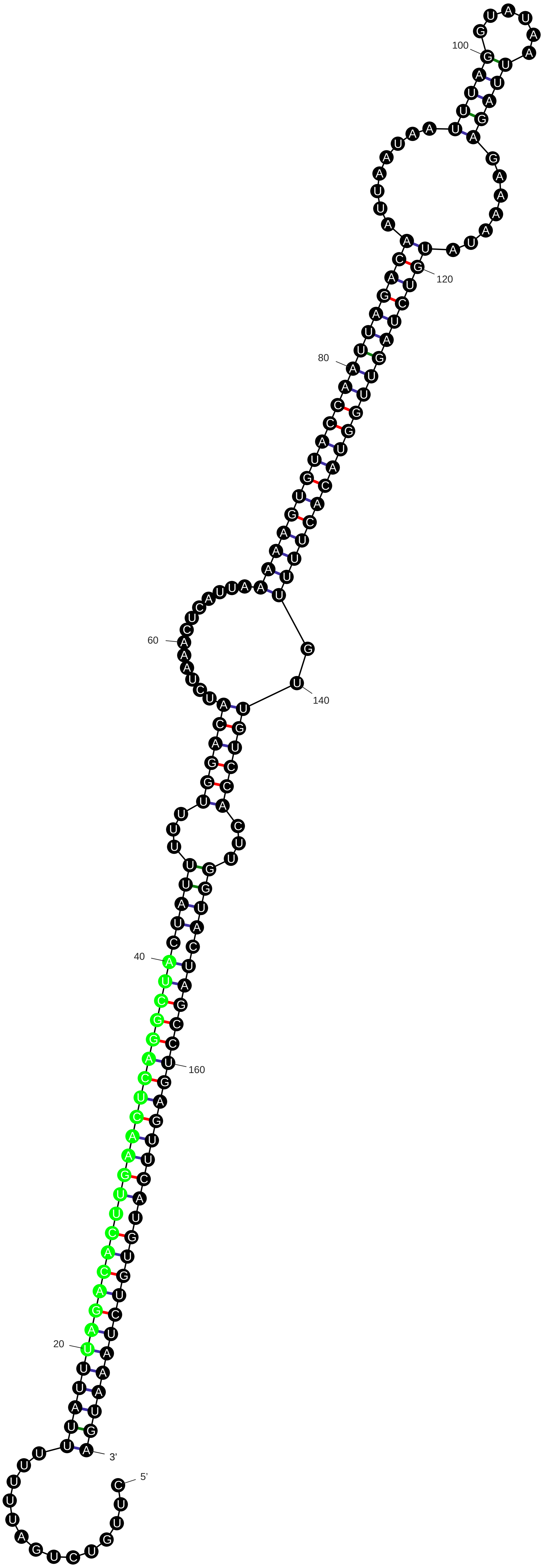

dG = -69.10 nta-miRn45

Output of sir_graph (©)
mfold_util 4.6

Created Mon Oct 17 23:21:11 2011

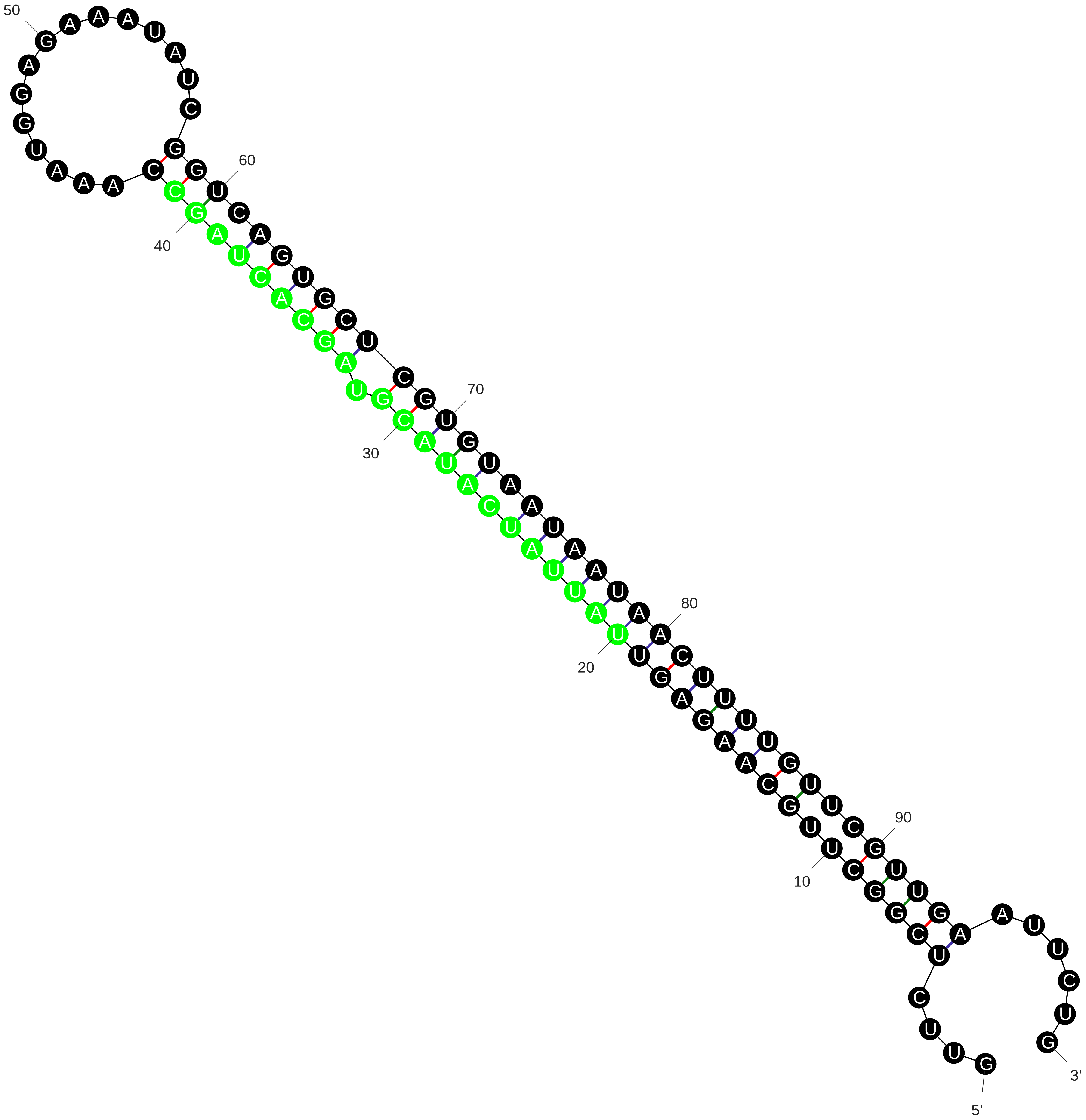

dG = -36.10 nta-miRn46

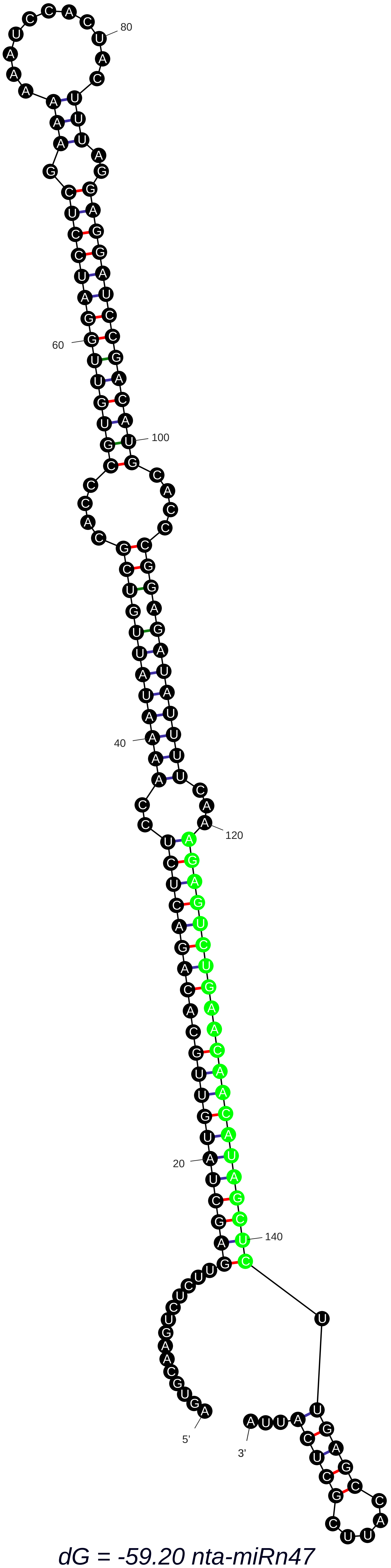

dG = -59.20 nta-miRn47

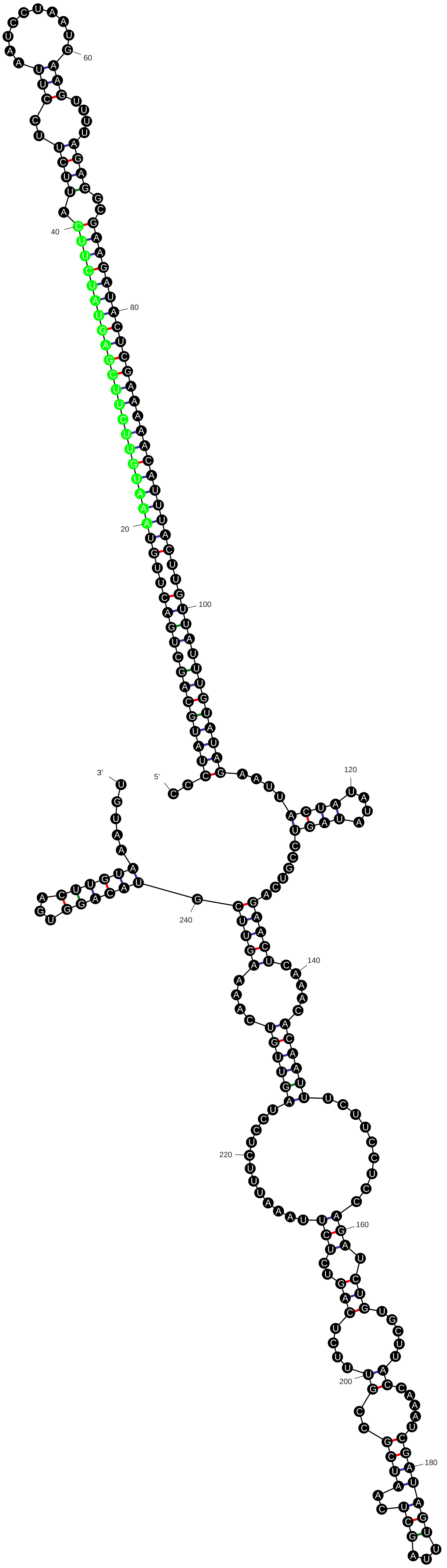

dG = -58.40 nta-miRn48

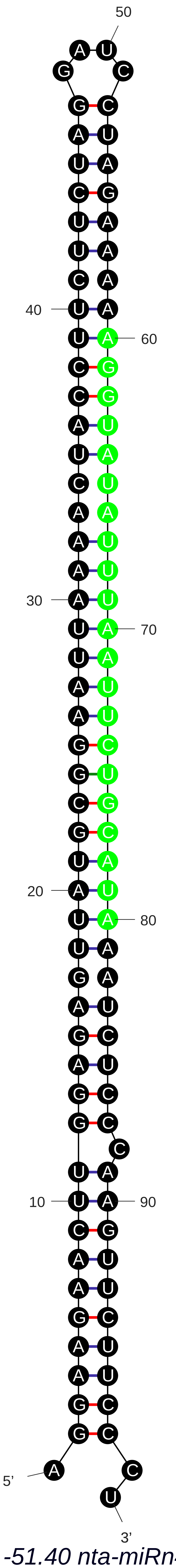

dG = -51.40 nta-miRn49

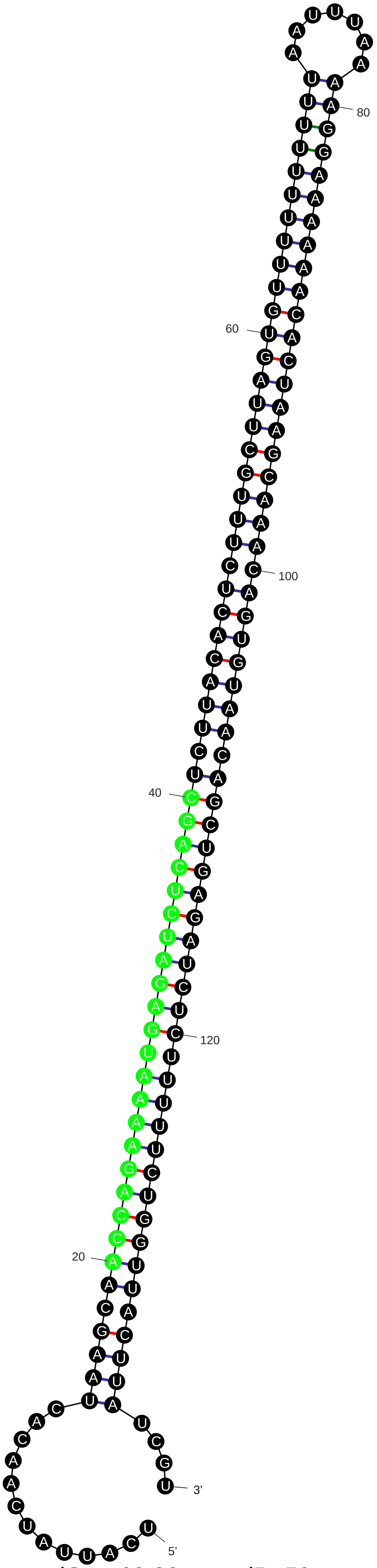

dG = -69.20 nta-miRn50

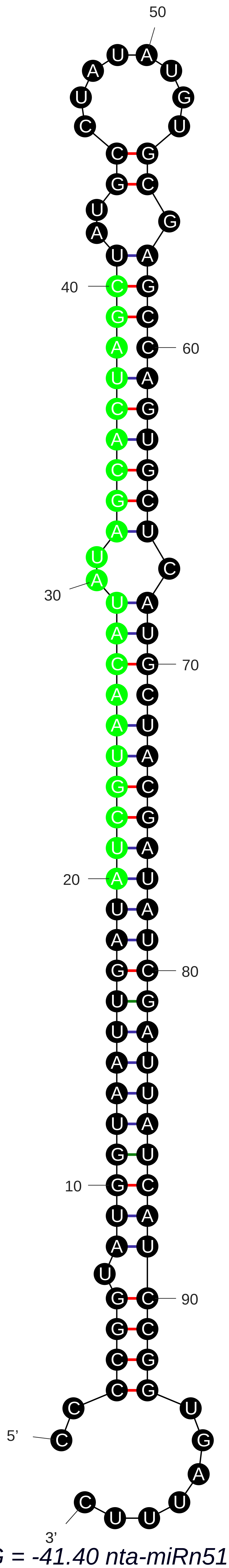

dG = -41.40 nta-miRn51

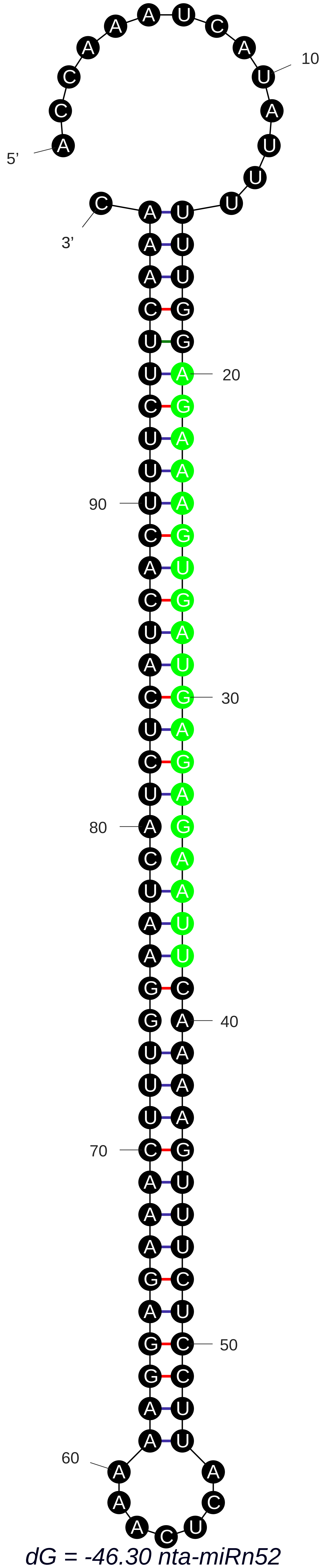

dG = -46.30 nta-miRn52

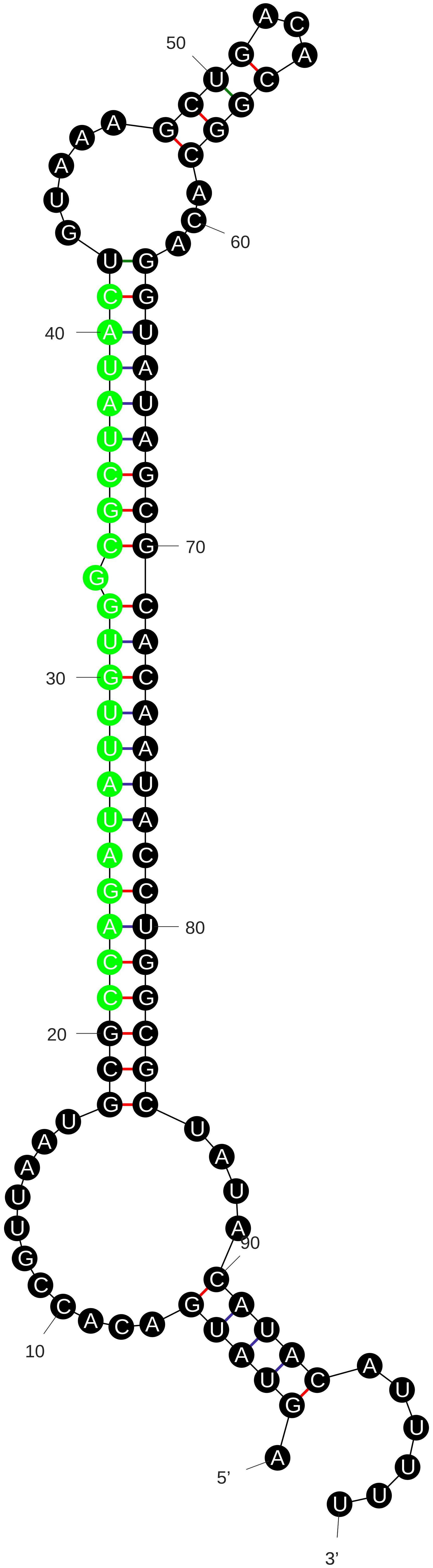

dG = -39.20 nta-miRn53

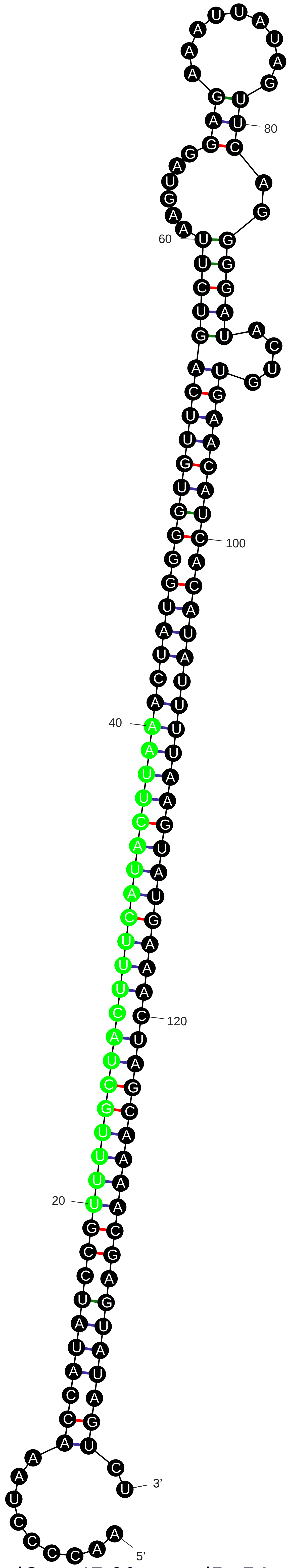

dG = -45.30 nta-miRn54

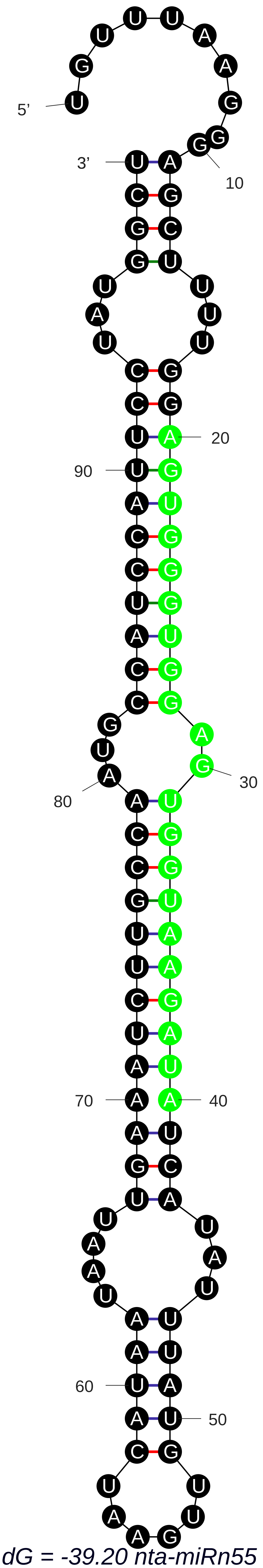

dG = -39.20 nta-miRn55

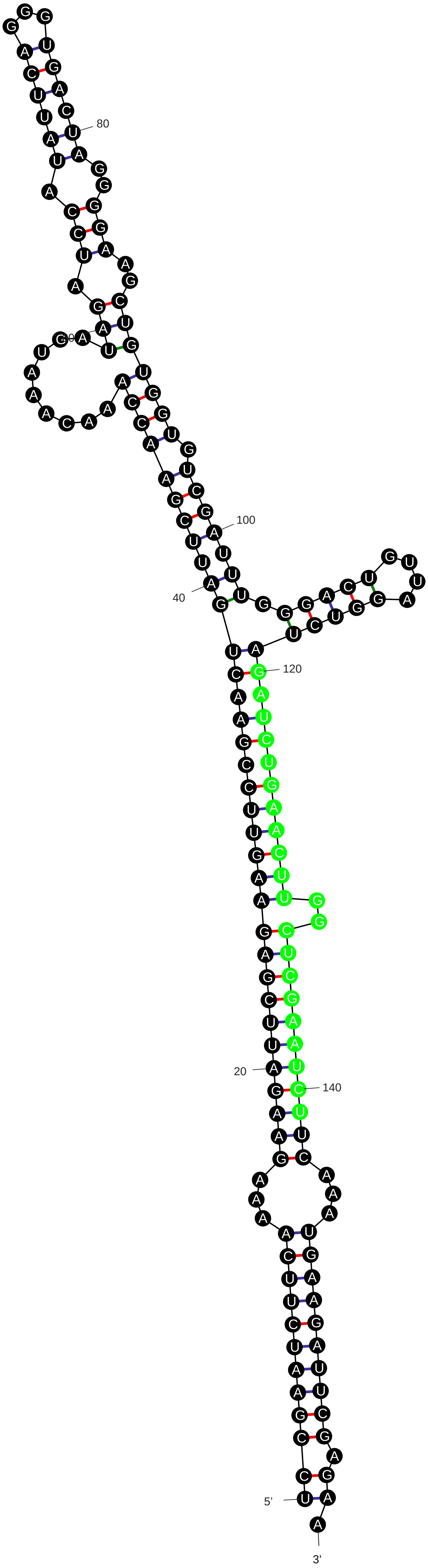

dG = -50.80 nta-miRn56

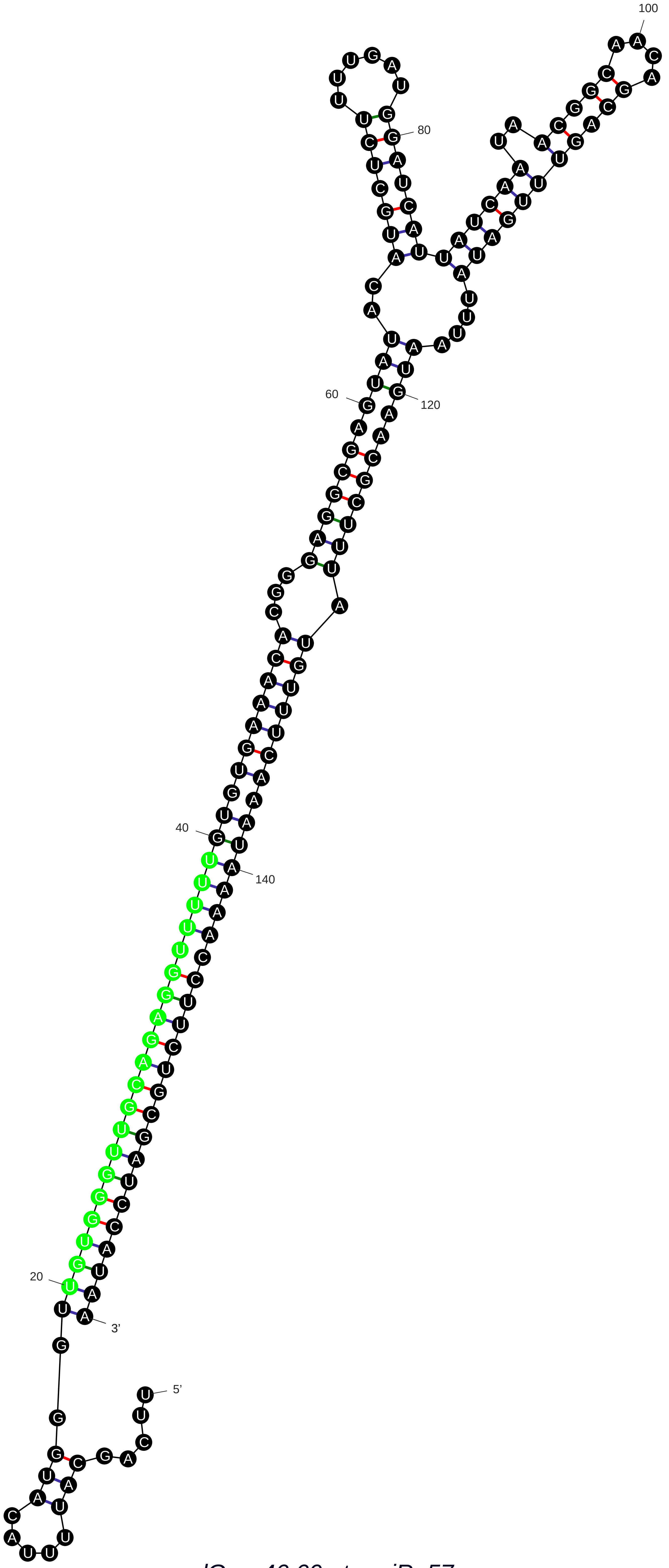

dG = -46.60 nta-miRn57

Output of sir_graph (©)
mfold_util 4.6

Created Mon Oct 17 23:46:04 2011

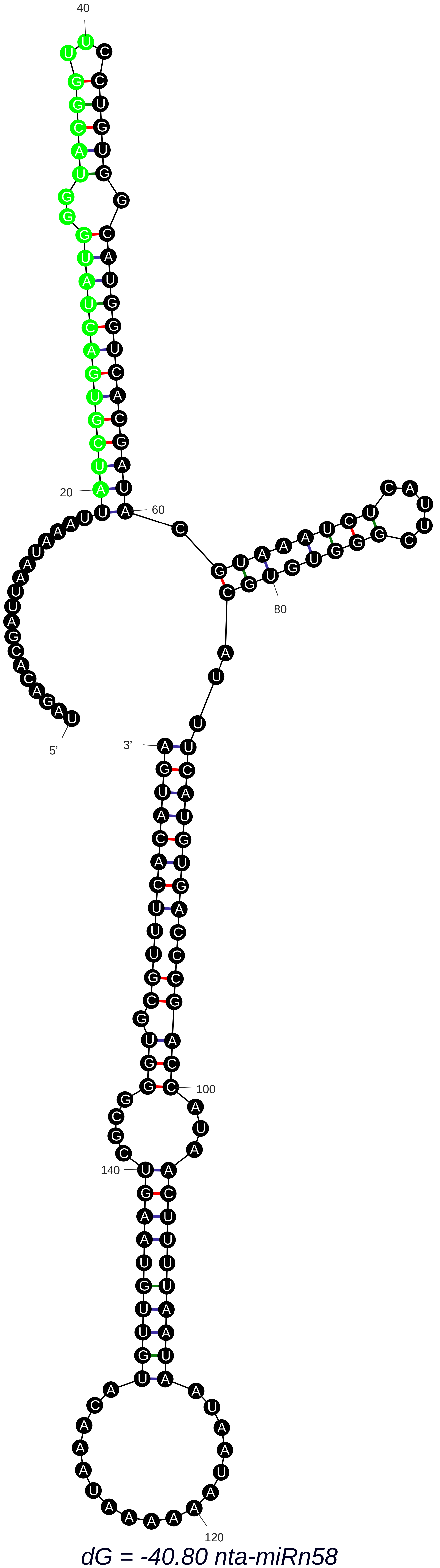

dG = -40.80 nta-miRn58

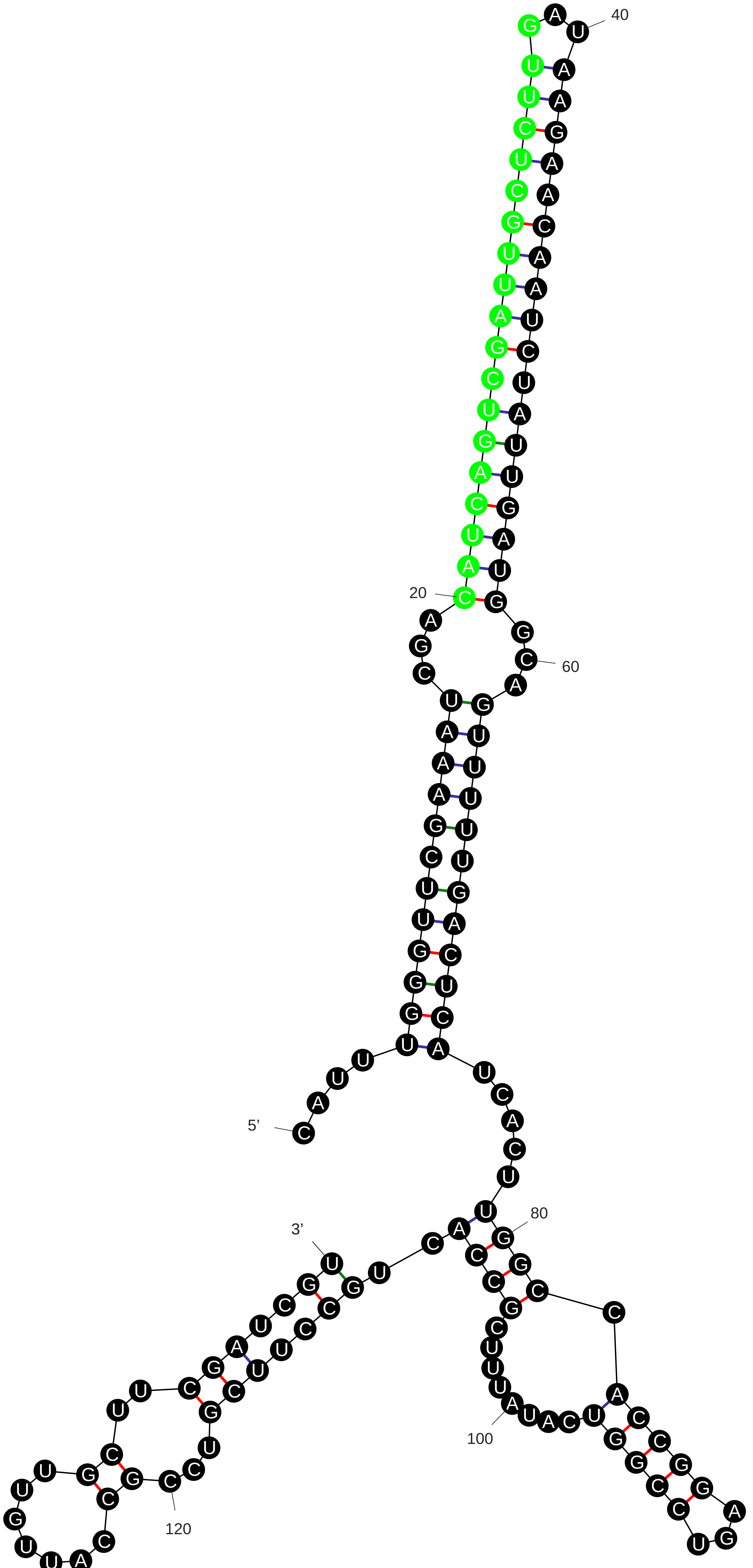

dG = -35.30 nta-miRn59

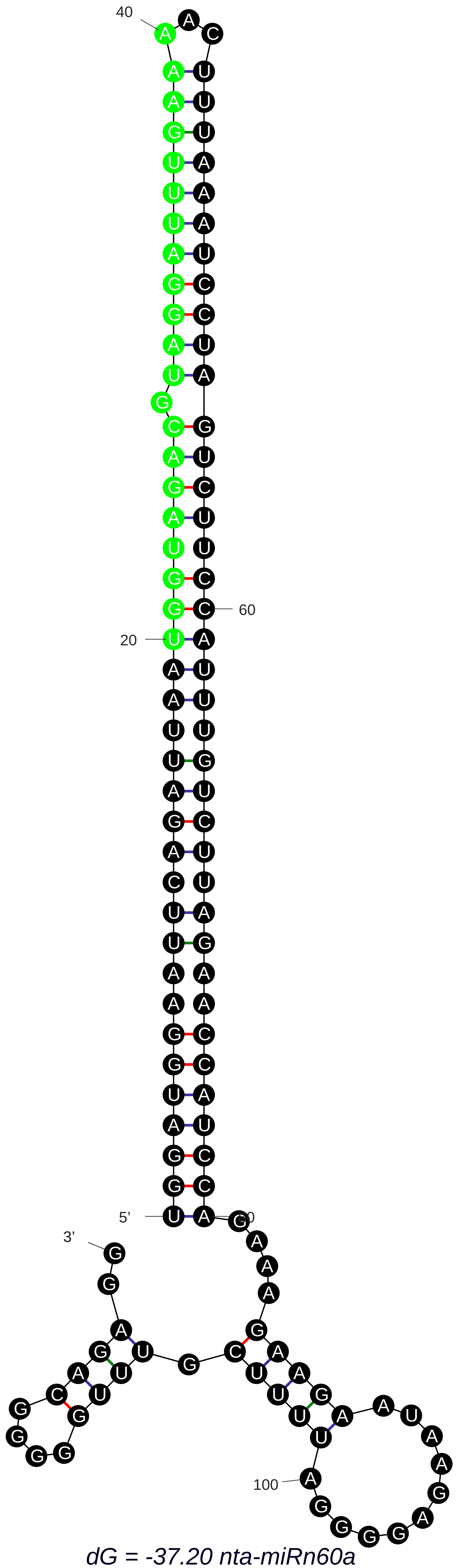

dG = -37.20 nta-miRn60a

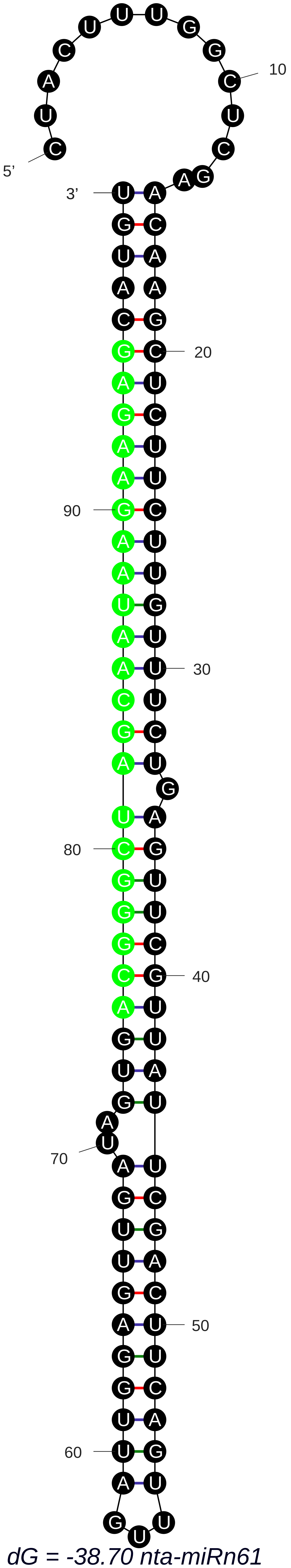

dG = -38.70 nta-miRn61

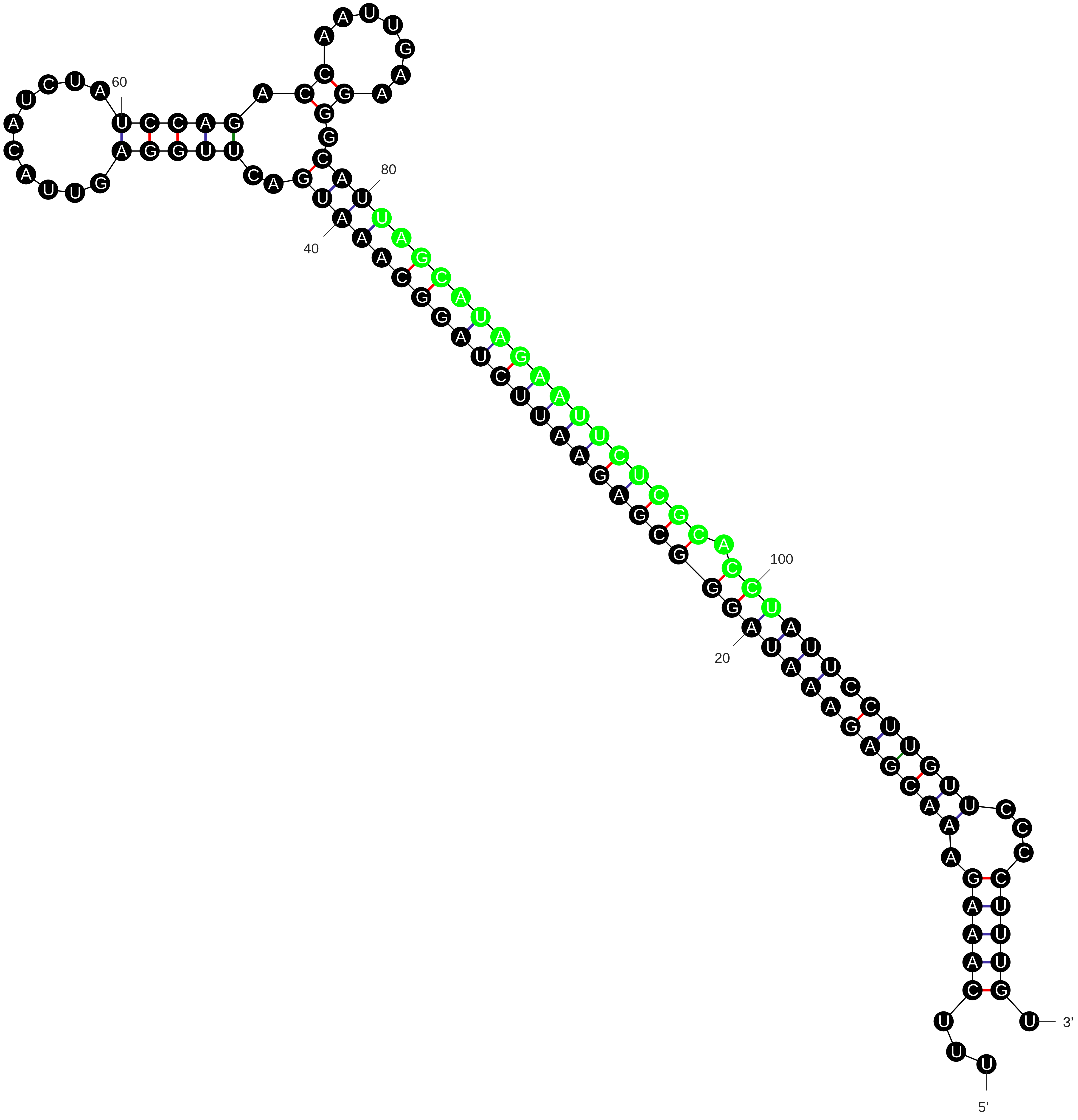

dG = -40.50 nta-miRn62

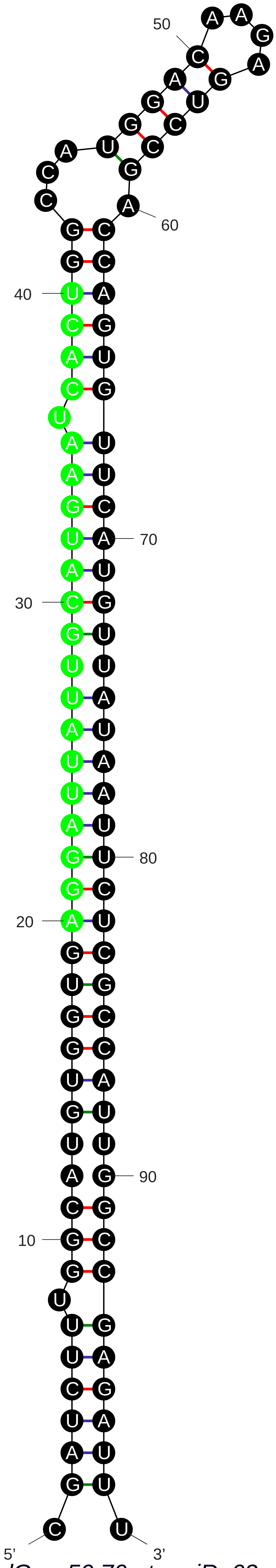

dG = -50.70 nta-miRn63

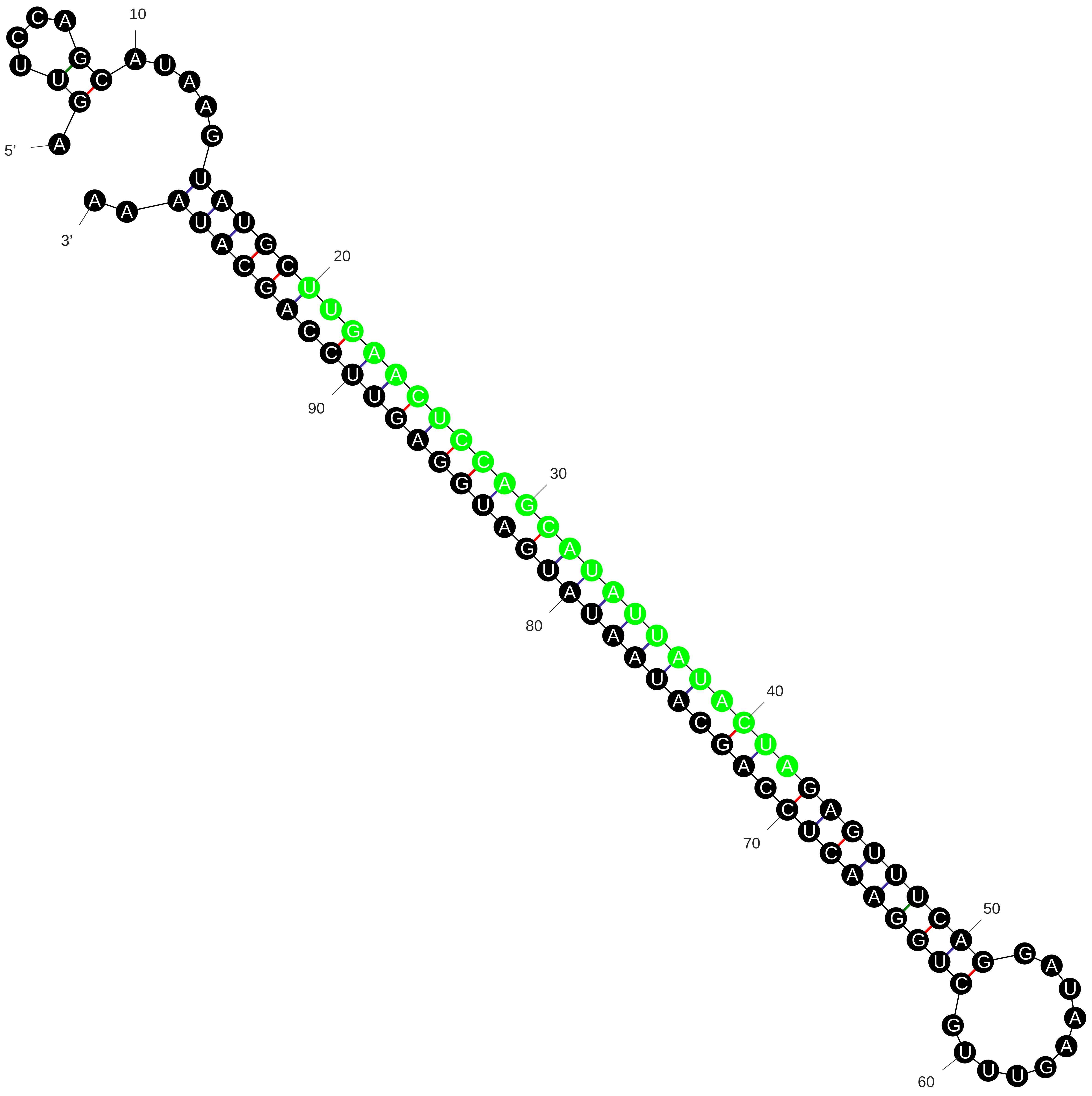

dG = -39.10 nta-miRn64

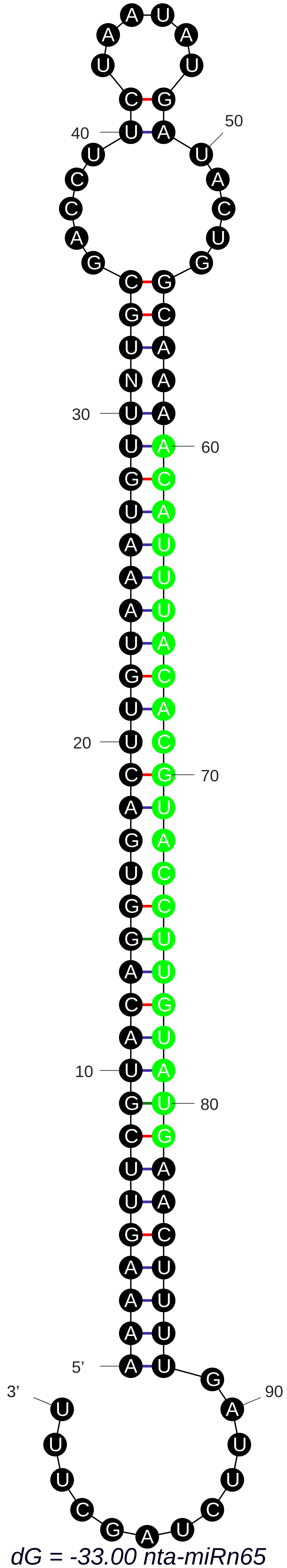

dG = -33.00 nta-miRn65

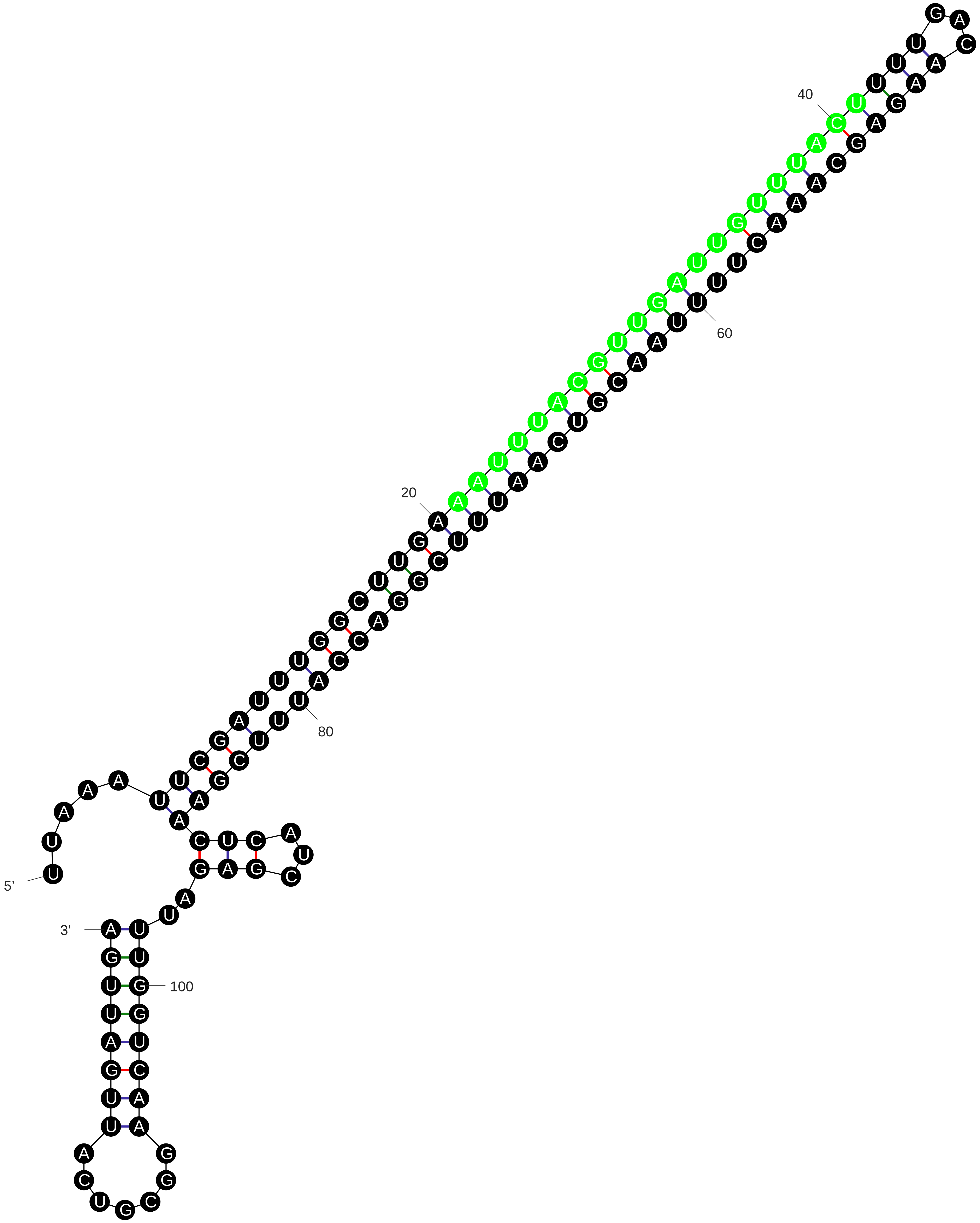

dG = -34.70 nta-miRn66

Output of sir_graph (©)
mfold_util 4.6

Created Tue Oct 18 00:03:57 2011

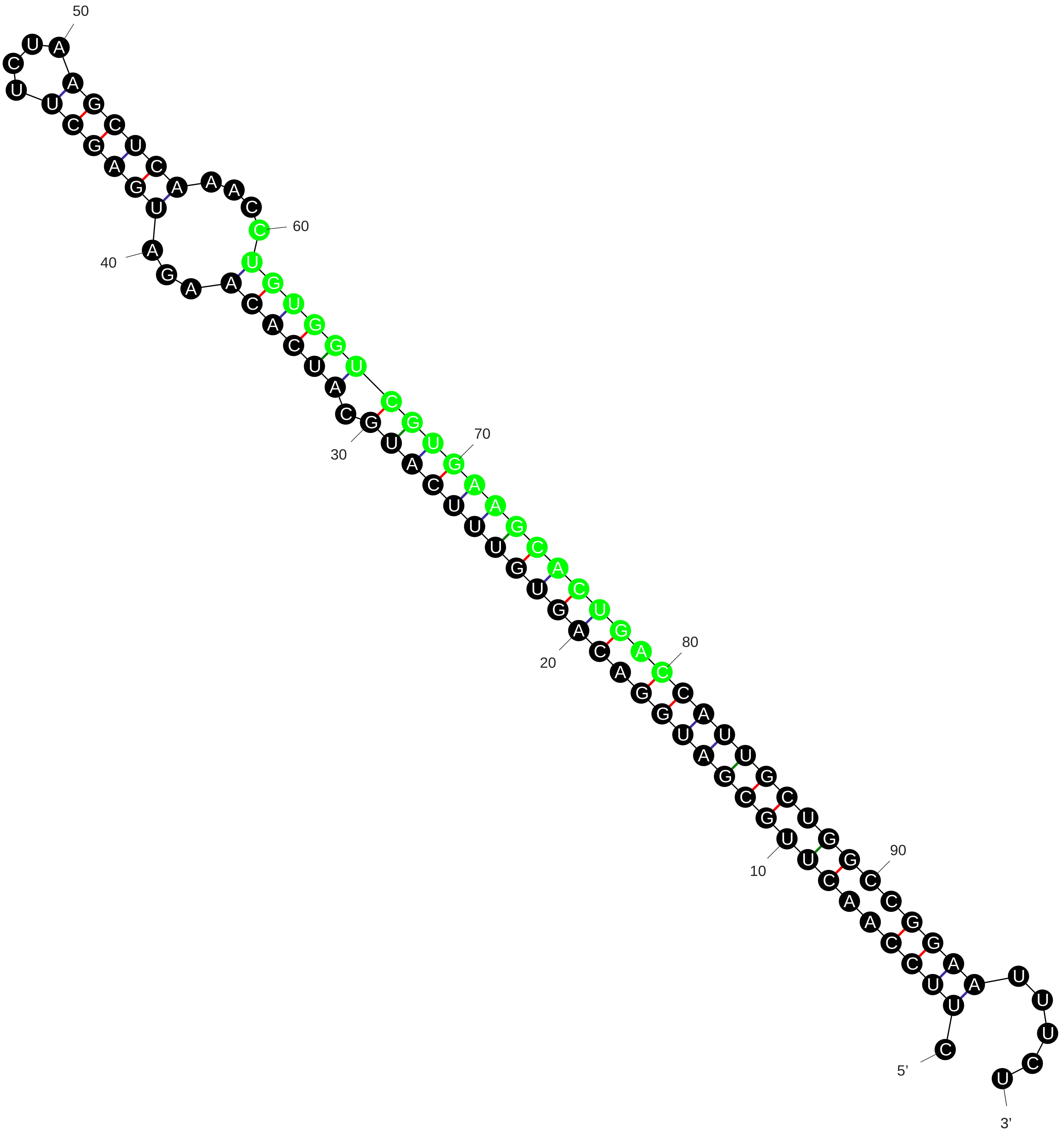

dG = -42.30 nta-miRn67

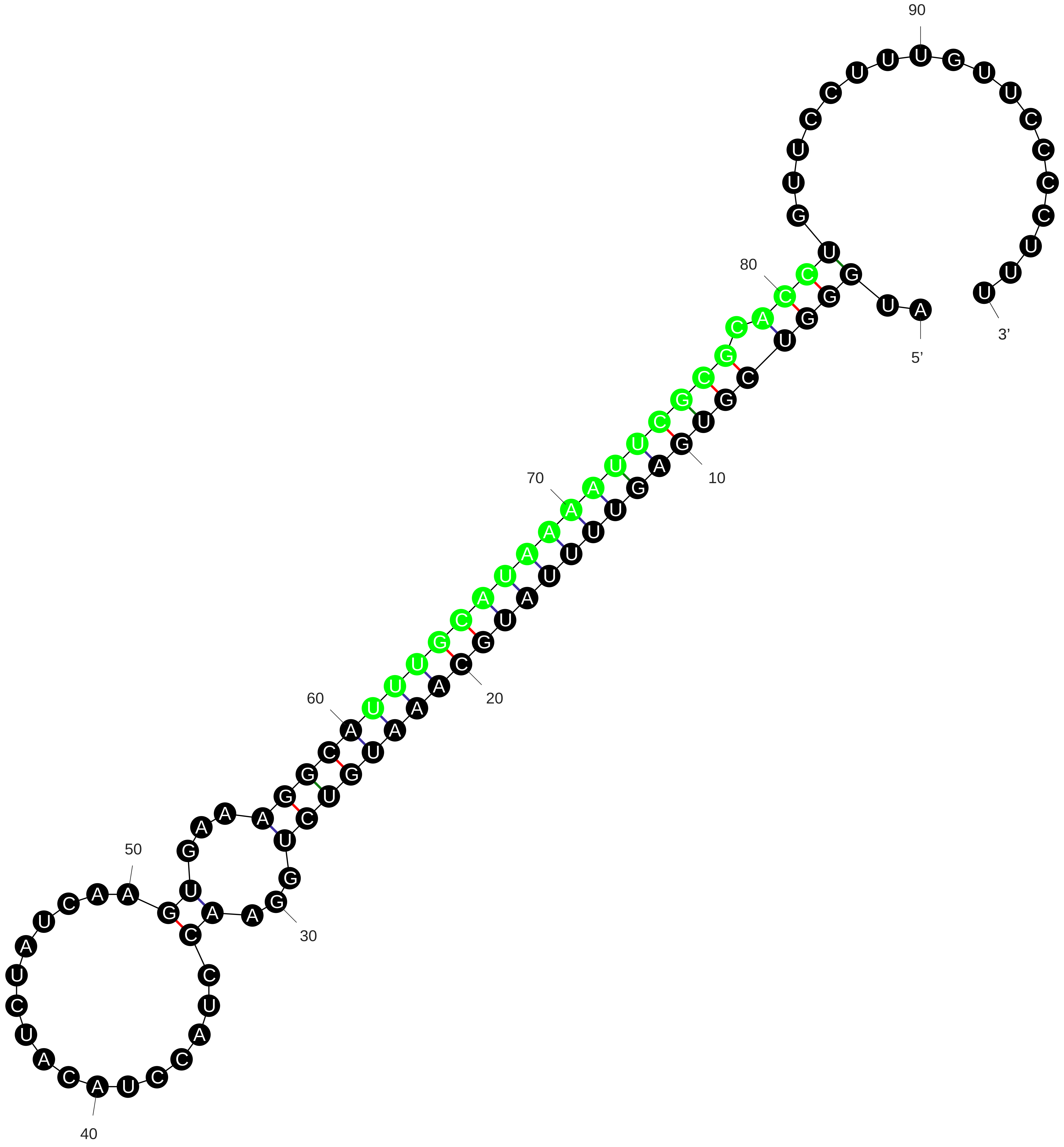

dG = -33.10 nta-miRn68

Output of sir_graph (©)
mfold_util 4.6

Created Tue Oct 18 00:08:17 2011

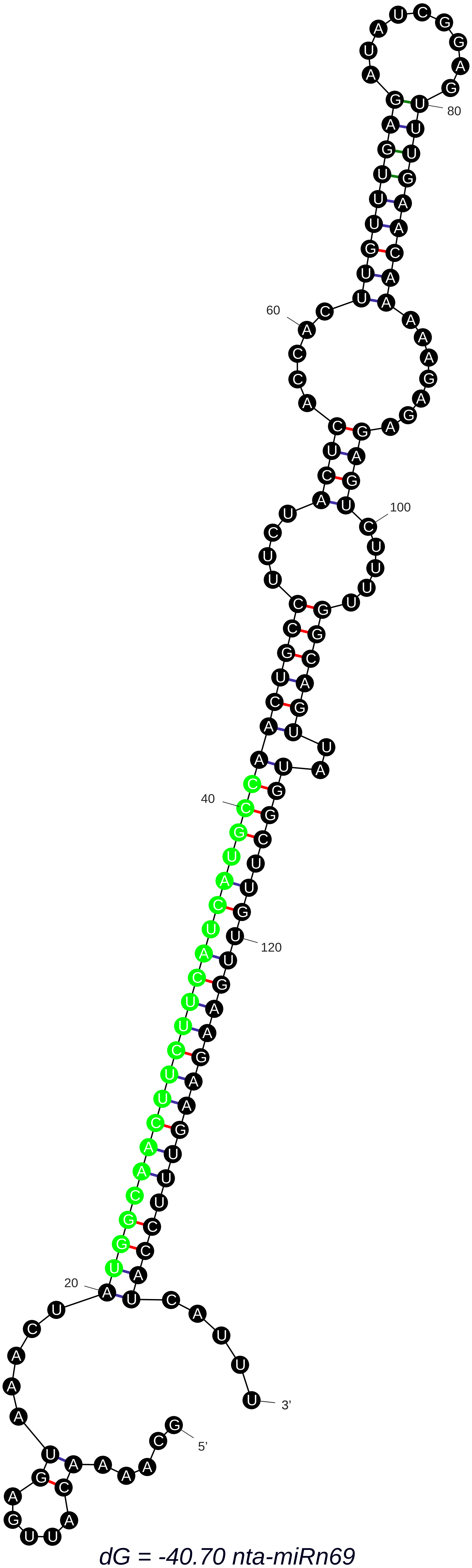

dG = -40.70 nta-miRn69

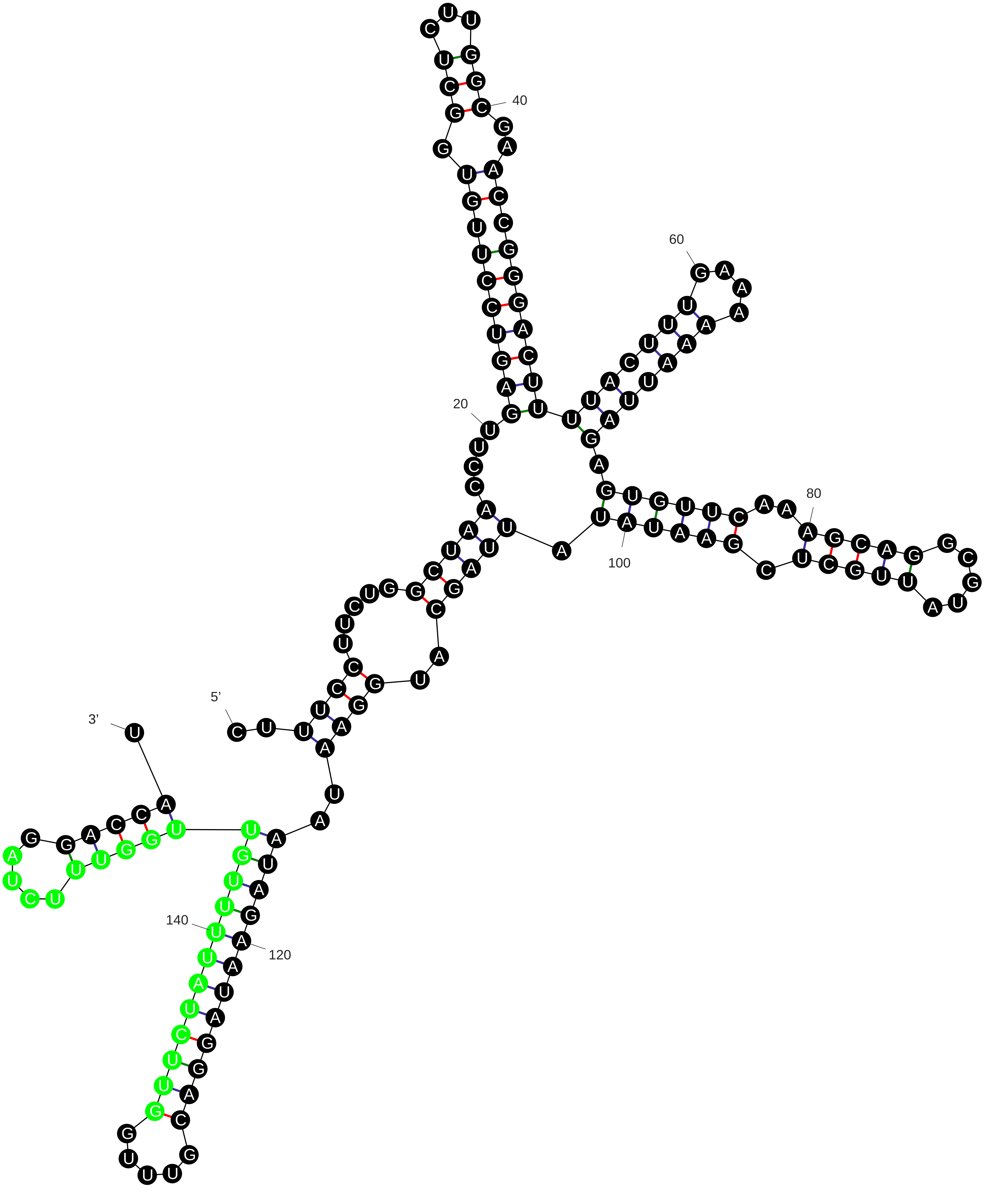

dG = -44.50 nta-miRn70

Output of sir_graph (©)
mfold_util 4.6

Created Tue Oct 18 00:12:36 2011

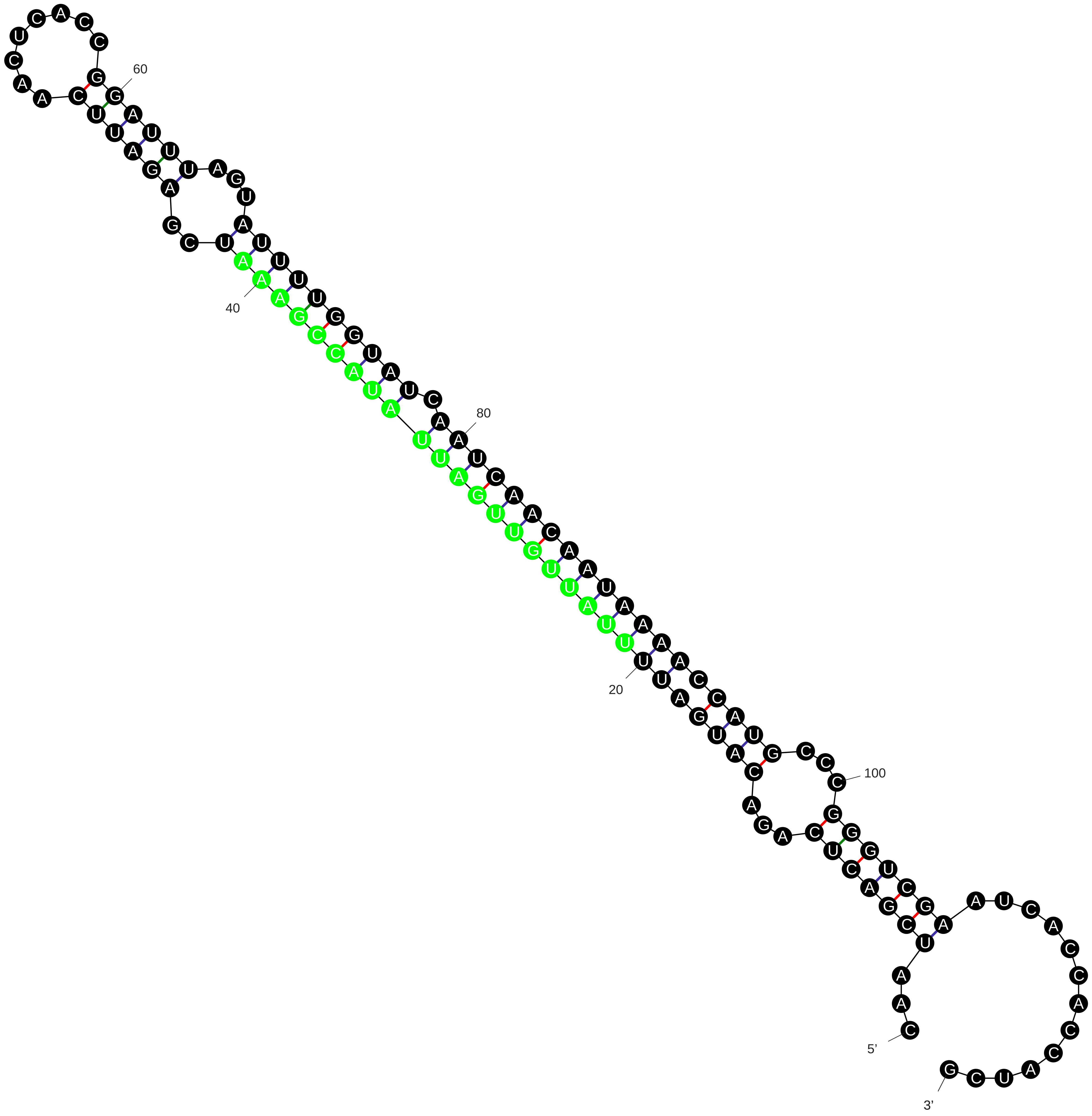

dG = -39.10 nta-miRn71

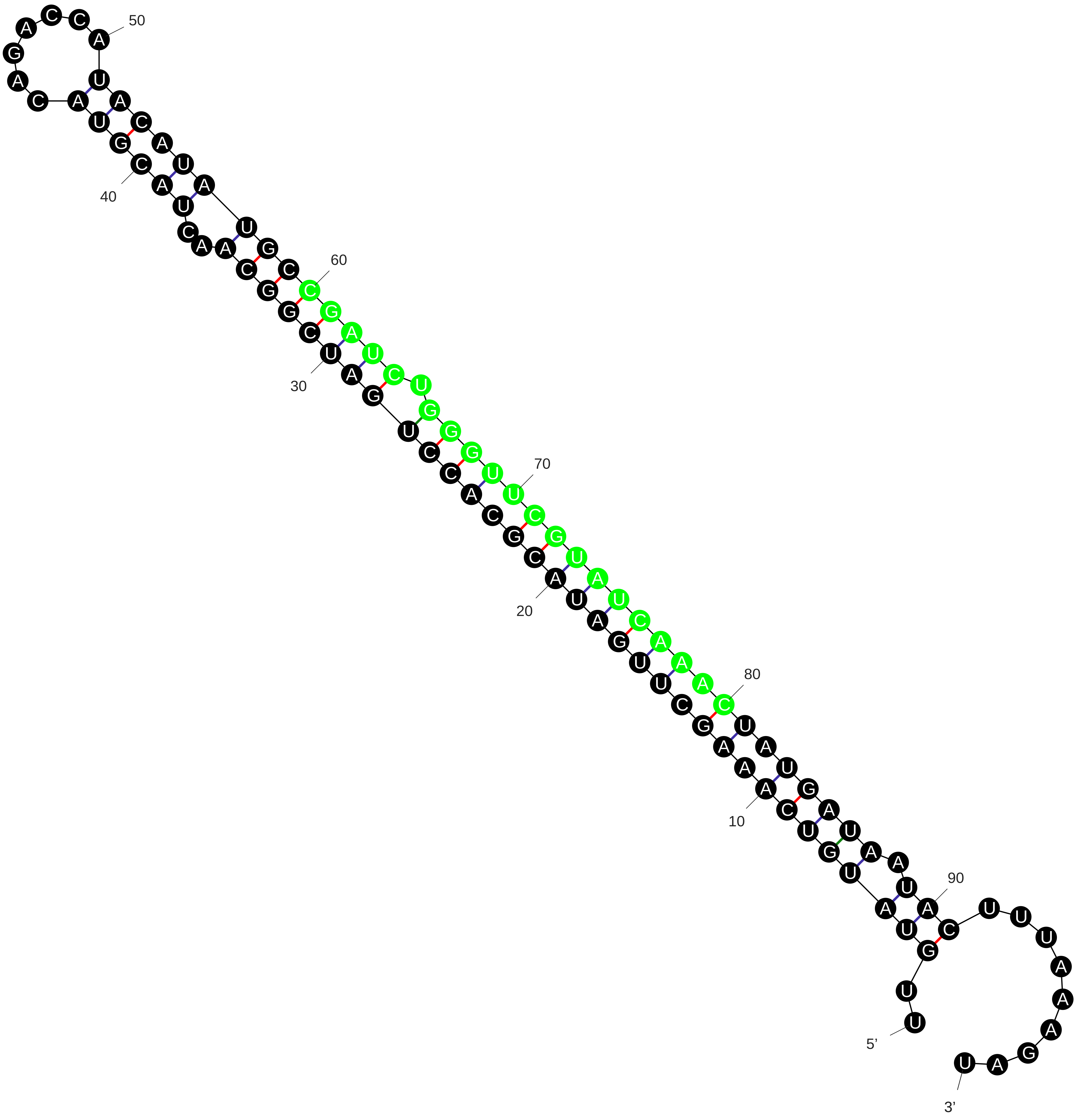

dG = -33.60 nta-miRn72

Output of sir_graph (©)
mfold_util 4.6

Created Tue Oct 18 00:16:27 2011

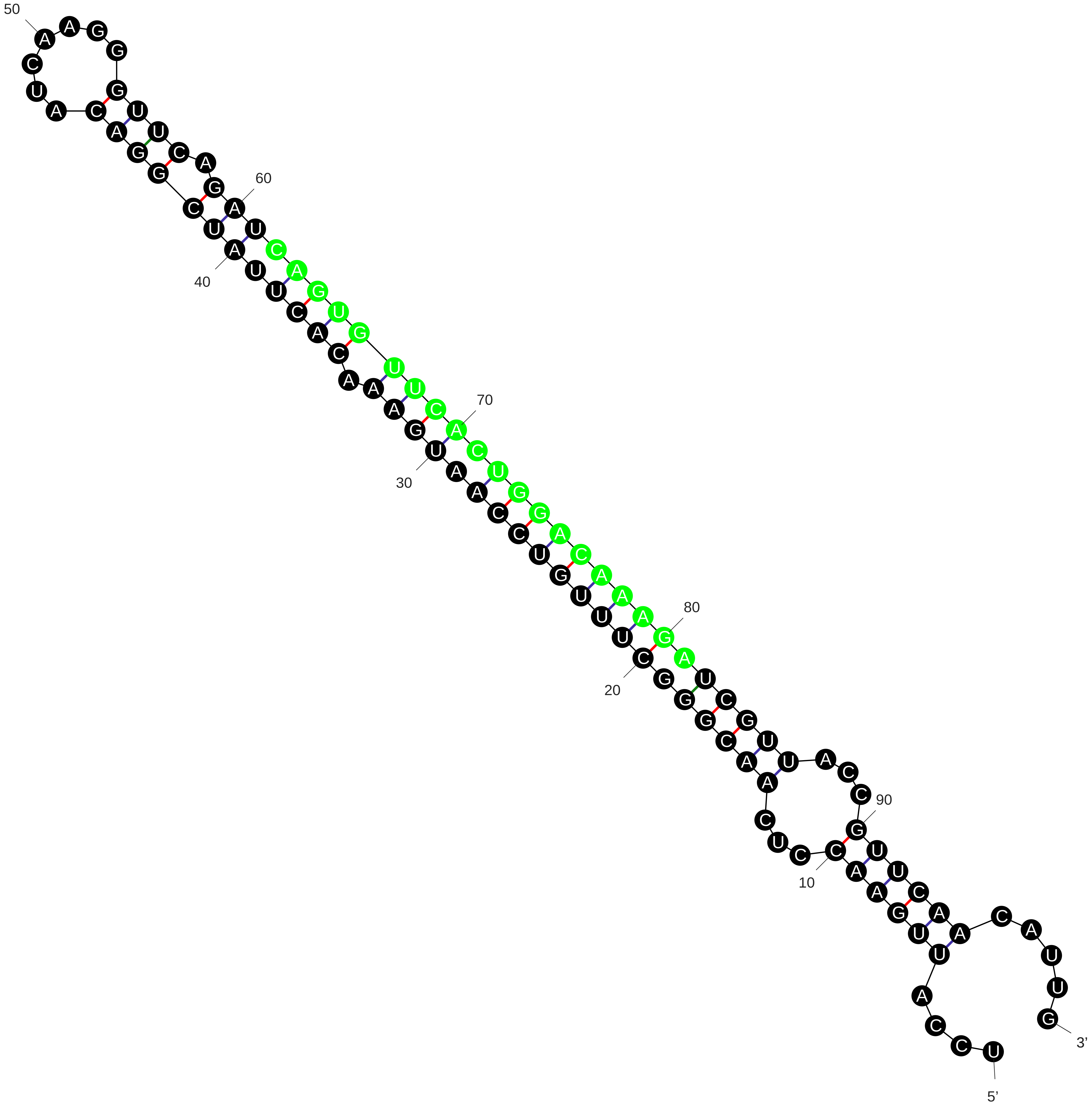

dG = -35.80 nta-miRn73

Output of sir_graph (©)
mfold_util 4.6

Created Tue Oct 18 00:19:03 2011

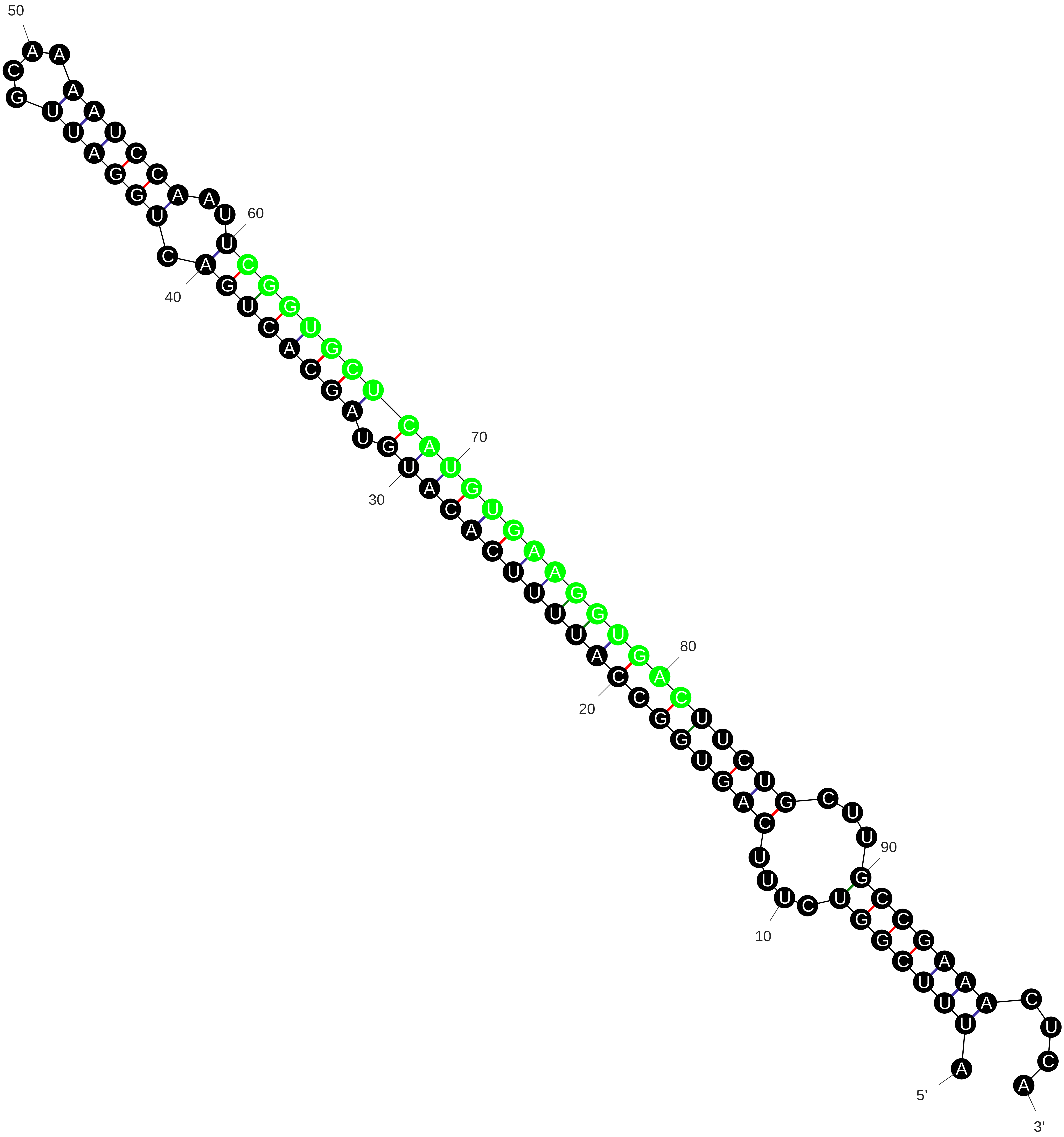

dG = -44.40 nta-miRn74

Output of sir_graph (©)
mfold_util 4.6

Created Tue Oct 18 00:20:58 2011

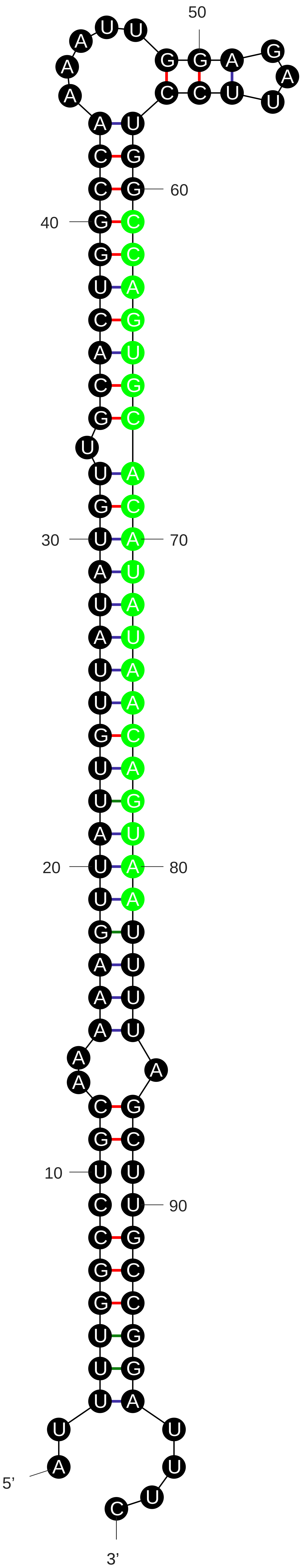

dG = -44.90 nta-miRn75

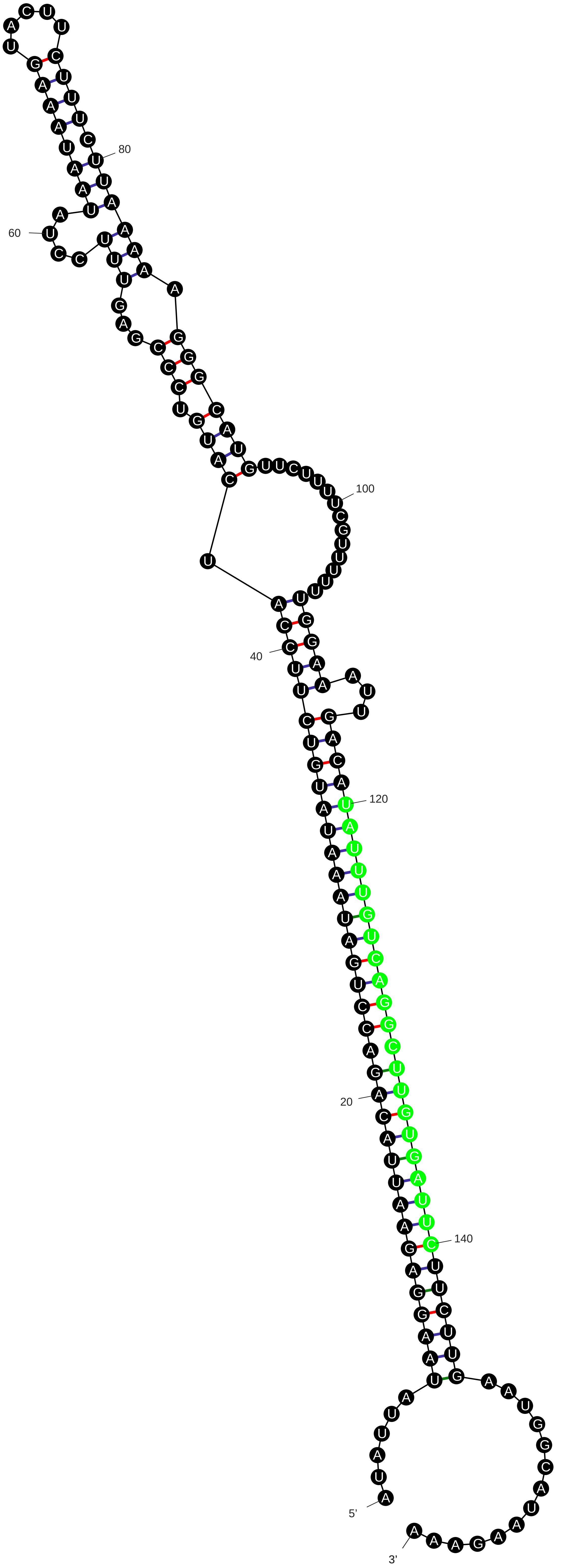

dG = -46.60 nta-miRn76

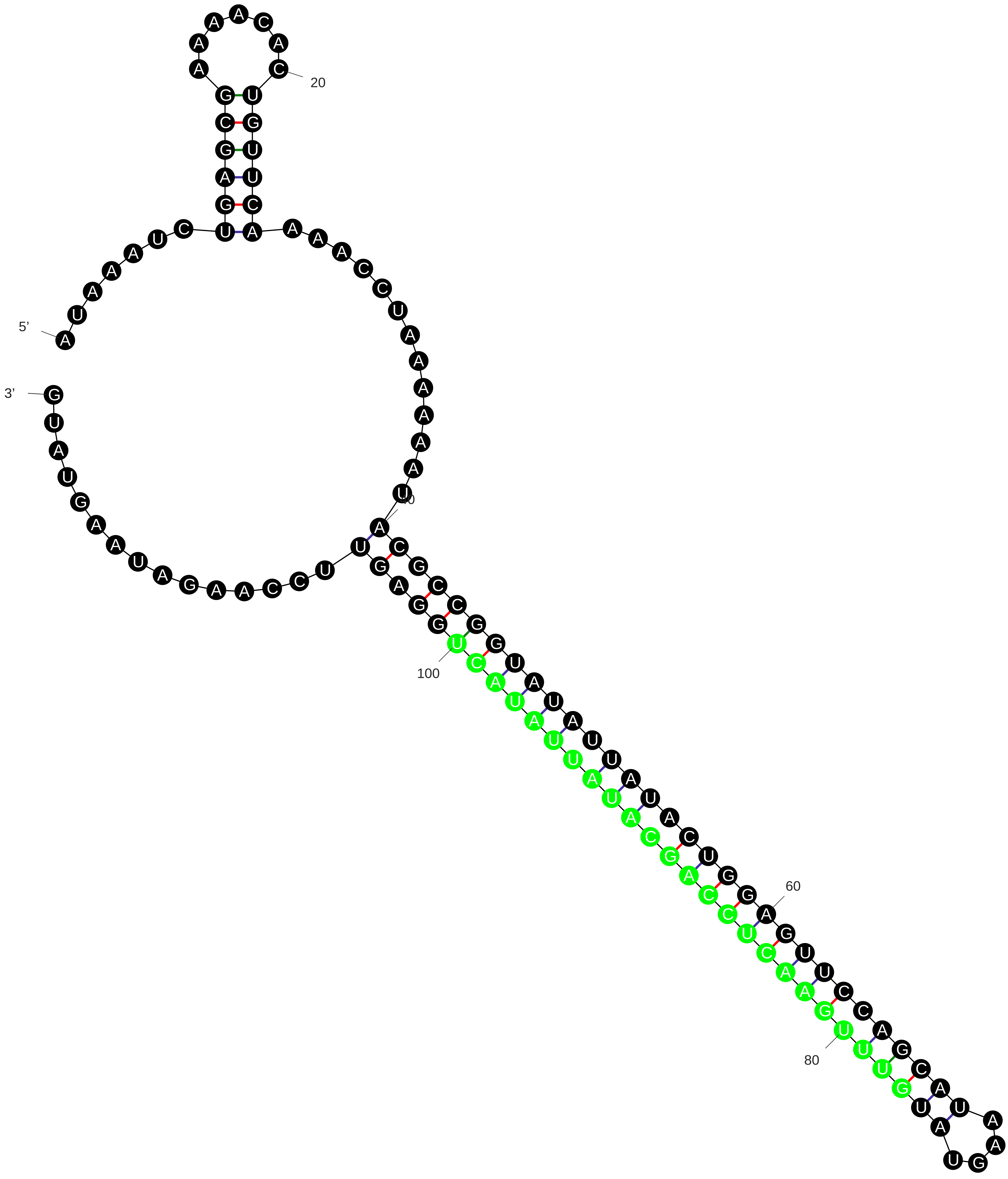

dG = -34.20 nta-miRn77a

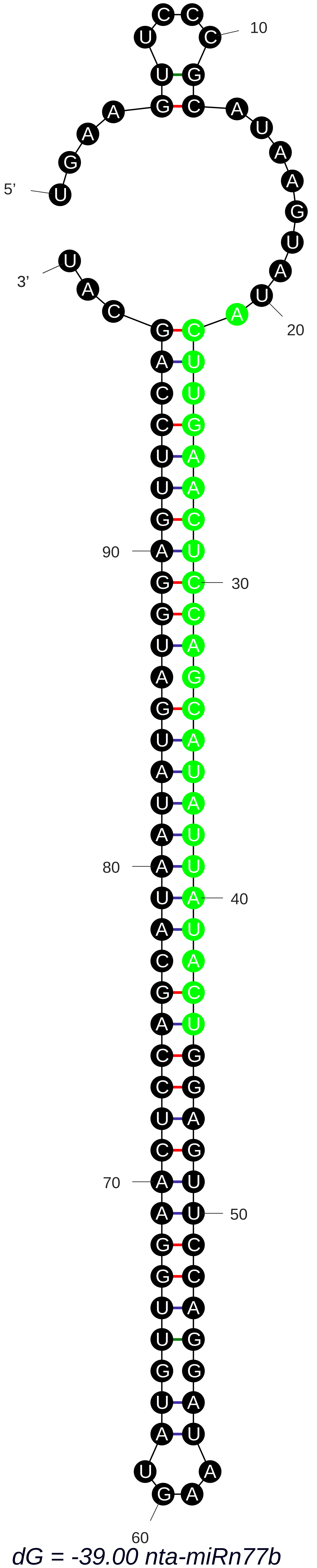

dG = -39.00 nta-miRn77b
